# Regioselective Pd-catalyzed direct C1- and C2-arylations of lilolidine for the access to 5,6-dihydropyrrolo[3,2,1-*ij*]quinoline derivatives

**DOI:** 10.3762/bjoc.15.204

**Published:** 2019-08-29

**Authors:** Hai-Yun Huang, Haoran Li, Thierry Roisnel, Jean-François Soulé, Henri Doucet

**Affiliations:** 1 Univ Rennes, CNRS, ISCR-UMR 6226, F-35000 Rennes, France

**Keywords:** catalysis: C–C bond formation, C–H bond activation, lilolidine, palladium

## Abstract

The Pd-catalyzed C–H bond functionalization of lilolidine was investigated. The use of a palladium-diphosphine catalyst associated to acetate bases in DMA was found to promote the regioselective arylation at α-position of the nitrogen atom of lilolidine with a wide variety of aryl bromides. From these α-arylated lilolidines, a second arylation at the β-position gives the access to α,β-diarylated lilolidines containing two different aryl groups. The one pot access to α,β-diarylated lilolidines with two identical aryl groups is also possible by using a larger amount of aryl bromide. The synthesis of 5,6-dihydrodibenzo[*a*,*c*]pyrido[3,2,1-*jk*]carbazoles from lilolidine via three successive direct arylations is also described. Therefore, this methodology provides a straightforward access to several lilolidine derivatives from commercially available compounds via one, two or three C–H bond functionalization steps allowing to tune their biological properties.

## Introduction

**Figure 1:**
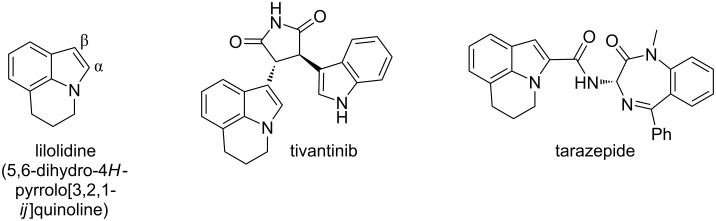
Structures of lilolidine, tivantinib and tarazepide.

Lilolidine (Figure 1, left), which is a commercially available compound, contains a 5,6-dihydropyrrolo[3,2,1-*ij*]quinoline skeleton found in several bioactive molecules. For example, tivantinib (Figure 1, middle) exhibits MET inhibitor properties [1]; whereas tarazepide (Figure 1, right) is being investigated for the treatment of gastrointestinal diseases. Other lilolidine derivatives also exhibit properties for the treatment of cancers [2–5]. Therefore, the discovery of simple methods for the preparation of lilolidine derivatives is an important research area in pharmaceutical chemistry.

**Scheme 1:**
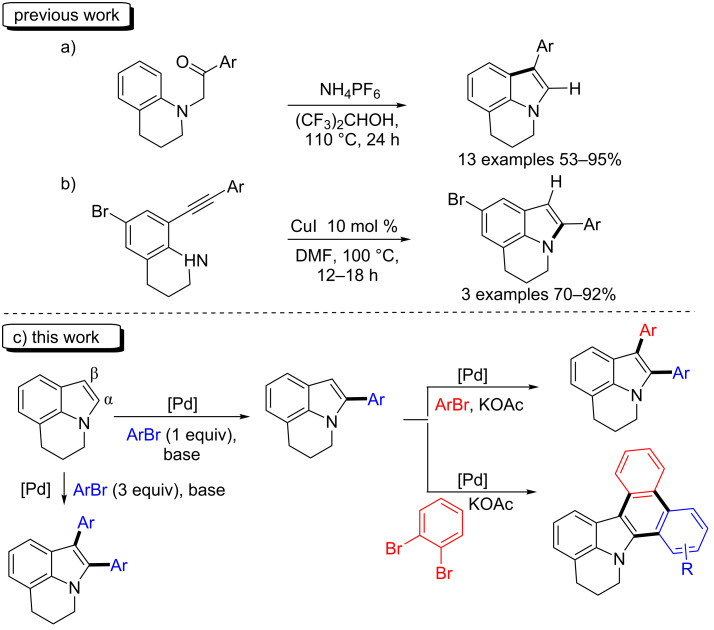
Access to α- and β-arylated lilolidine derivatives.

To the best of our knowledge, so far only a few methods allow the synthesis of lilolidines arylated at α- [6–7] or β- [8–13] positions of the nitrogen atom. In 2014, Chen and Tang et al. reported that NH_4_PF_6_ promotes the cyclodehydration of α-aminocarbonyl compounds, leading to the formation of β-arylated 5,6-dihydropyrrolo[3,2,1-*ij*]quinoline derivatives [6] (Scheme 1a). Three α-arylated 5,6-dihydropyrrolo[3,2,1-*ij*]quinoline derivatives have been prepared by Pal et al. via the cyclization of 8-arylethynyl-1,2,3,4-tetrahydroquinolines [9] (Scheme 1b). The best results were obtained using 10 mol % of CuI as catalyst in DMF at 100 °C.

The late stage C–H bond functionalization of molecules represents a powerful method for the easy screening of the biological properties of compounds containing a bioactive unit. Since the seminal work by Ohta et al. on the Pd-catalyzed C–H bond functionalization of heteroarenes such as thiophenes, furans, pyrroles and indoles [14–15], this methodology has been widely applied for the preparation of new aryl-substituted heteroarenes [16–21]. Several results dealing with the C–H bond functionalization of indoles have been reported allowing to prepare either α- [22–31] or β-arylated [32–37] indoles, depending on the reaction conditions. However, to the best of our knowledge, no example of regioselective α- or β-arylations via the C–H bond functionalization of lilolidine has been reported so far (Scheme 1c).

Herein, we report i) on the simple access to α-arylated 5,6-dihydropyrrolo[3,2,1-*ij*]quinolines using an air-stable Pd catalyst associated to an inexpensive base, ii) on the sequential access to α,β-diarylated 5,6-dihydropyrrolo[3,2,1-*ij*]quinolines containing identical or different aryl groups at α- and β-positions via two-fold Pd-catalyzed C–H bond functionalizations, iii) on the synthesis of 5,6-dihydrodibenzo[*a*,*c*]pyrido[3,2,1-*jk*]carbazoles via three successive C–H bond functionalization steps (Scheme 1c).

## Results and Discussion

Based on our previous results on the arylation of heteroaromatics [38], we initially employed 1 mol % Pd(OAc)_2_ catalyst associated to KOAc as the base in DMA at 150 °C as the reaction conditions to promote the coupling of lilolidine with 3-bromobenzonitrile (Table 1, entry 1). Under these conditions a mixture of the α- and β-arylated lilolidines **1a** and **1b** was obtained in a 64:36 ratio. Then, the influence of some bases on the regioselectivity with Pd(OAc)_2_ catalyst was examined. With CsOAc a similar regioselectivity than with KOAc was obtained, whereas the use of NaOAc afforded the products **1a** and **1b** in an 85:15 ratio, but with a moderate conversion of 3-bromobenzonitrile (Table 1, entries 2 and 3). High regioselectivities in favor of isomer **1a** (85–90%) were also obtained using K_2_CO_3_ and Na_2_CO_3_, but partial conversions of 3-bromobenzonitrile were observed (Table 1, entries 4–6). In order to improve the conversions of the aryl bromide, the thermally more stable PdCl(C_3_H_5_)(dppb) catalyst [39] was employed. With K_2_CO_3_ and Na_2_CO_3_, the conversion of 3-bromobenzonitrile was not improved, whereas using NaOAc, a complete conversion of the aryl bromide was observed (Table 1, entries 8–10). Moreover, the regioselectivity in favor of α-arylated lilolidine was improved to 93% affording **1a** in 83% yield. The use of KOAc associated to PdCl(C_3_H_5_)(dppb) catalyst also afforded the regioisomer **1a** in a quite good regioselectivity and yield (Table 1, entry 11). The higher conversions observed in the presence of acetate bases compared to carbonate bases (Table 1, entries 4–6 and 8–11) might be due to an easier coordination of acetates to palladium which favors the concerted metallation deprotonation (CMD) mechanism [40]. The regioselectivities observed using acetate bases are consistent with a CMD mechanism.

**Table 1:** Influence of the reaction conditions on the palladium-catalyzed direct coupling of lilolidine with 3-bromobenzonitrile.^a^

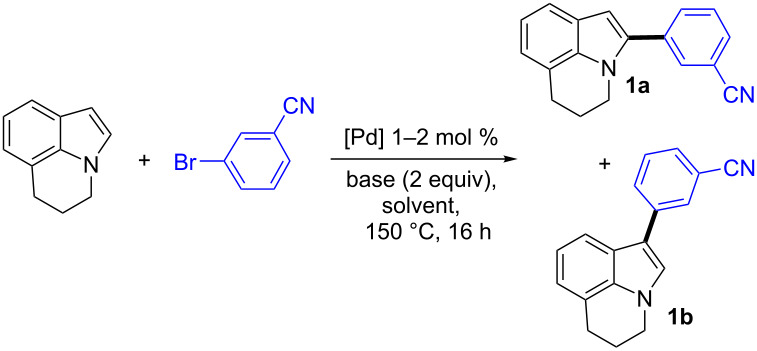					

Entry	Catalyst (mol %)	Base	Conv. (%)	Ratio **1a**:**1b**	Yield in **1a** (%)

1	Pd(OAc)_2_ (1)	KOAc	100	64:36	63
2	Pd(OAc)_2_ (1)	CsOAc	100	69:31	58
3	Pd(OAc)_2_ (1)	NaOAc	65	85:15	43
4	Pd(OAc)_2_ (1)	K_2_CO_3_	40	90:10	37
5	Pd(OAc)_2_ (1)	Cs_2_CO_3_	0	–	–
6	Pd(OAc)_2_ (1)	Na_2_CO_3_	33	85:15	n.d.
7	Pd(OAc)_2_ (1)	KOAc	0^b^	–	–
8	PdCl(C_3_H_5_)(dppb) (2)	K_2_CO_3_	42	90:10	n.d.
9	PdCl(C_3_H_5_)(dppb) (2)	Na_2_CO_3_	35	91:9	n.d.
10	PdCl(C_3_H_5_)(dppb) (2)	NaOAc	100	93:7	83
11	PdCl(C_3_H_5_)(dppb) (2)	KOAc	100	82:18	68

^a^Conditions: lilolidine (1.5 mmol), 3-bromobenzonitrile (1 mmol), base (2 mmol), DMA, under argon, 16 h, 150 °C, isolated yields. ^b^In xylene.

**Scheme 2:**
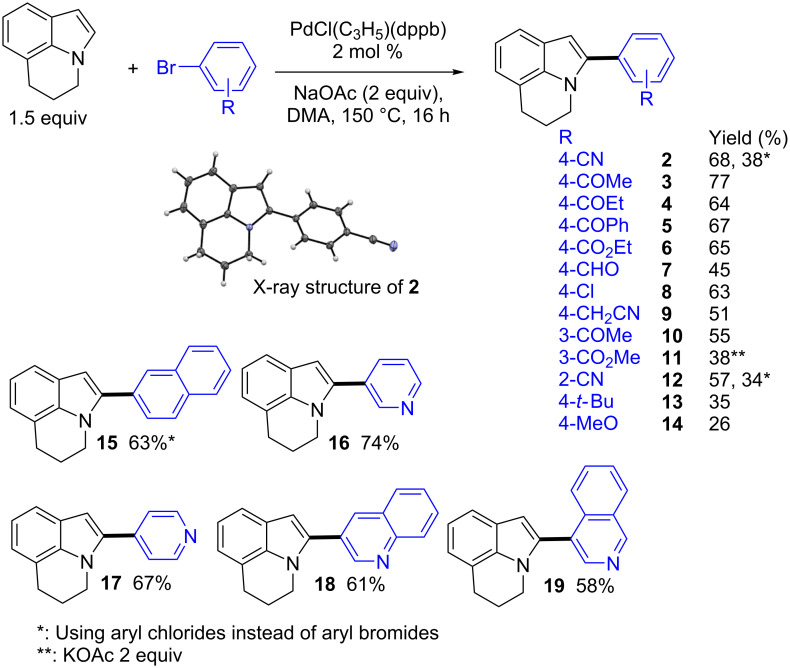
Synthesis of α-arylated lilolidine derivatives.

Then, a set of aryl bromides was reacted with lilolidine using 2 mol % of PdCl(C_3_H_5_)(dppb) catalyst, NaOAc or KOAc as bases in DMA at 150 °C (Scheme 2). We initially studied the reactivity of electron-deficient aryl bromides. Acetyl, propionyl, benzoyl and ester as *para*-substituents on the aryl bromides were tolerated affording the target products **2**–**6** in 64–77% yields. The structure of **2** was confirmed by X-ray analysis. A lower yield of **7** was obtained for the reaction of 4-bromobenzaldehyde with lilolidine due to the formation of degradation products. Good yields in **8** and **9** were obtained from 4-chloro- and 4-carbonitrile-substituted aryl bromides. In all cases, with these *para*-substituted aryl bromides, high regioselectivities in favor of the α-arylations were observed. The *meta*-substituted 3-bromoacetophenone and methyl 3-bromobenzoate also afforded the α-arylated lilolidines **10** and **11** with high regioselectivities. Conversely, with the more sterically hindered aryl source 2-bromobenzonitrile, a mixture of α- and β-arylated lilolidine derivatives was obtained (ratio α:β 69:31). The reactivity of two electron-rich aryl bromides was also examined. With 4-*tert*-butylbromobenzene and 4-bromoanisole, the target products **13** and **14** were obtained with high regioselectivities, but in low yields due to a partial conversion of these aryl bromides. It should be mentioned that the use of aryl chlorides instead of aryl bromides did not allow to improve the regioselectivities or the reaction yields. With 4-chlorobenzonitrile, product **2** was obtained in 80% regioselectivity and in 38% yield; whereas the use of 2-chlorobenzonitrile afforded **12** in 62% regioselectivity and in 34% yield. In both cases, partial conversions of the aryl chlorides were observed.

Pyridines and quinoline heterocycles are very important structures in pharmaceutical chemistry as more than 100 currently marketed drugs contain these units. Therefore, the reactivity of 3- and 4-bromo-substituted pyridines, 3-bromoquinoline and 4-bromoisoquinoline for the α-arylation of lilolidine was also studied. In all cases, the desired *N*-containing coupling products **16**–**19** were obtained in high regioselectivities and in 58–74% yields.

**Scheme 3:**
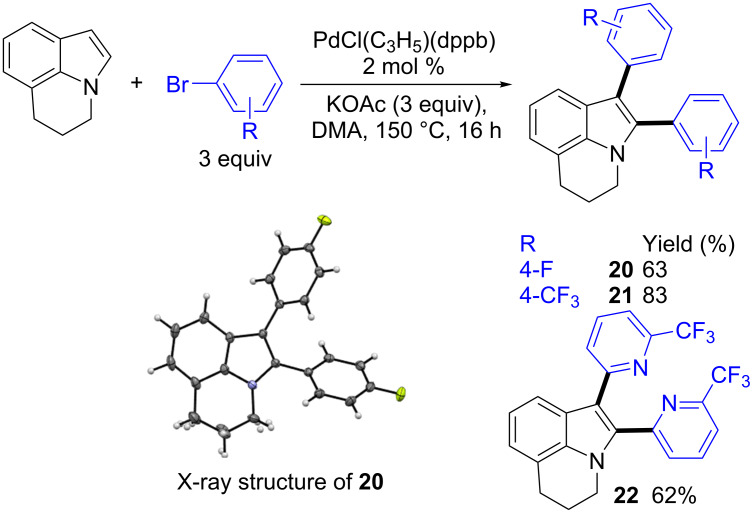
Synthesis of α,β-di(hetero)arylated lilolidine derivatives.

Then, the one-pot synthesis of α,β-di(hetero)arylated 5,6-dihydropyrrolo[3,2,1-*ij*]quinolines was attempted (Scheme 3). The use of a larger amount of aryl bromides (3 equiv) provided the target diarylated lilolidines **20**–**22** in good yields. Under these conditions, the mono-arylated lilolidines were detected in very low yields by GC–MS analysis of the crude mixtures. The structure of **20** was confirmed by X-ray diffraction.

**Scheme 4:**
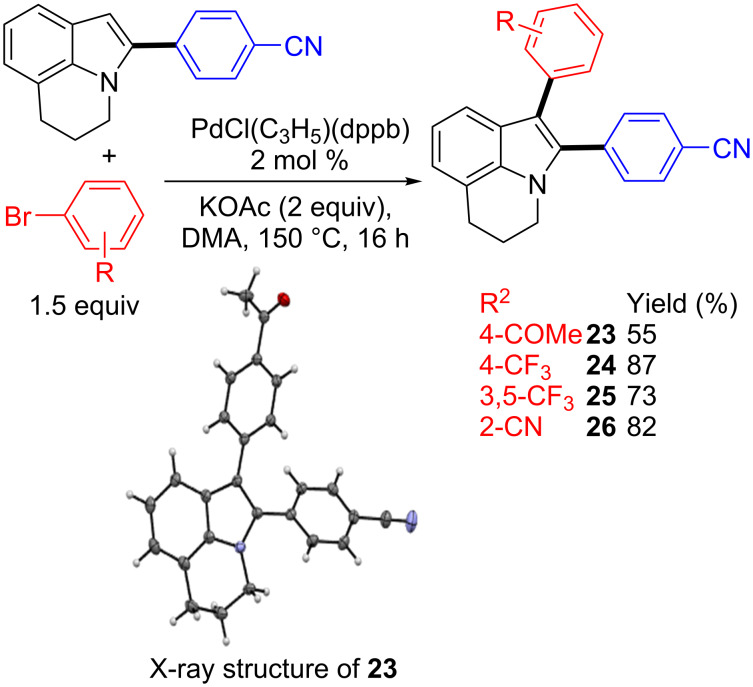
Synthesis of α,β-diarylated lilolidine derivatives via successive direct arylations.

As α-arylated lilolidines can be easily obtained under the reaction conditions shown in Scheme 2, the synthesis of α,β-diarylated 5,6-dihydropyrrolo[3,2,1-*ij*]quinolines containing two different aryl groups at α- and β-positions via sequential Pd-catalyzed C–H bond functionalization steps was studied (Scheme 4). The reaction of 1 equiv of 4-(5,6-dihydropyrrolo[3,2,1-*ij*]quinolin-2-yl)benzonitrile **2** and 1.5 equiv of a set of aryl bromides using again 2 mol % PdCl(C_3_H_5_)(dppb) catalyst associated to KOAc provided the desired diarylated lilolidines **23**–**26** in 55–87% yields. The structure of **23** was confirmed by X-ray diffraction.

**Scheme 5:**
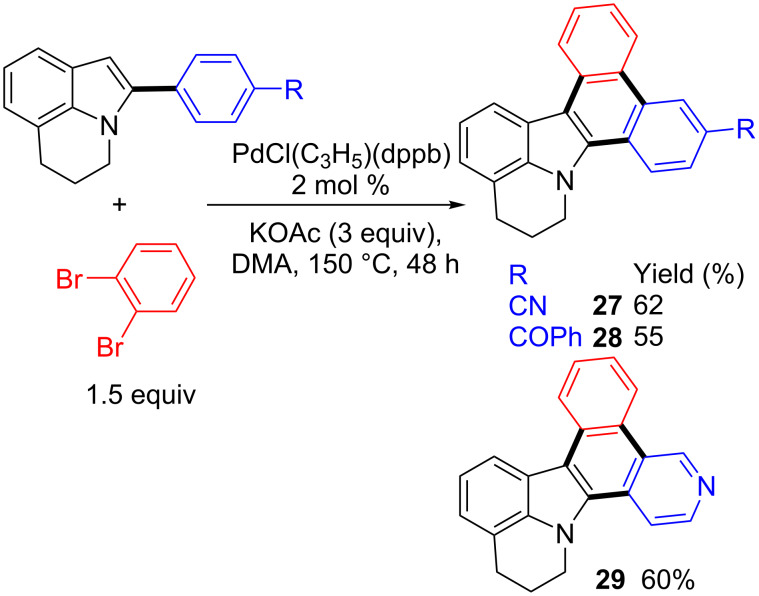
Synthesis of 5,6-dihydrodibenzo[*a*,*c*]pyrido[3,2,1-*jk*]carbazoles via successive direct arylations.

Finally, the synthesis of 5,6-dihydrodibenzo[*a*,*c*]pyrido[3,2,1-*jk*]carbazoles via β-arylation of the previously obtained α-arylated lilolidines followed by an intramolecular Pd-catalyzed direct arylation was examined (Scheme 5). The reaction of compound **2** with 1,2-dibromobenzene in the presence of 2 mol % PdCl(C_3_H_5_)(dppb) catalyst and KOAc as base afforded the desired carbazole **27** in moderate yield after 16 h due to a partial conversion of **2**. However, the use of a longer reaction time (48 h) allowed to reach an almost complete conversion of **2**, and the carbazole **27** was isolated in 62% yield. A slightly lower yield in the carbazole **28** was obtained from (4-(5,6-dihydropyrrolo[3,2,1-*ij*]quinolin-2-yl)phenyl)(phenyl)methanone **5** and 1,2-dibromobenzene. This sequential C–H bond arylation strategy was also effective for the synthesis of the carbazole **29** from the pyridine α-substituted lilolidine **17**.

## Conclusion

The late stage Pd-catalyzed C–H bond functionalization allows to prepare (di)arylated lilolidine derivatives in only one or two steps. The α-arylated lilolidines were generally obtained in high regioselectivities and in good yields using aryl bromides as easily available aryl sources, acetates as inexpensive bases and PdCl(C_3_H_5_)(dppb) as air-stable catalyst. The reaction tolerated a wide variety of useful functional groups such as nitrile, formyl, acetyl, propionyl, benzoyl, esters, chloro, or acetonitrile on the aryl bromide and the heteroaryl bromides 3- or 4-bromopyridines and 3-bromoquinoline. From these α-arylated lilolidines, a second Pd-catalyzed direct arylation at β-position gave rise to α,β-diarylated lilolidines with two different aryl units. The one pot access to α,β-diarylated lilolidines with two identical aryl groups was also possible by using a larger amount of aryl bromide. The synthesis of 5,6-dihydrodibenzo[*a*,*c*]pyrido[3,2,1-*jk*]carbazoles from lilolidine via three successive direct C–H bond arylations also proceed nicely. Therefore, this methodology provides a straightforward access to a wide variety of α- and β-(hetero)aryl-substituted lilolidines allowing to tune or modify their biological properties.

## Supporting Information

Supporting information features experimental procedures, products characterizations, copies of ^1^H and ^13^C NMR spectra of all products and CCDC numbers of products **2**, **20** and **23**.

File 1 Experimental procedures and NMR spectra of compounds **1**–**29**.
