# Supplementary material for: Regioselective Pd-catalyzed direct C1- and C2-arylations of lilolidine for the access to 5,6-dihydropyrrolo[3,2,1-ij]quinoline derivatives
Source: Beilstein J Org Chem. 2019 Aug 29;15:2069–75. doi: 10.3762/bjoc.15.204 (PMC6719733; doi:10.3762/bjoc.15.204)
Supplement: File 1 — Experimental procedures and NMR spectra of compounds 1–29. [file Beilstein_J_Org_Chem-15-2069-s001.pdf]

BEILSTEIN JOURNAL OF ORGANIC CHEMISTRY

Supporting Information

for

Regioselective Pd-catalyzed direct C1- and C2-arylations of lilolidine for the access to 5,6-dihydropyrrolo[3,2,1-*ij*]quinoline derivatives

Hai-Yun Huang, Haoran Li, Thierry Roisnel, Jean-François Soulé and Henri Doucet

Beilstein J. Org. Chem. **2019,** *15,* 2069–2075. doi:10.3762/bjoc.15.204

Experimental procedures and NMR spectra of compounds 1–29

License and Terms: This is a supporting information file under the terms of the Creative Commons Attribution License (http://creativecommons.org/licenses/by/4.0). Please note that the reuse, redistribution and reproduction in particular requires that the authors and source are credited.
The license is subject to the *Beilstein Journal of Organic Chemistry* terms and conditions: (https://www.beilstein-journals.org/bjoc)

Preparation and characterization of products 1a and 2–29 S2–S15
^{1}H and ^{13}C NMR spectra of all products S16–S48
CCDC numbers of products 2, 20 and 23 S49

General. All reactions were performed in Schlenk tubes under argon. DMA analytical grade were not distilled before use. Sodium acetate or potassium acetate 99+ were used. Commercial lilolidine (>98%) and aryl bromides were used without purification. ^{1}H (400 MHz), ^{13}C (100 MHz) spectra were recorded in $CDCl_3$ solutions. Chemical shifts are reported in ppm relative to $CDCl_3$ (^{1}H: 7.26 and ^{13}C: 77.16). Flash chromatography was performed on silica gel (230-400 mesh).

Preparation of the $PdCl(C_3H_5)(dppb)$ catalyst [1]: An oven-dried 40 mL Schlenk tube equipped with a magnetic stirring bar under argon atmosphere, was charged with $[Pd(C_3H_5)Cl]_2$ (182 mg, 0.5 mmol) and dppb (426 mg, 1 mmol). 10 mL of anhydrous dichloromethane were added, then, the solution was stirred at room temperature for twenty minutes. The solvent was removed under reduced pressure. The yellow powder was used without purification. ^{31}P NMR (81 MHz, $CDCl_3$) δ = 19.3 (s).

General procedure for the synthesis of the α-arylated lilolidine derivatives 1a and 2-22: As a typical experiment, the reaction of the aryl bromide (1 mmol), lilolidine (0.236 g, 1.5 mmol), NaOAc (0.164 g, 2 mmol) or KOAc (0.196 g, 2 mmol) (see schemes) at 150 °C during 16 h in DMA (2 mL) in the presence of $PdCl(C_3H_5)(dppb)$ (12.2 mg, 0.02 mmol) under argon afford the corresponding arylation product after evaporation of the solvent and purification on silica gel.

3-(5,6-Dihydropyrrolo[3,2,1-*ij*]quinolin-2-yl)benzonitrile (1a): From 3-bromobenzonitrile (0.182 g, 1 mmol) and lilolidine (0.236 g, 1.5 mmol), **1a** was obtained in 83% (0.214 g) yield as a white solid: mp 176-178 °C.

^{1}H NMR (400 MHz, $CDCl_3$): δ 7.83 (s, 1H), 7.79 (d, J = 8.0 Hz, 1H), 7.65 (d, J = 8.0 Hz, 1H), 7.57 (t, J = 7.9 Hz, 1H), 7.47 (d, J = 7.9 Hz, 1H), 7.07 (t, J = 7.2 Hz, 1H), 6.98 (d, J = 7.1 Hz, 1H), 6.61 (s, 1H), 4.21 (t, J = 5.7 Hz, 2H), 3.04 (t, J = 5.1 Hz, 2H), 2.24 (quint., J = 5.7 Hz, 2H).

^{13}C NMR (100 MHz, $CDCl_3$): δ 137.3, 135.8, 134.3, 132.8, 131.8, 130.9, 129.7, 125.9, 122.4, 120.5, 119.7, 118.7, 118.3, 113.1, 102.1, 44.0, 25.0, 23.3.

Elemental analysis: calcd (%) for $C_{18}H_{14}N_2$ (258.32): C 83.69, H 5.46; found: C 83.45, H 5.32.

4-(5,6-Dihydropyrrolo[3,2,1-*ij*]quinolin-2-yl)benzonitrile (2): From 4-bromobenzonitrile (0.182 g, 1 mmol) and lilolidine (0.236 g, 1.5 mmol), **2** was obtained in 68% (0.175 g) yield as a yellow solid: mp 201-203 °C.

^{1}H NMR (400 MHz, $CDCl_3$): δ 7.74 (d, *J* = 8.5 Hz, 2H), 7.66 (d, *J* = 8.5 Hz, 2H), 7.47 (d, *J* = 7.9 Hz, 1H), 7.05 (t, *J* = 7.9 Hz, 1H), 6.99 (d, *J* = 7.9 Hz, 1H), 6.66 (s, 1H), 4.24 (t, *J* = 5.7 Hz, 2H), 3.04 (t, *J* = 5.1 Hz, 2H), 2.23 (quint., *J* = 5.7 Hz, 2H).

^{13}C NMR (100 MHz, $CDCl_3$): δ 137.8, 137.4, 136.1, 132.5, 128.8, 125.9, 122.4, 120.6, 119.9, 119.0, 118.4, 110.9, 102.8, 44.3, 25.0, 23.3.

Elemental analysis: calcd (%) for $C_{18}H_{14}N_2$ (258.32): C 83.69, H 5.46; found: C 83.78, H 5.30.

1-(4-(5,6-Dihydropyrrolo[3,2,1-*ij*]quinolin-2-yl)phenyl)ethan-1-one (3)

From 4-bromoacetophenone (0.199 g, 1 mmol) and lilolidine (0.236 g, 1.5 mmol), **3** was obtained in 77% (0.212 g) yield as a yellow solid: mp 139-141 °C.

^{1}H NMR (400 MHz, $CDCl_3$): δ 8.05 (d, *J* = 8.5 Hz, 2H), 7.66 (d, *J* = 8.5 Hz, 2H), 7.48 (d, *J* = 7.9 Hz, 1H), 7.07 (t, *J* = 7.9 Hz, 1H), 6.99 (d, *J* = 7.9 Hz, 1H), 6.67 (s, 1H), 4.26 (t, *J* = 5.7 Hz, 2H), 3.04 (t, *J* = 5.1 Hz, 2H), 2.65 (s, 3H), 2.23 (quint., *J* = 5.7 Hz, 2H).

^{13}C NMR (100 MHz, $CDCl_3$): δ 197.6, 138.7, 137.5, 136.0, 135.9, 128.8, 128.5, 126.0, 122.4, 120.4, 119.5, 118.3, 102.2, 44.2, 26.8, 25.1, 23.3.

Elemental analysis: calcd (%) for $C_{19}H_{17}NO$ (275.35): C 82.88, H 6.22; found: C 83.02, H 6.30.

1-(4-(5,6-Dihydropyrrolo[3,2,1-*ij*]quinolin-2-yl)phenyl)propan-1-one (4)

From 4-bromopropiophenone (0.213 g, 1 mmol) and lilolidine (0.236 g, 1.5 mmol), **4** was obtained in 64% (0.184 g) yield as a white solid: mp 147-149 °C.

^{1}H NMR (400 MHz, $CDCl_3$): δ 8.06 (d, *J* = 8.5 Hz, 2H), 7.66 (d, *J* = 8.5 Hz, 2H), 7.48 (d, *J* = 7.9 Hz, 1H), 7.07 (t, *J* = 7.9 Hz, 1H), 6.98 (d, *J* = 7.9 Hz, 1H), 6.66 (s, 1H), 4.26 (t, *J* = 5.7 Hz, 2H), 3.11-3.00 (m, 4H), 2.23 (quint., *J* = 5.7 Hz, 2H), 1.27 (t, *J* = 7.5 Hz, 3H).

^{13}C NMR (100 MHz, $CDCl_3$): δ 200.4, 138.8, 137.3, 135.9, 135.7, 128.5, 128.4, 126.0, 122.4, 120.3, 119.5, 118.3, 102.1, 44.2, 32.0, 25.1, 23.3, 8.4.

Elemental analysis: calcd (%) for $C_{20}H_{19}NO$ (289.38): C 83.01, H 6.62; found: C 82.89, H 6.67.

Other regioisomer:

^{1}H NMR (400 MHz, $CDCl_3$): δ 8.03 (d, *J* = 8.5 Hz, 2H), 7.82-7.74 (m, 3H), 7.41 (s, 1H), 7.14 (t, *J* = 7.9 Hz, 1H), 6.99 (d, *J* = 7.9 Hz, 1H), 4.23 (t, *J* = 5.7 Hz, 2H), 3.10-2.99 (m, 4H), 2.29 (quint., *J* = 5.7 Hz, 2H), 1.26 (t, *J* = 7.5 Hz, 3H).

^{13}C NMR (100 MHz, $CDCl_3$): δ 200.4, 141.3, 135.3, 133.8, 128.9, 126.3, 125.0, 123.7, 122.5, 121.1, 119.6, 117.7, 115.6, 44.5, 31.7, 24.8, 22.9, 8.6.

(4-(5,6-Dihydropyrrolo[3,2,1-*ij*]quinolin-2-yl)phenyl)(phenyl)methanone (5)

From 4-bromobenzophenone (0.261 g, 1 mmol) and lilolidine (0.236 g, 1.5 mmol), **5** was obtained in 67% (0.226 g) yield as a yellow solid: mp 159-161 °C.

^{1}H NMR (400 MHz, $CDCl_3$): δ 7.93 (d, *J* = 8.5 Hz, 2H), 7.88 (d, *J* = 8.5 Hz, 2H), 7.70 (d, *J* = 8.3 Hz, 2H), 7.64 (t, *J* = 7.4 Hz, 1H), 7.57-7.47 (m, 3H), 7.09 (t, *J* = 7.7 Hz, 1H), 7.00 (d, *J* = 7.0 Hz, 1H), 6.70 (s, 1H), 4.29 (t, *J* = 5.7 Hz, 2H), 3.06 (t, *J* = 5.1 Hz, 2H), 2.25 (quint., *J* = 5.7 Hz, 2H).

^{13}C NMR (100 MHz, $CDCl_3$): δ 196.2, 138.7, 137.7, 136.9, 136.3, 135.9, 132.5, 130.6, 130.1, 128.4, 128.2, 126.0, 122.3, 120.3, 119.4, 118.2, 102.1, 44.2, 25.0, 23.3.

Elemental analysis: calcd (%) for $C_{24}H_{19}NO$ (337.42): C 85.43, H 5.68; found: C 85.28, H 5.29.

Ethyl 4-(5,6-Dihydropyrrolo[3,2,1-*ij*]quinolin-2-yl)benzoate (6)

From ethyl 4-bromobenzoate (0.229 g, 1 mmol) and lilolidine (0.236 g, 1.5 mmol), **6** was obtained in 65% (0.198 g) yield as a white solid: mp 109-111 °C.

^{1}H NMR (400 MHz, $CDCl_3$): δ 8.15 (d, *J* = 8.4 Hz, 2H), 7.64 (d, *J* = 8.4 Hz, 2H), 7.48 (d, *J* = 7.9 Hz, 1H), 7.07 (t, *J* = 7.9 Hz, 1H), 6.98 (d, *J* = 7.9 Hz, 1H), 6.66 (s, 1H), 4.43 (q, *J* = 7.5 Hz, 2H), 4.25 (t, *J* = 5.7 Hz, 2H), 3.04 (t, *J* = 5.1 Hz, 2H), 2.23 (quint., *J* = 5.7 Hz, 2H), 1.44 (t, *J* = 7.5 Hz, 3H).

^{13}C NMR (100 MHz, $CDCl_3$): δ 166.5, 138.8, 137.2, 135.9, 130.0, 129.4, 128.3, 126.0, 122.3, 120.3, 119.4, 118.2, 102.0, 61.2, 44.2, 25.1, 23.3, 14.5.

Elemental analysis: calcd (%) for $C_{20}H_{19}NO_2$ (305.38): C 78.66, H 6.27; found: C 78.89, H 6.36.

4-(5,6-Dihydropyrrolo[3,2,1-*ij*]quinolin-2-yl)benzaldehyde (7)

From 4-bromobenzaldehyde (0.185 g, 1 mmol) and lilolidine (0.236 g, 1.5 mmol), **7** was obtained in 45% (0.117 g) yield as a yellow solid: mp 143-145 °C.

^{1}H NMR (400 MHz, $CDCl_3$): δ 10.06 (s, 1H), 7.96 (d, *J* = 8.5 Hz, 2H), 7.73 (d, *J* = 8.5 Hz, 2H), 7.49 (d, *J* = 7.9 Hz, 1H), 7.07 (t, *J* = 7.9 Hz, 1H), 6.99 (d, *J* = 7.9 Hz, 1H), 6.70 (s, 1H), 4.27 (t, *J* = 5.7 Hz, 2H), 3.05 (t, *J* = 5.1 Hz, 2H), 2.24 (quint., *J* = 5.7 Hz, 2H).

^{13}C NMR (100 MHz, $CDCl_3$): δ 191.8, 138.9, 138.4, 136.1, 135.2, 130.2, 128.8, 126.0, 122.4, 120.5, 119.7, 118.4, 102.7, 44.3, 25.1, 23.3.

Elemental analysis: calcd (%) for $C_{18}H_{15}NO$ (261.32): C 82.73, H 5.79; found: C 82.89, H 5.64.

2-(4-Chlorophenyl)-5,6-dihydropyrrolo[3,2,1-ij]quinoline (8)

From 4-bromochlorobenzene (0.191 g, 1 mmol) and lilolidine (0.236 g, 1.5 mmol), **8** was obtained in 63% (0.168 g) yield as a white solid: mp 147-149 °C.

^{1}H NMR (400 MHz, $CDCl_3$): δ 7.95 (d, *J* = 8.5 Hz, 2H), 7.44 (d, *J* = 7.9 Hz, 1H), 7.43 (d, *J* = 8.5 Hz, 2H), 7.04 (t, *J* = 7.9 Hz, 1H), 6.95 (d, *J* = 7.9 Hz, 1H), 6.54 (s, 1H), 4.19 (t, *J* = 5.7Hz, 2H), 3.03 (t, *J* = 5.1 Hz, 2H), 2.22 (quint., *J* = 5.7 Hz, 2H).

^{13}C NMR (100 MHz, $CDCl_3$): δ 138.8, 135.5, 133.8, 131.4, 129.9, 128.9, 126.0, 122.2, 120.2, 119.1, 118.1, 101.1, 43.9, 25.1, 23.3.

Elemental analysis: calcd (%) for $C_{17}H_{14}ClN$ (267.76): C 76.26, H 5.27; found: C 76.39, H 5.41.

2-(4-(5,6-Dihydropyrrolo[3,2,1-*ij*]quinolin-2-yl)phenyl)acetonitrile (9)

From 2-(4-bromophenyl)acetonitrile (0.196 g, 1 mmol) and lilolidine (0.236 g, 1.5 mmol), **9** was obtained in 51% (0.139 g) yield as a yellow solid: mp 130-132 °C.

^{1}H NMR (400 MHz, $CDCl_3$): δ 7.58 (d, *J* = 8.5 Hz, 2H), 7.48 (d, *J* = 7.9 Hz, 1H), 7.42 (d, *J* = 8.5 Hz, 2H), 7.07 (t, *J* = 7.9 Hz, 1H), 6.98 (d, *J* = 7.9 Hz, 1H), 6.58 (s, 1H), 4.21 (t, *J* = 5.7 Hz, 2H), 3.80 (s, 2H), 3.04 (t, *J* = 5.1 Hz, 2H), 2.23 (quint., *J* = 5.7 Hz, 2H).

^{13}C NMR (100 MHz, $CDCl_3$): δ 139.0, 135.5, 132.8, 129.3, 129.2, 128.3, 126.0, 122.2, 120.2, 119.1, 118.0, 117.8, 101.1, 43.9, 25.1, 23.5, 23.3.

Elemental analysis: calcd (%) for $C_{19}H_{16}N_2$ (272.35): C 83.79, H 5.92; found: C 83.58, H 5.69.

1-(3-(5,6-Dihydropyrrolo[3,2,1-*ij*]quinolin-2-yl)phenyl)ethan-1-one (10)

From 3-bromoacetophenone (0.199 g, 1 mmol) and lilolidine (0.236 g, 1.5 mmol), **10** was obtained in 55% (0.151 g) yield as a yellow solid: mp 159-161 °C.

^{1}H NMR (400 MHz, $CDCl_3$): δ 8.15 (s, 1H), 7.96 (d, *J* = 8.5 Hz, 1H), 7.76 (d, *J* = 7.8 Hz, 1H), 7.57 (t, *J* = 7.9 Hz, 1H), 7.47 (d, *J* = 7.8 Hz, 1H), 7.06 (t, *J* = 7.9 Hz, 1H), 6.97 (d, *J* = 7.9 Hz, 1H), 6.62 (s, 1H), 4.22 (t, *J* = 5.7 Hz, 2H), 3.04 (t, *J* = 5.1 Hz, 2H), 2.66 (s, 3H), 2.23 (quint., *J* = 5.7 Hz, 2H).

^{13}C NMR (100 MHz, $CDCl_3$): δ 197.9, 138.9, 137.6, 135.6, 133.5, 133.1, 129.1, 128.4, 127.5, 126.0, 122.3, 120.3, 119.2, 118.1, 101.4, 43.9, 26.9, 25.1, 23.3.

Elemental analysis: calcd (%) for $C_{19}H_{17}NO$ (275.35): C 82.88, H 6.22; found: C 82.78, H 6.08.

Methyl 3-(5,6-dihydropyrrolo[3,2,1-*ij*]quinolin-2-yl)benzoate (11)

From methyl 3-bromobenzoate (0.215 g, 1 mmol) and lilolidine (0.236 g, 1.5 mmol), **11** was obtained in 38% (0.110 g) yield as a yellow solid: mp 146-148 °C.

^{1}H NMR (400 MHz, $CDCl_3$): δ 8.25 (s, 1H), 8.05 (d, *J* = 8.5 Hz, 1H), 7.76 (d, *J* = 8.5 Hz, 1H), 7.54 (t, *J* = 7.8 Hz, 1H), 7.48 (d, *J* = 7.8 Hz, 1H), 7.06 (t, *J* = 7.9 Hz, 1H), 6.97 (d, *J* = 7.9 Hz, 1H), 6.62 (s, 1H), 4.24 (t, *J* = 5.7 Hz, 2H), 3.97 (s, 3H), 3.04 (t, *J* = 5.1 Hz, 2H), 2.23 (quint., *J* = 5.7 Hz, 2H).

^{13}C NMR (100 MHz, $CDCl_3$): δ 167.0, 138.8, 135.6, 133.2, 133.0, 130.7, 129.6, 128.9, 128.7, 126.0, 122.3, 120.3, 119.1, 118.1, 101.3, 52.4, 43.9, 25.1, 23.3.

Elemental analysis: calcd (%) for $C_{19}H_{17}NO_2$ (291.35): C 78.33, H 5.88; found: C 78.60, H 6.04.

2-(5,6-Dihydropyrrolo[3,2,1-*ij*]quinolin-2-yl)benzonitrile (12): From 2-bromobenzonitrile (0.182 g, 1 mmol) and lilolidine (0.236 g, 1.5 mmol), **12** was obtained in 57% (0.147 g) yield as a white solid: mp 225-227 °C.

^{1}H NMR (400 MHz, $CDCl_3$): δ 7.81 (dd, *J* = 7.8, 0.8 Hz, 1H), 7.67 (td, *J* = 7.8, 1.3 Hz, 1H), 7.56 (d, *J* = 7.9 Hz, 1H), 7.52-7.44 (m, 2H), 7.07 (t, *J* = 7.9 Hz, 1H), 6.99 (d, *J* = 7.9 Hz, 1H), 6.73 (s, 1H), 4.11 (t, *J* = 5.7 Hz, 2H), 3.05 (t, *J* = 5.1 Hz, 2H), 2.25 (quint., *J* = 5.7 Hz, 2H).

^{13}C NMR (100 MHz, $CDCl_3$): δ 136.4, 135.7, 135.2, 133.9, 132.6, 131.0, 128.2, 125.9, 122.4, 120.4, 119.8, 118.6, 118.5, 112.9, 103.9, 43.6, 25.0, 23.1.

Elemental analysis: calcd (%) for $C_{18}H_{14}N_2$ (258.32): C 83.69, H 5.46; found: C 83.76, H 5.51.

The regioisomers 2-(5,6-Dihydropyrrolo[3,2,1-*ij*]quinolin-1-yl)benzonitrile was also isolated in 19% yield (31% selectivity): ^{1}H NMR (400 MHz, $CDCl_3$): δ 7.83 (d, *J* = 7.9 Hz, 1H), 7.75 (dd, *J* = 7.8, 1.0 Hz, 1H), 7.66 (s, 1H), 7.63 (td, *J* = 7.7, 1.2 Hz, 1H), 7.60 (d, *J* = 7.9 Hz, 1H), 7.31 (td, *J* = 7.9, 1.1 Hz, 1H), 7.12 (t, *J* = 7.9 Hz, 1H), 7.01 (d, *J* = 7.5 Hz, 1H), 4.26 (t, *J* = 5.7 Hz, 2H), 3.05 (t, *J* = 5.1 Hz, 2H), 2.30 (quint., *J* = 5.7 Hz, 2H).

^{13}C NMR (100 MHz, $CDCl_3$): δ 139.7, 134.7, 134.2, 132.9, 129.6, 126.4, 125.7, 124.2, 122.5, 121.1, 120.0, 119.6, 117.1, 112.4, 110.1, 44.6, 24.8, 22.9.

2-(4-(*tert*-Butyl)phenyl)-5,6-dihydropyrrolo[3,2,1-*ij*]quinoline (13)

From 4-*tert*-butylbromobenzene (0.213 g, 1 mmol) and lilolidine (0.236 g, 1.5 mmol), **13** was obtained in 35% (0.101 g) yield as a yellow oil.

^{1}H NMR (400 MHz, $CDCl_3$): δ 7.52-7.49 (m, 4H), 7.44 (d, *J* = 7.9 Hz, 1H), 7.04 (t, *J* = 7.9 Hz, 1H), 6.93 (d, *J* = 7.9 Hz, 1H), 6.57 (s, 1H), 4.27 (t, *J* = 5.7Hz, 2H), 3.06 (t, *J* = 5.1 Hz, 2H), 2.24 (quint., *J* = 5.7 Hz, 2H), 1.41 (s, 9H).

^{13}C NMR (100 MHz, $CDCl_3$): δ 150.8, 140.1, 135.4, 130.0, 128.5, 126.2, 125.6, 122.1, 119.9, 118.6, 117.8, 100.3, 43.9, 34.8, 31.5, 25.2, 23.3.

Elemental analysis: calcd (%) for $C_{21}H_{23}N$ (289.42): C 87.15, H 8.01; found: C 87.39, H 7.78.

2-(4-Methoxyphenyl)-5,6-dihydropyrrolo[3,2,1-*ij*]quinoline (14)

From 4-bromoanisole (0.187 g, 1 mmol) and lilolidine (0.236 g, 1.5 mmol), **14** was obtained in 26% (0.068 g) yield as a yellow oil.

^{1}H NMR (400 MHz, $CDCl_3$): δ 7.49 (d, *J* = 8.0 Hz, 2H), 7.44 (d, *J* = 7.9 Hz, 1H), 7.03 (t, *J* = 7.9 Hz, 1H), 6.99 (d, *J* = 8.0 Hz, 2H), 6.92 (d, *J* = 7.9 Hz, 1H), 6.48 (s, 1H), 4.19 (t, *J* = 5.7Hz, 2H), 3.87 (s, 3H), 3.02 (t, *J* = 5.1 Hz, 2H), 2.20 (quint., *J* = 5.7 Hz, 2H).

^{13}C NMR (100 MHz, $CDCl_3$): δ 159.4, 140.0, 135.2, 130.0, 126.2, 125.4, 122.1, 119.9, 118.5, 117.7, 114.2, 99.9, 55.5, 43.8, 25.1, 23.3.

Elemental analysis: calcd (%) for $C_{18}H_{17}NO$ (263.34): C 82.10, H 6.51; found: C 82.39, H 6.54.

2-(Naphthalen-2-yl)-5,6-dihydropyrrolo[3,2,1-*ij*]quinoline (15)

From 2-bromonaphthalene (0.207 g, 1 mmol) and lilolidine (0.236 g, 1.5 mmol), **15** was obtained in 63% (0.178 g) yield as a yellow solid: mp 147-149 °C.

^{1}H NMR (400 MHz, $CDCl_3$): δ 8.00 (bs, 1H), 7.93 (d, *J* = 8.5 Hz, 1H), 7.91-7.87 (m, 2H), 7.70 (dd, *J* = 8.4, 1.7 Hz, 1H), 7.57-7.47 (m, 3H), 7.06 (t, *J* = 7.4 Hz, 1H), 6.69 (dd, *J* = 7.0, 1.0 Hz, 1H), 6.67 (s, 1H), 4.31 (t, *J* = 5.7 Hz, 2H), 3.06 (t, *J* = 5.1 Hz, 2H), 2.25 (quint., *J* = 5.7 Hz, 2H).

^{13}C NMR (100 MHz, $CDCl_3$): δ 140.1, 135.6, 133.5, 132.8, 130.3, 128.3, 128.2, 127.9, 127.4, 126.9, 126.6, 126.4, 126.2, 122.2, 120.1, 118.9, 118.0, 101.2, 44.1, 25.2, 23.4.

Elemental analysis: calcd (%) for $C_{21}H_{17}N$ (283.37): C 89.01, H 6.05; found: C 88.78, H 6.20.

2-(Pyridin-3-yl)-5,6-dihydropyrrolo[3,2,1-*ij*]quinoline (16)

From 3-bromopyridine (0.158 g, 1 mmol) and lilolidine (0.236 g, 1.5 mmol), **16** was obtained in 74% (0.173 g) yield as a yellow solid: mp 99-101 °C.

^{1}H NMR (400 MHz, $CDCl_3$): δ 8.84 (d, *J* = 1.3 Hz, 1H), 8.62 (dd, *J* = 4.6, 1.2 Hz, 1H), 7.86 (dt, J = 7.9, 2.0 Hz, 1H), 7.48 (d, *J* = 7.9 Hz, 1H), 7.40 (dd, J = 7.8, 4.8 Hz, 1H), 7.07 (t, *J* = 7.9 Hz, 1H), 6.98 (d, *J* = 7.9 Hz, 1H), 6.62 (s, 1H), 4.22 (t, *J* = 5.7 Hz, 2H), 3.04 (t, *J* = 5.1 Hz, 2H), 2.24 (quint., *J* = 5.7 Hz, 2H).

^{13}C NMR (100 MHz, $CDCl_3$): δ 149.4, 148.7, 136.2, 135.7, 128.9, 126.0, 123.5, 122.3, 120.4, 119.4, 118.2, 101.8, 43.9, 25.0, 23.2.

Elemental analysis: calcd (%) for $C_{16}H_{14}N_2$ (234.30): C 82.02, H 6.02; found: C 81.78, H 5.85.

Other regioisomer:

^{1}H NMR (400 MHz, $CDCl_3$): δ 8.95 (s, 1H), 8.47 (d, *J* = 3.9 Hz, 1H), 7.98 (d, *J* = 7.9 Hz, 1H), 7.72 (d, *J* = 8.1 Hz, 1H), 7.37-7.32 (m, 2H), 7.11 (t, *J* = 7.9 Hz, 1H), 6.99 (d, *J* = 7.9 Hz, 1H), 4.24 (t, *J* = 5.7 Hz, 2H), 3.04 (t, *J* = 5.1 Hz, 2H), 2.29 (quint., *J* = 5.7 Hz, 2H).

^{13}C NMR (100 MHz, $CDCl_3$): δ 147.9, 146.4, 135.2, 133.9, 124.2, 123.8, 123.7, 122.5, 121.0, 119.6, 117.2, 112.9, 44.5, 24.8, 22.9.

2-(Pyridin-4-yl)-5,6-dihydropyrrolo[3,2,1-*ij*]quinoline (17)

From 4-bromopyridine (0.158 g, 1 mmol) and lilolidine (0.236 g, 1.5 mmol), **17** was obtained in 67% (0.157 g) yield as a yellow solid: mp 111-113 °C.

^{1}H NMR (400 MHz, $CDCl_3$): δ 8.67 (d, *J* = 6.0 Hz, 2H), 7.51-7.44 (m, 3H), 7.07 (t, *J* = 7.9 Hz, 1H), 6.99 (d, *J* = 7.9 Hz, 1H), 6.73 (s, 1H), 4.28 (t, *J* = 5.7 Hz, 2H), 3.04 (t, *J* = 5.1 Hz, 2H), 2.24 (quint., *J* = 5.7 Hz, 2H).

^{13}C NMR (100 MHz, $CDCl_3$): δ 150.2, 140.3, 136.8, 136.2, 125.8, 122.6, 122.5, 120.5, 119.9, 118.5, 102.8, 44.3, 25.0, 23.3.

Elemental analysis: calcd (%) for $C_{16}H_{14}N_2$ (234.30): C 82.02, H 6.02; found: C 81.85, H 6.14.

2-(Quinolin-3-yl)-5,6-dihydropyrrolo[3,2,1-*ij*]quinoline (18)

From 3-bromoquinoline (0.208 g, 1 mmol) and lilolidine (0.236 g, 1.5 mmol), **18** was obtained in 61% (0.173 g) yield as a yellow solid: mp 151-153 °C.

^{1}H NMR (400 MHz, $CDCl_3$): δ 9.17 (d, J = 2.0 Hz, 1H), 8.27 (d, J = 2.0 Hz, 1H), 8.17 (d, *J* = 8.4 Hz, 1H), 7.89 (d, *J* = 8.4 Hz, 1H), 7.76 (t, *J* = 7.5 Hz, 1H), 7.61 (t, *J* = 7.5 Hz, 1H), 7.52 (d, *J* = 7.9 Hz, 1H), 7.10 (t, *J* = 7.9 Hz, 1H), 7.00 (d, *J* = 7.9 Hz, 1H), 6.75 (s, 1H), 4.31 (t, *J* = 5.7 Hz, 2H), 3.07 (t, *J* = 5.1 Hz, 2H), 2.27 (quint., *J* = 5.7 Hz, 2H).

^{13}C NMR (100 MHz, $CDCl_3$): δ 150.8, 147.3, 136.8, 135.8, 134.4, 129.8, 129.5, 128.0, 127.8, 127.4, 126.1, 122.3, 120.4, 119.5, 118.3, 102.2, 44.0, 25.0, 23.3.

Elemental analysis: calcd (%) for $C_{20}H_{16}N_2$ (284.36): C 84.48, H 5.67; found: C 84.29, H 5.57.

2-(Isoquinolin-4-yl)-5,6-dihydropyrrolo[3,2,1-*ij*]quinoline (19)

From 4-bromoisoquinoline (0.208 g, 1 mmol) and lilolidine (0.236 g, 1.5 mmol), **19** was obtained in 58% (0.165 g) yield as a yellow solid: mp 155-157 °C.

^{1}H NMR (400 MHz, $CDCl_3$): δ 9.33 (s, 1H), 8.63 (s, 1H), 8.08 (d, *J* = 8.4 Hz, 1H), 7.89 (d, *J* = 8.2 Hz, 1H), 7.76-7.63 (m, 2H), 7.55 (d, *J* = 8.0 Hz, 1H), 7.13 (t, *J* = 7.6 Hz, 1H), 7.03 (d, *J* =

7.9 Hz, 1H), 6.68 (s, 1H), 3.91 (t, J = 5.7 Hz, 2H), 3.07 (t, J = 5.1 Hz, 2H), 2.21 (quint., J = 5.7 Hz, 2H).

^{13}C NMR (100 MHz, $CDCl_3$): δ 152.9, 144.4, 135.4, 135.3, 134.2, 131.1, 128.4, 128.0, 127.6, 126.1, 125.1, 124.2, 122.1, 120.3, 119.2, 118.0, 103.6, 43.2, 25.0, 23.1.

Elemental analysis: calcd (%) for $C_{20}H_{16}N_2$ (284.36): C 84.48, H 5.67; found: C 84.39, H 5.71.

Other regioisomer:

^{1}H NMR (400 MHz, $CDCl_3$): δ 9.24 (s, 1H), 8.68 (s, 1H), 8.17 (d, J = 8.6 Hz, 1H), 8.04 (d, J = 8.4 Hz, 1H), 7.68-7.60 (m, 2H), 7.36 (d, J = 8.0 Hz, 1H), 7.32 (s, 1H), 7.08 (t, J = 7.6 Hz, 1H), 7.02 (d, J = 7.9 Hz, 1H), 4.28 (t, J = 5.7 Hz, 2H), 3.09 (t, J = 5.1 Hz, 2H), 2.24 (quint., J = 5.7 Hz, 2H).

^{13}C NMR (100 MHz, $CDCl_3$): δ 151.0, 143.4, 134.9, 134.7, 130.1, 128.7, 127.9, 127.2, 127.1, 125.9, 125.7, 125.5, 122.3, 120.5, 119.2, 117.8, 111.3, 44.4, 24.8, 23.0.

General procedure for the synthesis of the *α,β*-diarylated lilolidine derivatives 20–22:

As a typical experiment, the reaction of the aryl bromide (3 mmol), lilolidine (0.157 g, 1 mmol), KOAc (0.294 g, 3 mmol) at 150 °C during 16 h in DMA (5 mL) in the presence of $PdCl(C_3H_5)$(dppb) (12.2 mg, 0.02 mmol) under argon afford the corresponding diarylation product after evaporation of the solvent and purification on silica gel.

1,2-Bis(4-fluorophenyl)-5,6-dihydropyrrolo[3,2,1-ij]quinoline (20)

From 4-bromofluorobenzene (0.525 g, 3 mmol) and lilolidine (0.157 g, 1 mmol), **20** was obtained in 63% (0.217 g) yield as a white solid: mp 177-179 °C.

^{1}H NMR (400 MHz, $CDCl_3$): δ 7.54 (d, J = 7.9 Hz, 1H), 7.33-7.22 (m, 4H), 7.13-7.05 (m, 3H), 7.03-6.95 (m, 3H) 4.06 (t, J = 5.7 Hz, 2H), 3.06 (t, J = 5.1 Hz, 2H), 2.25 (quint., J = 5.7 Hz, 2H).

^{13}C NMR (100 MHz, $CDCl_3$): δ 162.6 (d, J = 248.3 Hz), 161.2 (d, J = 244.5 Hz), 135.0, 134.4, 132.5 (d, J = 8.1 Hz), 131.5 (d, J = 3.2 Hz), 131.2 (d, J = 7.7 Hz), 127.7 (d, J = 3.2 Hz), 125.0, 122.2, 120.6, 119.5, 116.9, 115.8 (d, J = 21.5 Hz), 115.4 (d, J = 21.2 Hz), 113.8, 43.3, 25.2, 23.1.

Elemental analysis: calcd (%) for $C_{23}H_{17}F_2N$ (345.39): C 79.98, H 4.96; found: C 80.21, H 4.89.

1,2-Bis(4-(trifluoromethyl)phenyl)-5,6-dihydropyrrolo[3,2,1-ij]quinoline (21)

From 4-bromobenzotrifluoride (0.676 g, 3 mmol) and lilolidine (0.157 g, 1 mmol), **21** was obtained in 83% (0.369 g) yield as a white solid: mp 220-222 °C.

^{1}H NMR (400 MHz, $CDCl_3$): δ 7.67 (d, *J* = 8.1 Hz, 2H), 7.59 (d, *J* = 7.9 Hz, 1H), 7.55 (d, *J* = 8.1 Hz, 2H), 7.46 (d, *J* = 8.1 Hz, 2H), 7.41 (d, *J* = 8.1 Hz, 2H), 7.15 (t, *J* = 7.9 Hz, 1H), 7.06 (d, *J* = 7.9 Hz, 1H), 4.09 (t, *J* = 5.7 Hz, 2H), 3.08 (t, *J* = 5.1 Hz, 2H), 2.26 (quint., *J* = 5.7 Hz, 2H).

^{13}C NMR (100 MHz, $CDCl_3$): δ 139.1, 135.2, 135.0, 134.8, 131.1, 130.3 (q, *J* = 32.4 Hz), 129.8, 127.7 (q, *J* = 32.4 Hz), 125.8 (q, *J* = 3.8 Hz), 125.5 (q, *J* = 3.8 Hz), 124.9, 124.5 (q, *J* = 271.7 Hz), 124.2 (q, *J* = 272.3 Hz), 122.5, 121.2, 120.2, 117.0, 114.3, 43.6, 25.1, 23.1.

Elemental analysis: calcd (%) for $C_{25}H_{17}F_6N$ (445.41): C 67.42, H 3.85; found: C 67.56, H 3.99.

1,2-Bis(6-(trifluoromethyl)pyridin-2-yl)-5,6-dihydropyrrolo[3,2,1-*ij*]quinoline (22)

From 2-bromo-6-trifluoromethylpyridine (0.678 g, 3 mmol) and lilolidine (0.157 g, 1 mmol), **22** was obtained in 62% (0.277 g) yield as a yellow solid: mp 143-145 °C.

^{1}H NMR (400 MHz, $CDCl_3$): δ 7.85-7.77 (m, 3H), 7.75 (t, *J* = 7.8 Hz, 1H), 7.65 (dd, *J* = 6.3, 2.5 Hz, 1H), 7.57 (d, *J* = 8.0 Hz, 1H), 7.46 (d, *J* = 7.7 Hz, 1H), 7.17 (t, *J* = 7.9 Hz, 1H), 7.06 (d, *J* = 7.9 Hz, 1H), 4.31 (t, *J* = 5.7 Hz, 2H), 3.05 (t, *J* = 5.1 Hz, 2H), 2.25 (quint., *J* = 5.7 Hz, 2H).

^{13}C NMR (100 MHz, $CDCl_3$): δ 155.5, 151.9, 148.0 (q, *J* = 34.6 Hz), 137.4, 135.2, 135.1, 129.8, 126.2, 124.6, 123.0, 121.8, 121.7 (q, *J* = 273.8 Hz), 121.6 (q, *J* = 273.0 Hz), 120.8, 119.1 (m), 118.0, 116.9 (m), 114.9, 44.1, 25.1, 23.1.

Elemental analysis: calcd (%) for $C_{23}H_{15}F_6N_3$ (447.38): C 61.75, H 3.38; found: C 61.79, H 3.50.

General procedure for the synthesis of the *α,β*-diarylated lilolidine derivatives 23–29: As a typical experiment, the reaction of the aryl bromide (1.5 mmol), 5,6-dihydropyrrolo[3,2,1-*ij*]quinolin-2-yl derivative **2**, **5** or **17** (1 mmol), KOAc (0.192 g, 2 mmol) at 150 ºC during 16 h in DMA (2 mL) in the presence of $PdCl(C_3H_5)$(dppb) (12.2 mg, 0.02 mmol) under argon afford the corresponding arylation product after evaporation of the solvent and purification on silica gel.

4-(1-(4-Acetylphenyl)-5,6-dihydropyrrolo[3,2,1-*ij*]quinolin-2-yl)benzonitrile (23)

From 4-bromoacetophenone (0.299 g, 1.5 mmol) and 4-(5,6-dihydropyrrolo[3,2,1-*ij*]quinolin-2-yl)benzonitrile (**2**, 0.258 g, 1 mmol), **23** was obtained in 55% (0.207 g) yield as a yellow solid: mp 243-245 °C.

^{1}H NMR (400 MHz, $CDCl_3$): δ 7.90 (d, *J* = 8.5 Hz, 2H), 7.68 (d, *J* = 8.5 Hz, 2H), 7.60 (d, *J* = 7.9 Hz, 1H), 7.45 (d, *J* = 8.5 Hz, 2H), 7.37 (d, *J* = 8.5 Hz, 2H), 7.15 (t, *J* = 7.9 Hz, 1H), 7.07 (d, *J* = 7.9 Hz, 1H), 4.10 (t, *J* = 5.7 Hz, 2H), 3.08 (t, *J* = 5.1 Hz, 2H), 2.60 (s, 3H), 2.27 (quint., *J* = 5.7 Hz, 2H).

^{13}C NMR (100 MHz, $CDCl_3$): δ 197.7, 140.3, 136.4, 135.0, 134.7, 134.5, 132.5, 131.4, 129.7, 128.8, 124.8, 122.6, 121.4, 120.5, 118.6, 117.2, 115.2, 111.9, 43.7, 26.7, 25.1, 23.0.

Elemental analysis: calcd (%) for $C_{26}H_{20}N_2O$ (376.46): C 82.95, H 5.36; found: C 82.99, H 5.58.

4-(1-(4-(Trifluoromethyl)phenyl)-5,6-dihydropyrrolo[3,2,1-*ij*]quinolin-2-yl)benzonitrile (24)

From 1-bromo-4-trifluoromethylbenzene (0.338 g, 1.5 mmol) and 4-(5,6-dihydropyrrolo[3,2,1-*ij*]quinolin-2-yl)benzonitrile (**2**, 0.258 g, 1 mmol), **24** was obtained in 87% (0.350 g) yield as a white solid: mp 239-241 °C.

^{1}H NMR (400 MHz, $CDCl_3$): δ 7.72 (d, *J* = 8.5 Hz, 2H), 7.61 (d, *J* = 7.9 Hz, 1H), 7.59 (d, *J* = 8.5 Hz, 2H), 7.48 (d, *J* = 8.5 Hz, 2H), 7.41 (d, *J* = 8.5 Hz, 2H), 7.18 (t, *J* = 7.9 Hz, 1H), 7.10 (d, *J* = 7.9 Hz, 1H), 4.14 (t, *J* = 5.7 Hz, 2H), 3.12 (t, *J* = 5.1 Hz, 2H), 2.30 (quint., *J* = 5.7 Hz, 2H).

^{13}C NMR (100 MHz, $CDCl_3$): δ 138.9, 136.3, 134.9, 134.4, 132.5, 131.3, 129.9, 128.0 (q, *J* = 32.4 Hz), 125.6 (q, *J* = 3.7 Hz), 124.8, 124.6 (q, *J* = 271.8 Hz), 122.6, 121.3, 120.5, 118.6, 117.0, 114.9, 111.9, 43.6, 25.1, 23.0.

Elemental analysis: calcd (%) for $C_{25}H_{17}F_3N_2$ (402.42): C 74.62, H 4.26; found: C 74.39, H 4.36.

4-(1-(3,5-Bis(trifluoromethyl)phenyl)-5,6-dihydropyrrolo[3,2,1-*ij*]quinolin-2-yl)benzonitrile (25)

From 1,3-bis(trifluoromethyl)-5-bromobenzobenzene (0.440 g, 1.5 mmol) and 4-(5,6-dihydropyrrolo[3,2,1-*ij*]quinolin-2-yl)benzonitrile **2** (0.258 g, 1 mmol), **25** was obtained in 73% (0.343 g) yield as a white solid: mp 209-211 °C.

^{1}H NMR (400 MHz, $CDCl_3$): δ 7.74-7.67 (m, 5H), 7.56 (d, J = 8.0 Hz, 1H), 7.45 (d, J = 8.5 Hz, 2H), 7.18 (t, J = 7.4 Hz, 1H), 7.10 (d, J = 7.2 Hz, 1H), 4.11 (t, J = 5.7 Hz, 2H), 3.09 (t, J = 5.1 Hz, 2H), 2.28 (quint., J = 5.7 Hz, 2H).

^{13}C NMR (100 MHz, $CDCl_3$): δ 137.4, 135.6, 134.9, 134.8, 132.7, 131.9 (q, J = 33.0 Hz), 131.3, 129.4, 124.4, 123.2 (q, J = 272.5 Hz), 122.8, 121.8, 120.8, 119.4 (q, J = 4.0 Hz), 118.4, 116.5, 113.2, 112.4, 43.7, 25.0, 23.0.

Elemental analysis: calcd (%) for $C_{26}H_{16}F_6N_2$ (470.42): C 66.38, H 3.43; found: C 66.20, H 3.54.

2-(2-(4-Cyanophenyl)-5,6-dihydropyrrolo[3,2,1-*ij*]quinolin-1-yl)benzonitrile (26)

From 2-bromobenzonitrile (0.272 g, 1.5 mmol) and 4-(5,6-dihydropyrrolo[3,2,1-*ij*]quinolin-2-yl)benzonitrile **2** (0.258 g, 1 mmol), **26** was obtained in 82% (0.294 g) yield as a white solid: mp 195-197 °C.

^{1}H NMR (400 MHz, $CDCl_3$): δ 7.66-7.53 (m, 5H), 7.41-7.34 (m, 4H), 7.13 (t, J = 7.2 Hz, 1H), 7.06 (d, J = 7.2 Hz, 1H), 4.31-4.18 (m, 1H), 4.18-4.05 (m, 1H), 3.14-3.05 (m, 2H), 2.40-2.27 (m, 1H), 2.27-2.21 (m, 1H).

^{13}C NMR (100 MHz, $CDCl_3$): δ 139.1, 136.0, 135.5, 134.8, 133.8, 132.8, 132.4, 132.2, 131.1, 127.1, 125.3, 122.6, 121.3, 120.5, 118.7, 118.6, 117.0, 113.6, 112.6, 111.7, 73.9, 25.0, 23.1.

Elemental analysis: calcd (%) for $C_{25}H_{17}N_3$ (359.43): C 83.54, H 4.77; found: C 83.31, H 4.40.

5,6-Dihydro-4*H*-dibenzo[a,c]pyrido[3,2,1-*jk*]carbazole-10-carbonitrile (27)

From 1,2-dibromobenzene (0.354 g, 1.5 mmol) and 4-(5,6-dihydropyrrolo[3,2,1-*ij*]quinolin-2-yl)benzonitrile **2** (0.258 g, 1 mmol), **27** was obtained in 62% (0.206 g) yield as a yellow solid: mp 262-264 °C.

^{1}H NMR (400 MHz, $CDCl_3$): δ 9.02 (s, 1H), 8.78 (d, J = 8.1 Hz, 1H), 8.60 (d, J = 8.3 Hz, 1H), 8.53 (d, J = 8.5 Hz, 1H), 8.34 (d, J = 8.1 Hz, 1H), 7.78 (t, J = 7.9 Hz, 1H), 7.73 (d, J = 8.5 Hz,

1H), 7.60 (t, *J* = 7.9 Hz, 1H), 7.33 (t, *J* = 7.8 Hz, 1H), 7.24 (d, *J* = 7.0 Hz, 1H), 4.77 (t, *J* = 5.7 Hz, 2H), 3.12 (t, *J* = 5.1 Hz, 2H), 2.36 (quint., *J* = 5.7 Hz, 2H).

^{13}C NMR (100 MHz, $CDCl_3$): δ 137.5, 132.2, 130.6, 130.1, 129.2, 128.5, 127.6, 126.2, 125.6, 124.4, 124.1, 123.5, 123.4, 122.8, 122.3, 121.2, 120.9, 120.0, 119.8, 115.9, 108.3, 46.8, 25.1, 23.7.

Elemental analysis: calcd (%) for $C_{24}H_{16}N_2$ (332.41): C 86.72, H 4.85; found: C 86.49, H 4.98.

(5,6-Dihydro-4*H*-dibenzo[*a,c*]pyrido[3,2,1-*jk*]carbazol-10-yl)(phenyl)methanone (28)

From 1,2-dibromobenzene (0.354 g, 1.5 mmol) and (4-(5,6-dihydropyrrolo[3,2,1-*ij*]quinolin-2-yl)phenyl)(phenyl)methanone **5** (0.337 g, 1 mmol), **28** was obtained in 55% (0.226 g) yield as a yellow solid: mp 126-128 °C.

^{1}H NMR (400 MHz, $CDCl_3$): δ 9.30 (s, 1H), 8.85 (d, *J* = 7.8 Hz, 1H), 8.74-8.67 (m, 2H), 8.40 (d, *J* = 8.1 Hz, 1H), 8.12 (dd, *J* = 8.6, 1.6 Hz, 1H), 7.95 (d, *J* = 7.9 Hz, 2H), 7.79 (t, *J* = 7.8 Hz, 1H), 7.68 (t, *J* = 7.9 Hz, 1H), 7.63-7.53 (m, 3H), 7.34 (d, *J* = 7.2 Hz, 1H), 7.28-7.21 (m, 1H), 4.96 (t, *J* = 5.7 Hz, 2H), 3.17 (t, *J* = 5.1 Hz, 2H), 2.42 (quint., *J* = 5.7 Hz, 2H).

^{13}C NMR (100 MHz, $CDCl_3$): δ 196.7, 138.2, 137.5, 133.7, 133.0, 132.5, 130.6, 130.3, 129.9, 128.6, 127.9, 127.3, 127.0, 126.9, 126.7, 124.1, 124.0, 123.7, 122.9, 122.8, 121.9, 121.5, 120.7, 119.9, 115.5, 46.9, 25.2, 23.8.

Elemental analysis: calcd (%) for $C_{30}H_{21}NO$ (411.50): C 87.56, H 5.14; found: C 87.39, H 4.95.

13,14-Dihydro-12*H*-benzo[*c*]dipyrido[4,3-a:3',2',1'-*jk*]carbazole (29)

From 1,2-dibromobenzene (0.354 g, 1.5 mmol) and 2-(pyridin-4-yl)-5,6-dihydropyrrolo[3,2,1-*ij*]quinoline **17** (0.234 g, 1 mmol), **29** was obtained in 60% (0.185 g) yield as a yellow solid: mp 239-241 °C.

^{1}H NMR (400 MHz, $CDCl_3$): δ 10.16 (bs, 1H), 8.90-8.79 (m, 2H), 8.77 (bs, 1H), 8.42-8.32 (m, 2H), 7.80 (t, *J* = 7.6 Hz, 1H), 7.65 (t, *J* = 7.6 Hz, 1H), 7.35 (t, *J* = 7.5 Hz, 1H), 7.26 (d, *J* = 7.0 Hz, 1H), 4.91 (t, *J* = 5.7 Hz, 2H), 3.17 (t, *J* = 5.1 Hz, 2H), 2.43 (quint., *J* = 5.7 Hz, 2H).

^{13}C NMR (100 MHz, $CDCl_3$): δ 147.6, 144.6, 137.6, 131.1, 130.6, 128.2, 125.6, 124.6, 124.1, 122.9, 122.8, 122.4, 121.2, 120.9, 120.1, 116.7, 46.4, 25.0, 23.6.

Elemental analysis: calcd (%) for $C_{22}H_{16}N_2$ (308.38): C 85.69, H 5.23; found: C 85.50, H 4.96.

[1] Cantat, T.; Génin, E.; Giroud, C.; Meyer G.; Jutand, A. *J. Organomet. Chem.* **2003**, *687*, 365-376.

3-(5,6-Dihydropyrrolo[3,2,1-*ij*]quinolin-2-yl)benzonitrile (1a)

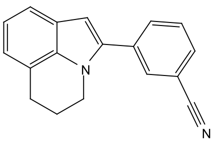

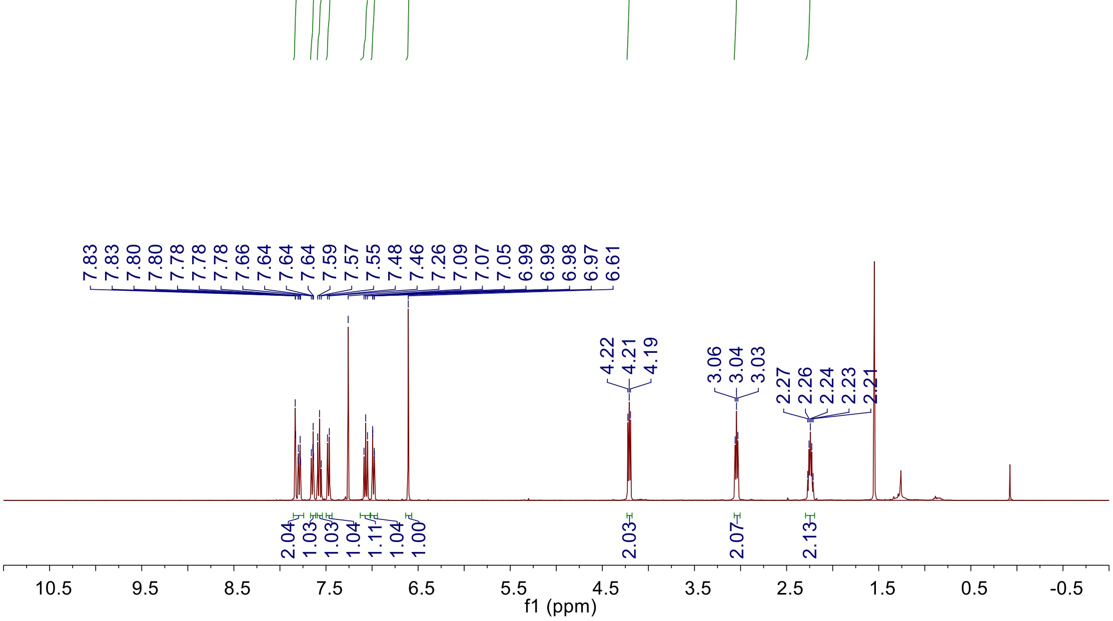

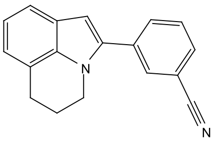

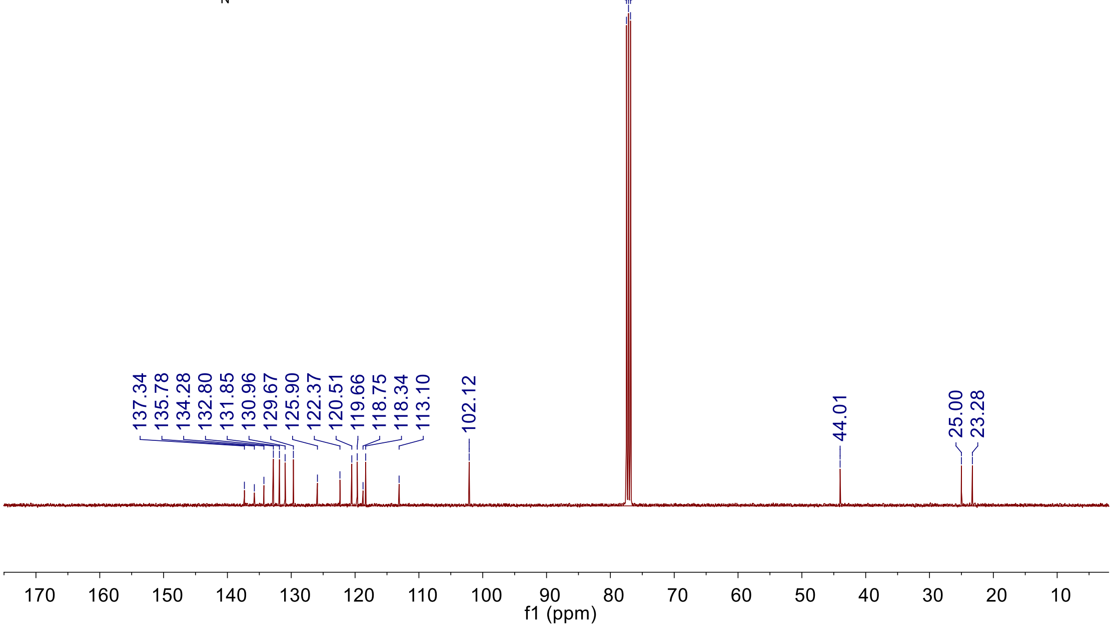

4-(5,6-Dihydropyrrolo[3,2,1-*ij*]quinolin-2-yl)benzonitrile (2)

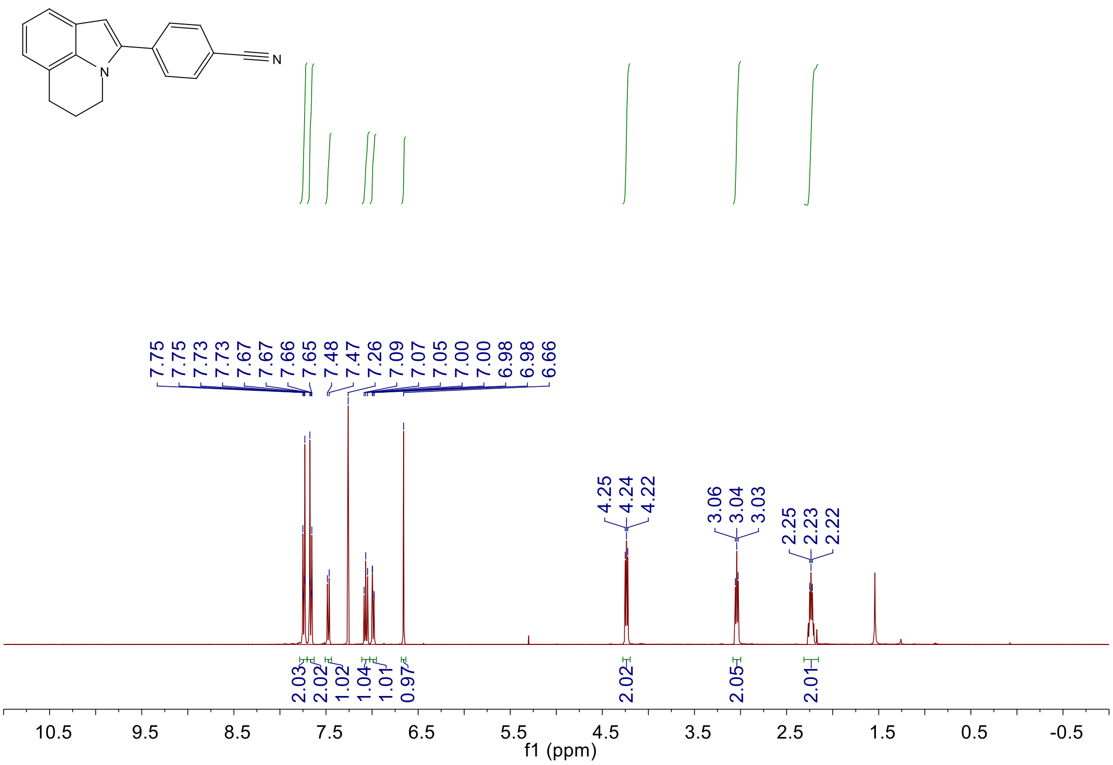

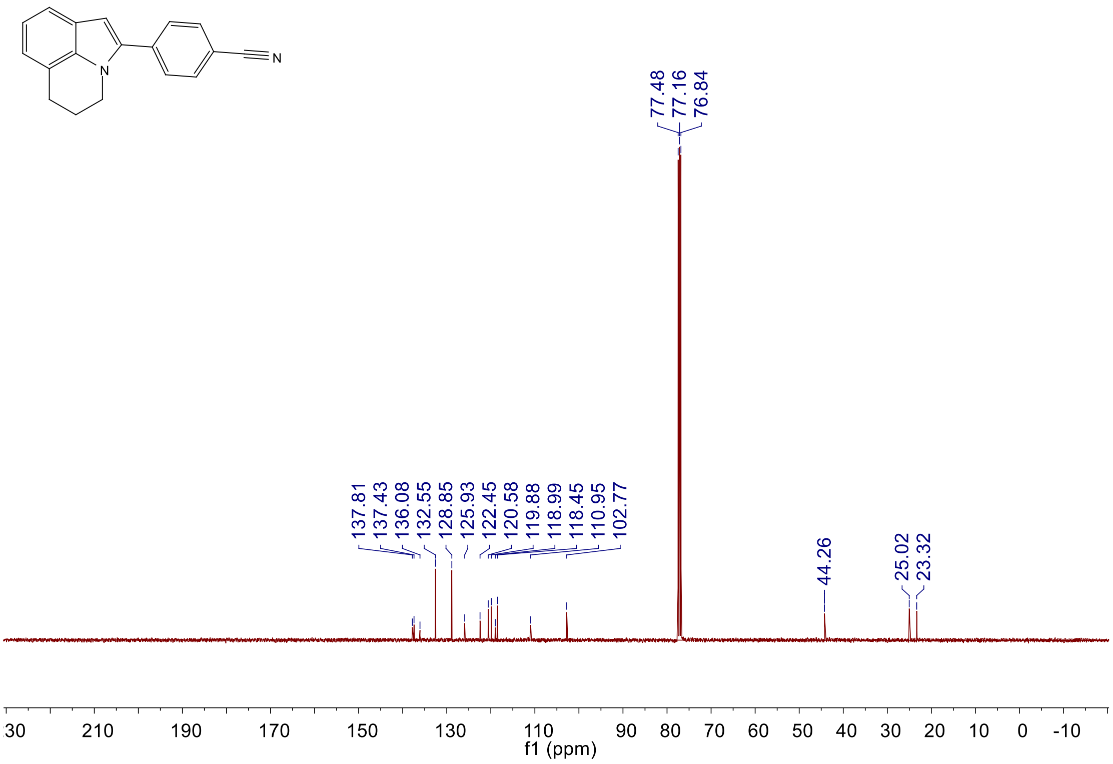

1-(4-(5,6-Dihydropyrrolo[3,2,1-*ij*]quinolin-2-yl)phenyl)ethan-1-one (3)

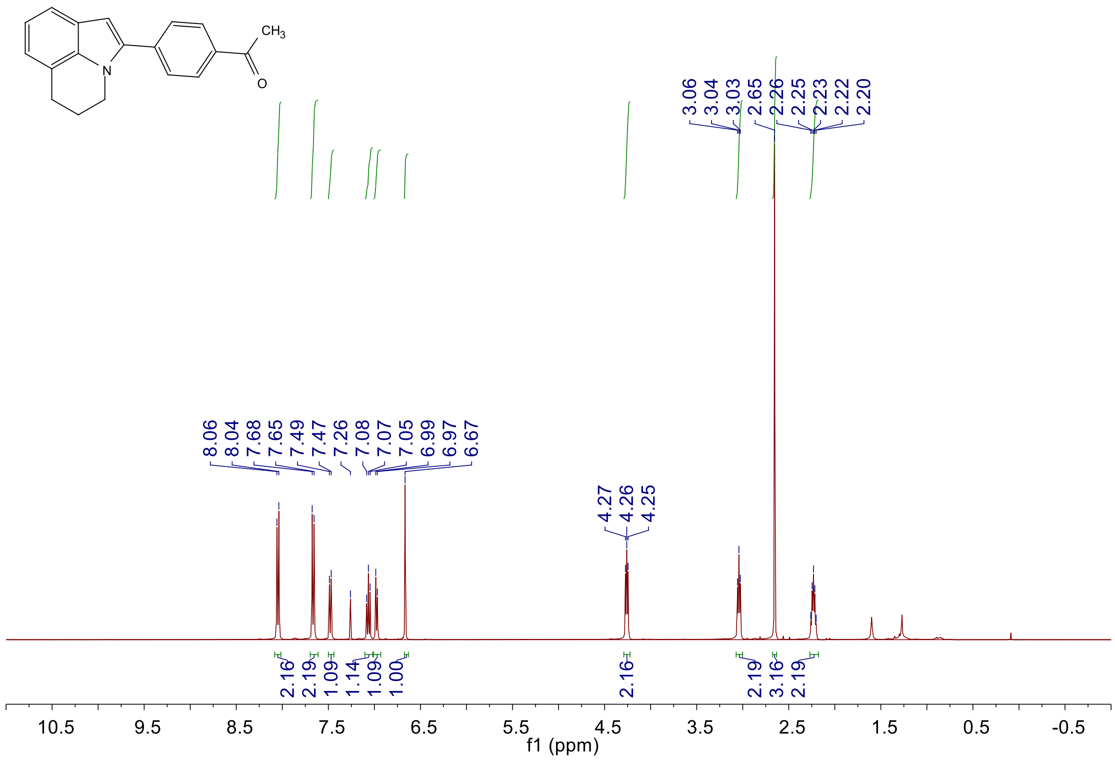

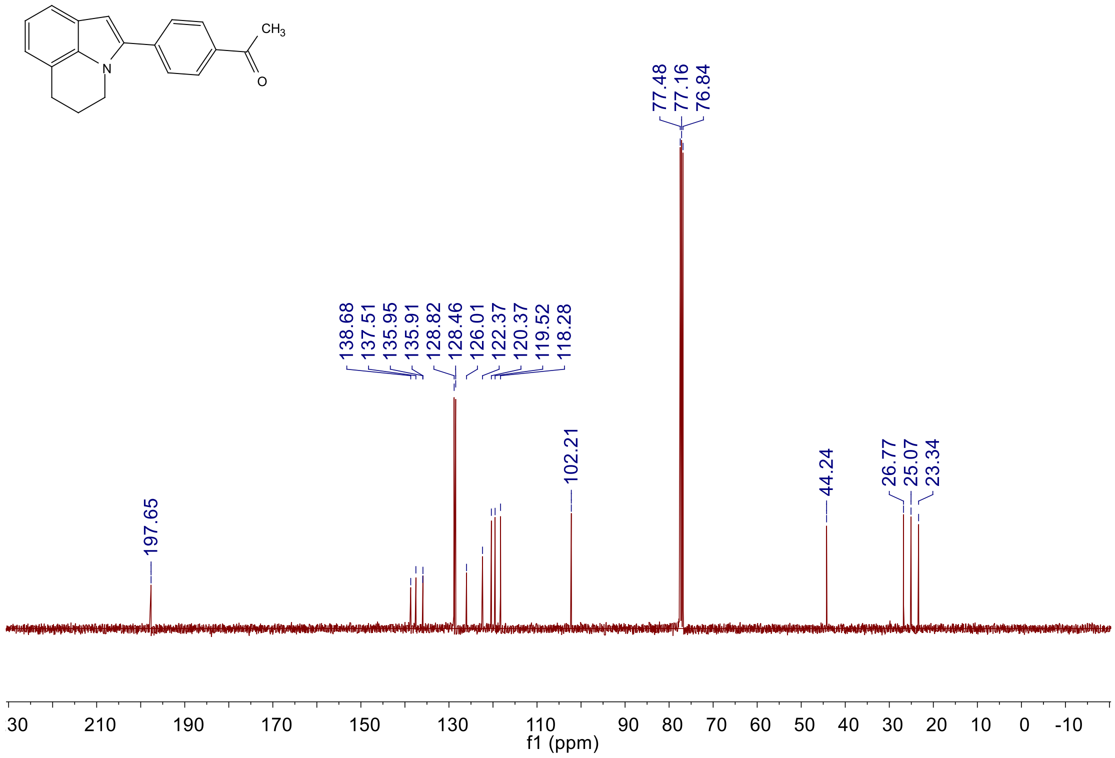

1-(4-(5,6-Dihydropyrrolo[3,2,1-*ij*]quinolin-2-yl)phenyl)propan-1-one (4)

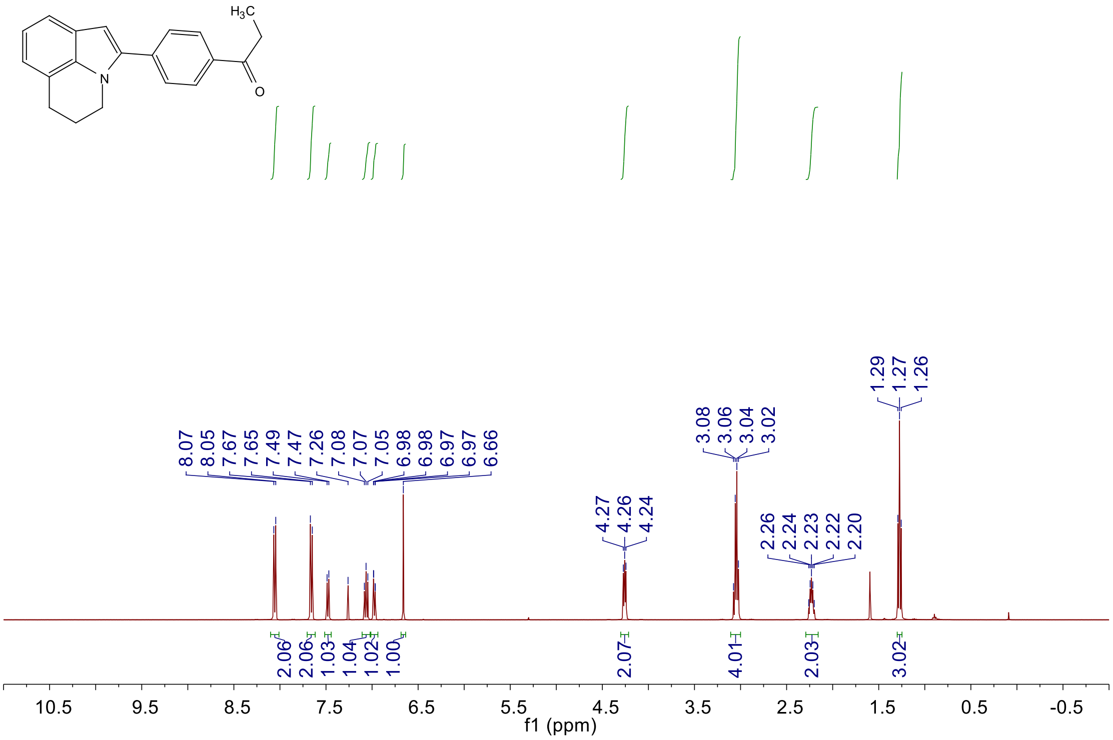

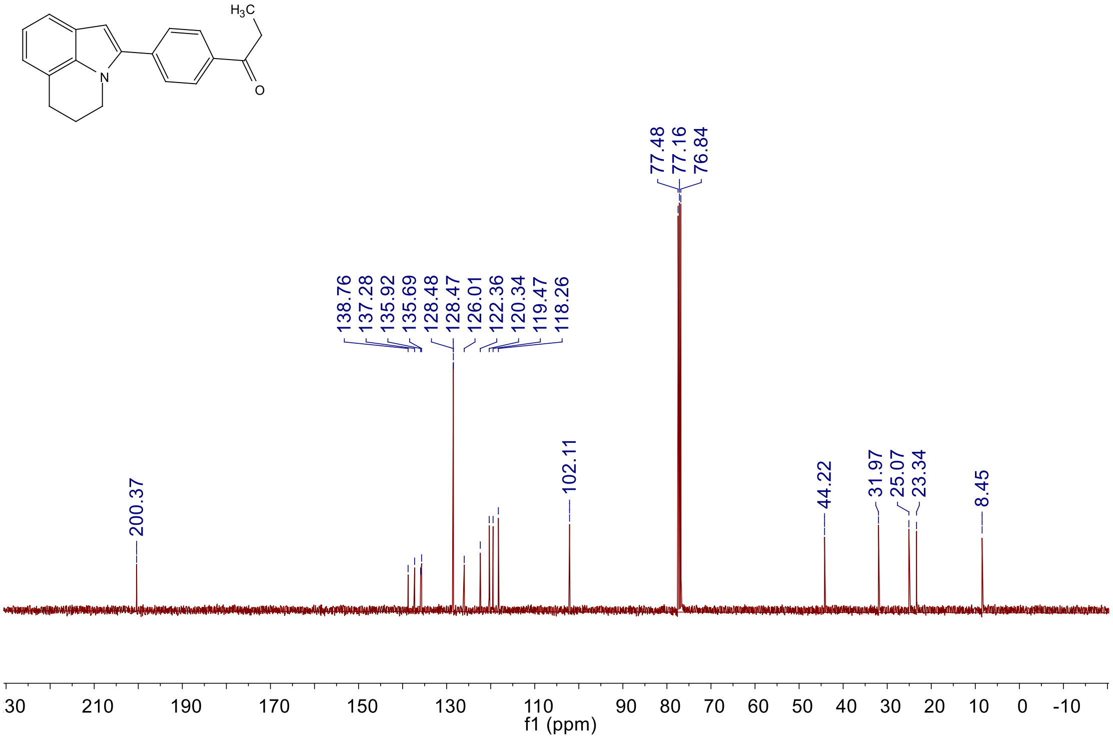

Other regioisomer: 1-(4-(5,6-Dihydropyrrolo[3,2,1-*ij*]quinolin-1-yl)phenyl)propan-1-one

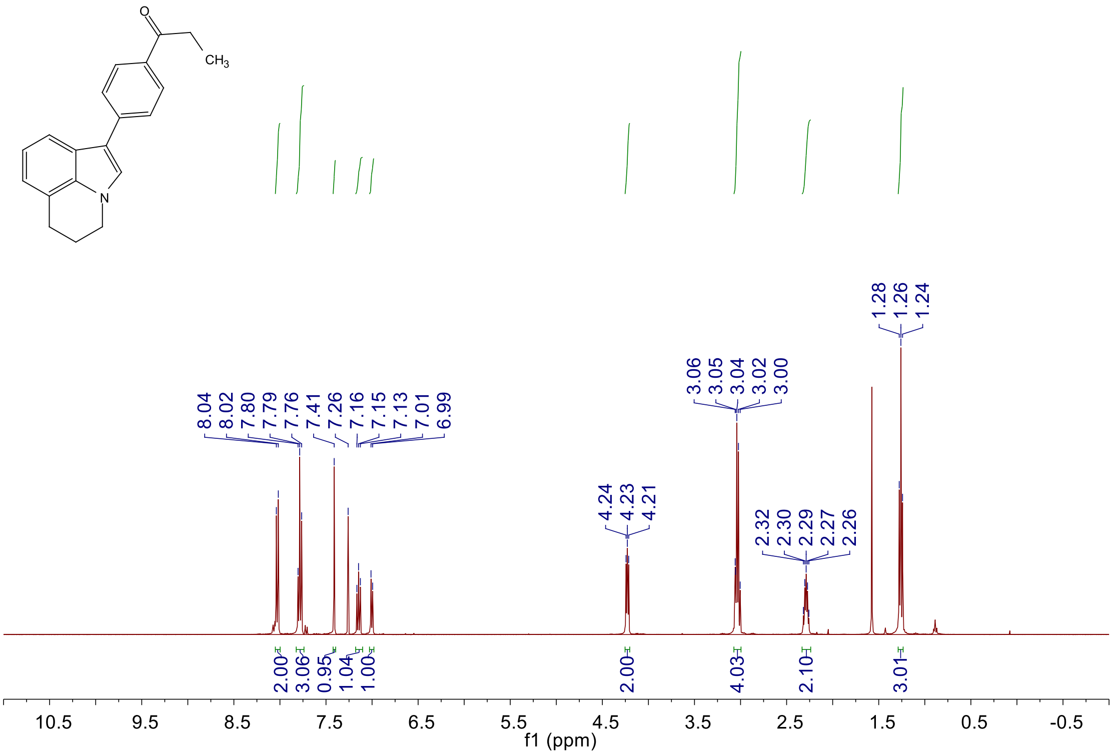

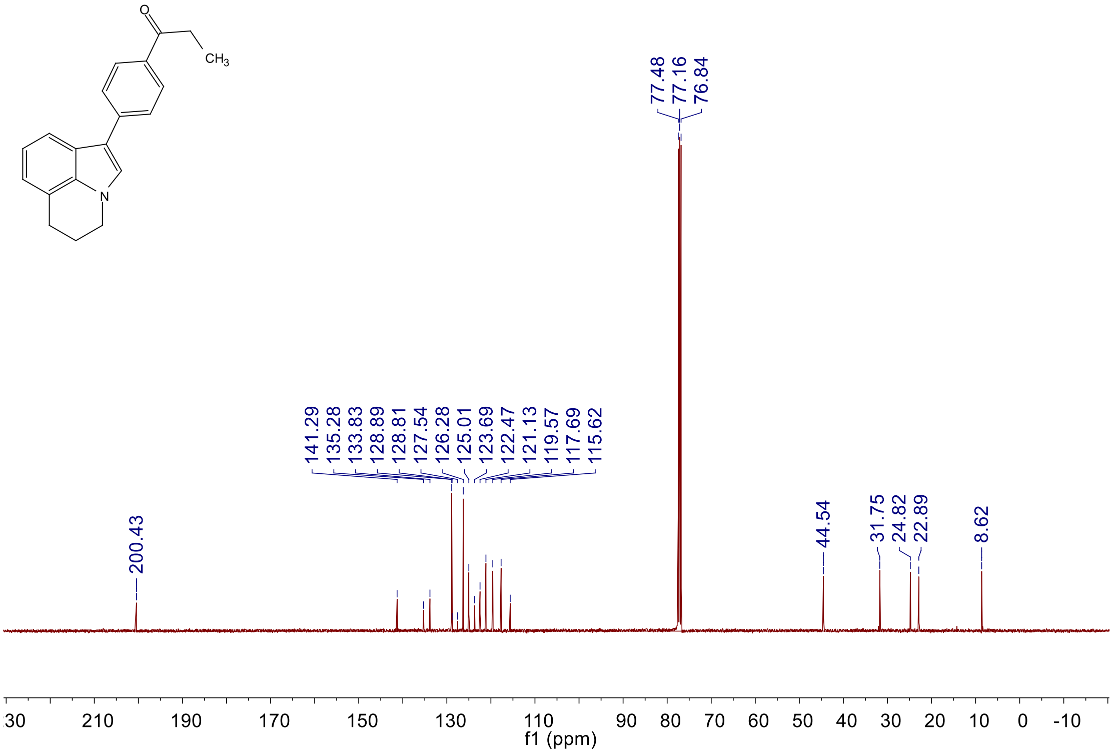

(4-(5,6-Dihydropyrrolo[3,2,1-*ij*]quinolin-2-yl)phenyl)(phenyl)methanone (5)

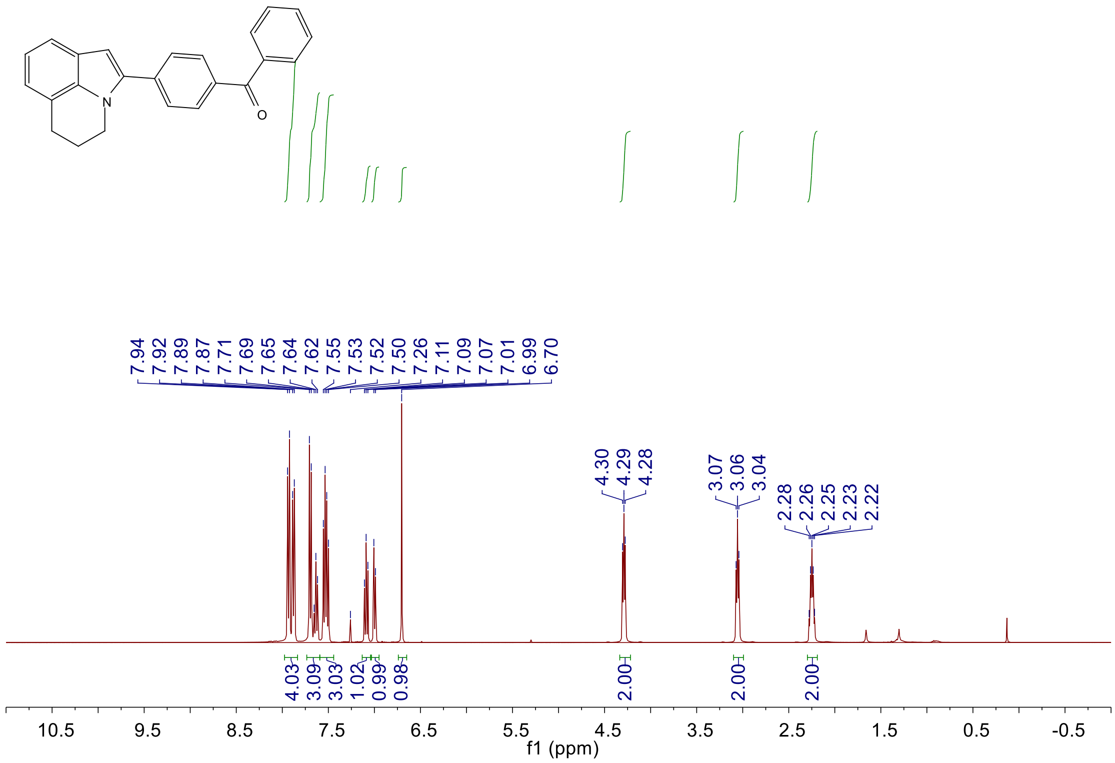

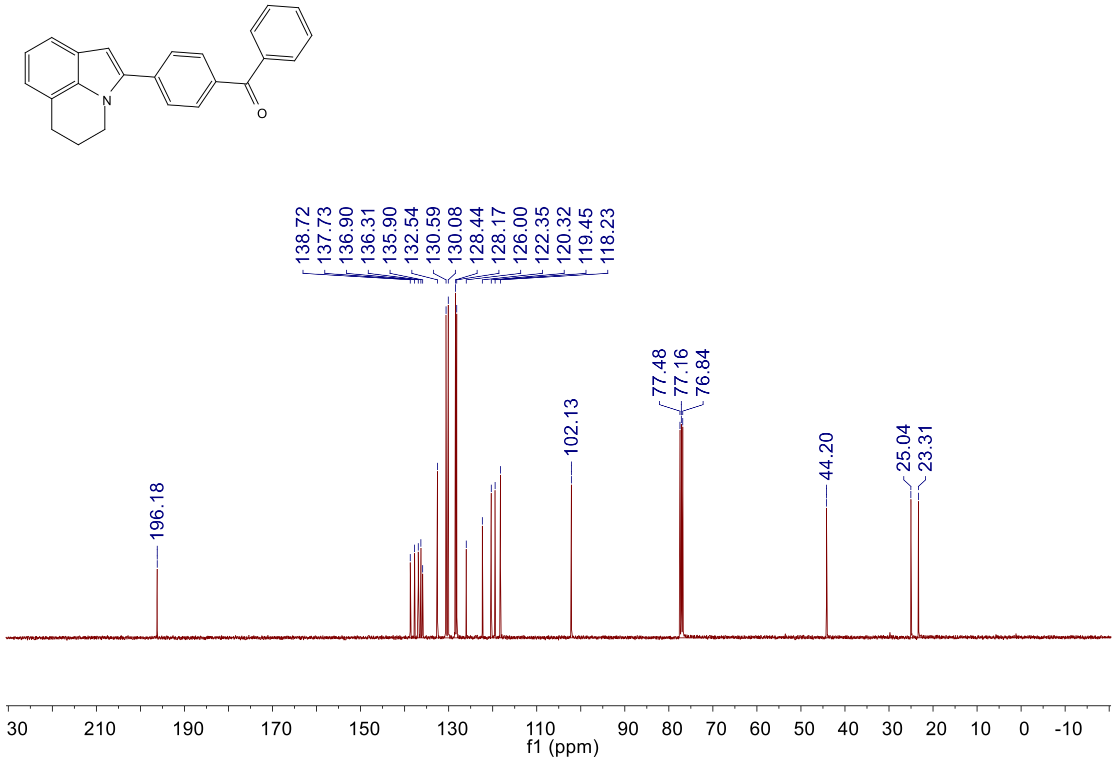

Ethyl 4-(5,6-Dihydropyrrolo[3,2,1-*ij*]quinolin-2-yl)benzoate (6)

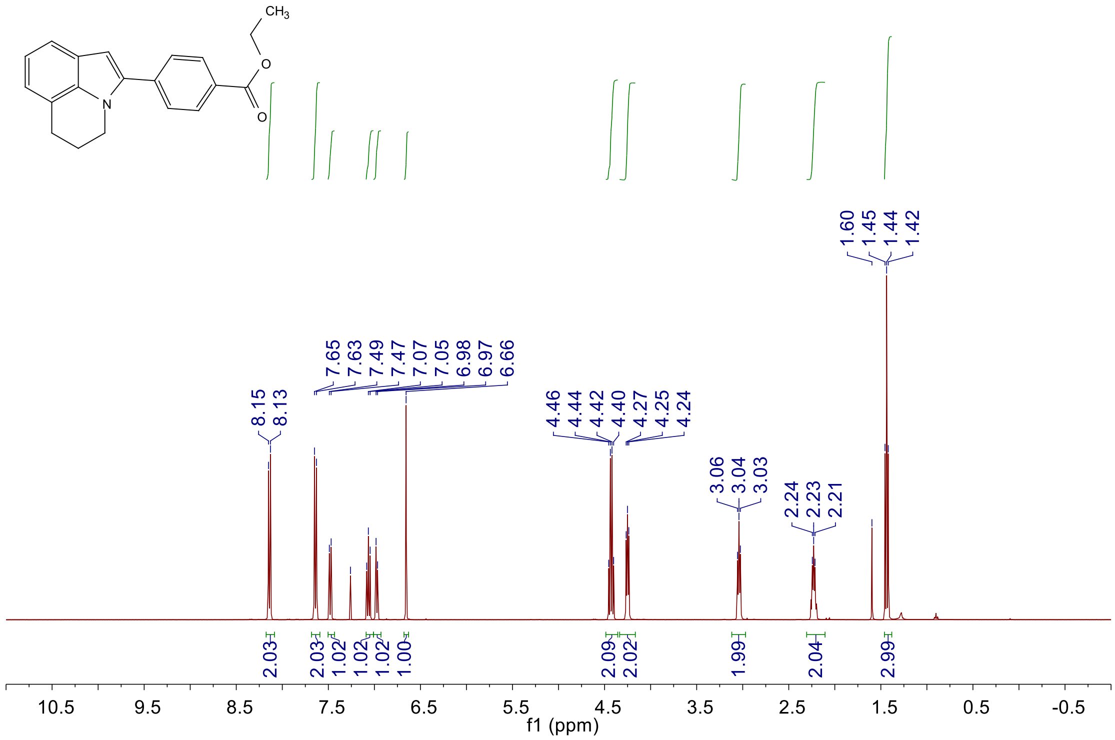

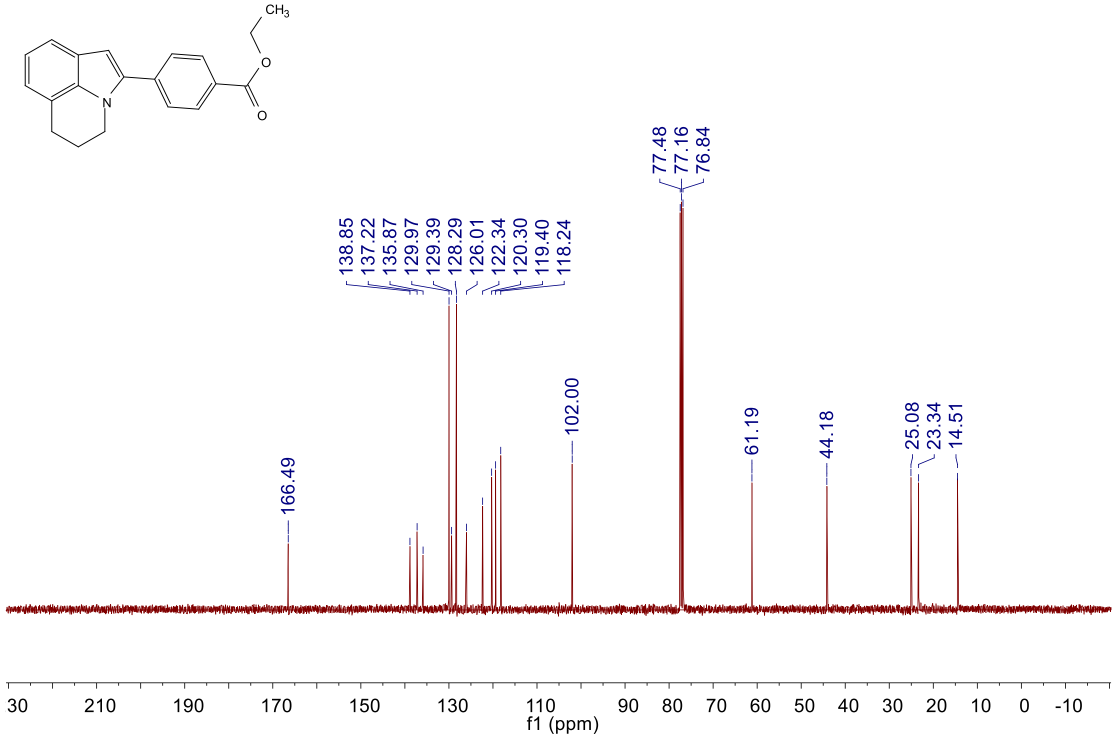

4-(5,6-Dihydropyrrolo[3,2,1-*ij*]quinolin-2-yl)benzaldehyde (7)

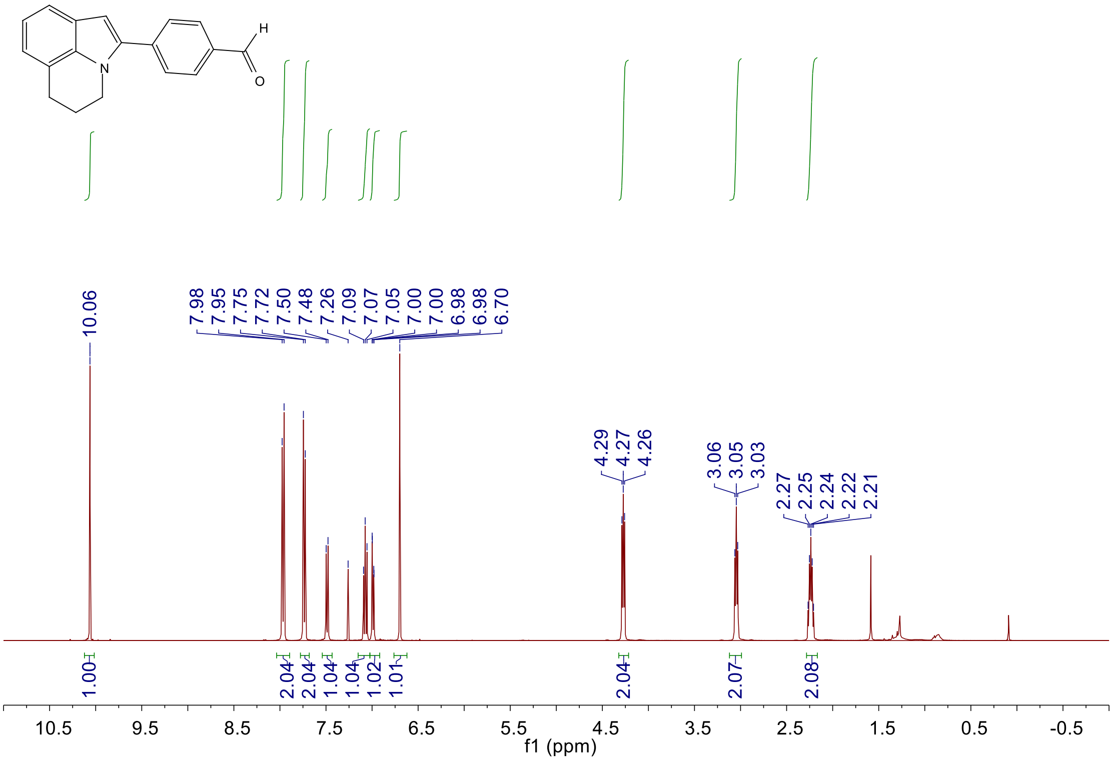

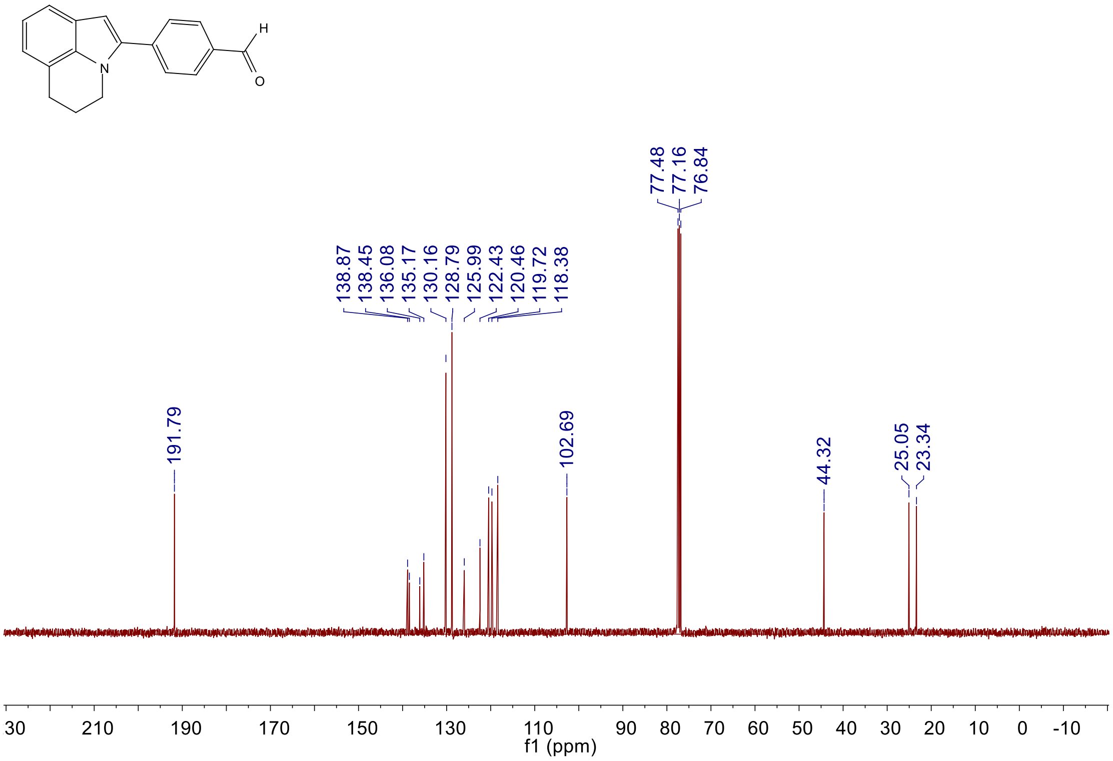

2-(4-Chlorophenyl)-5,6-dihydropyrrolo[3,2,1-*ij*]quinoline (8)

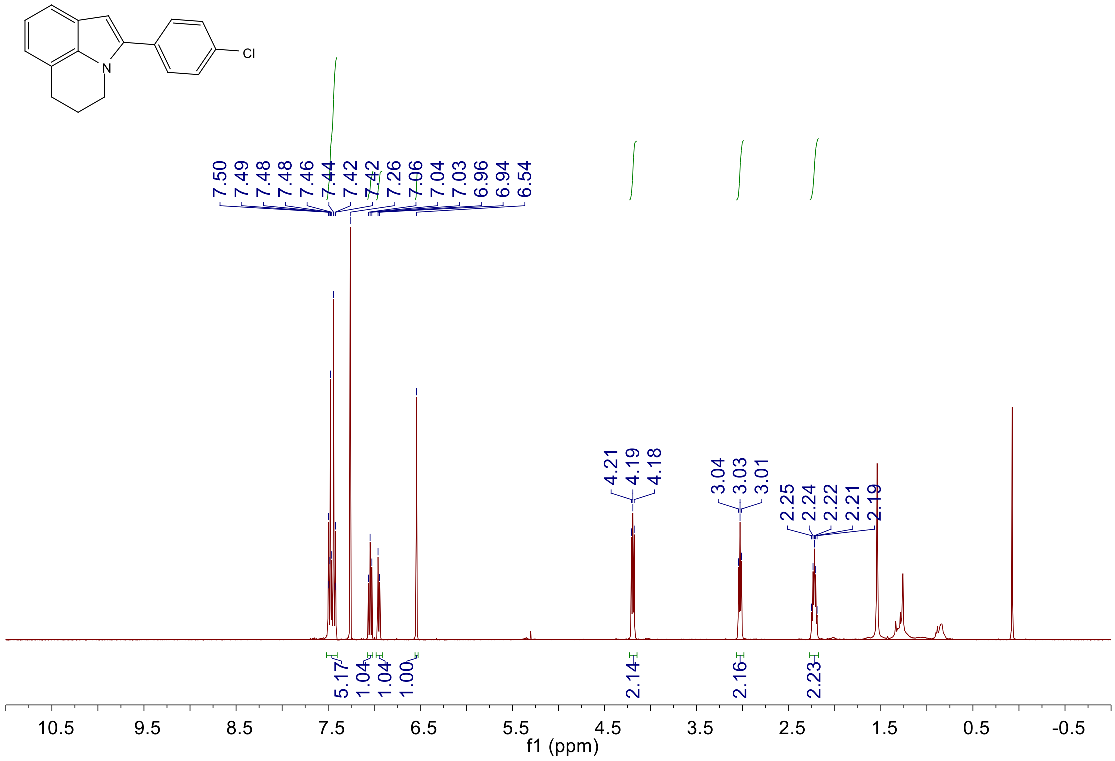

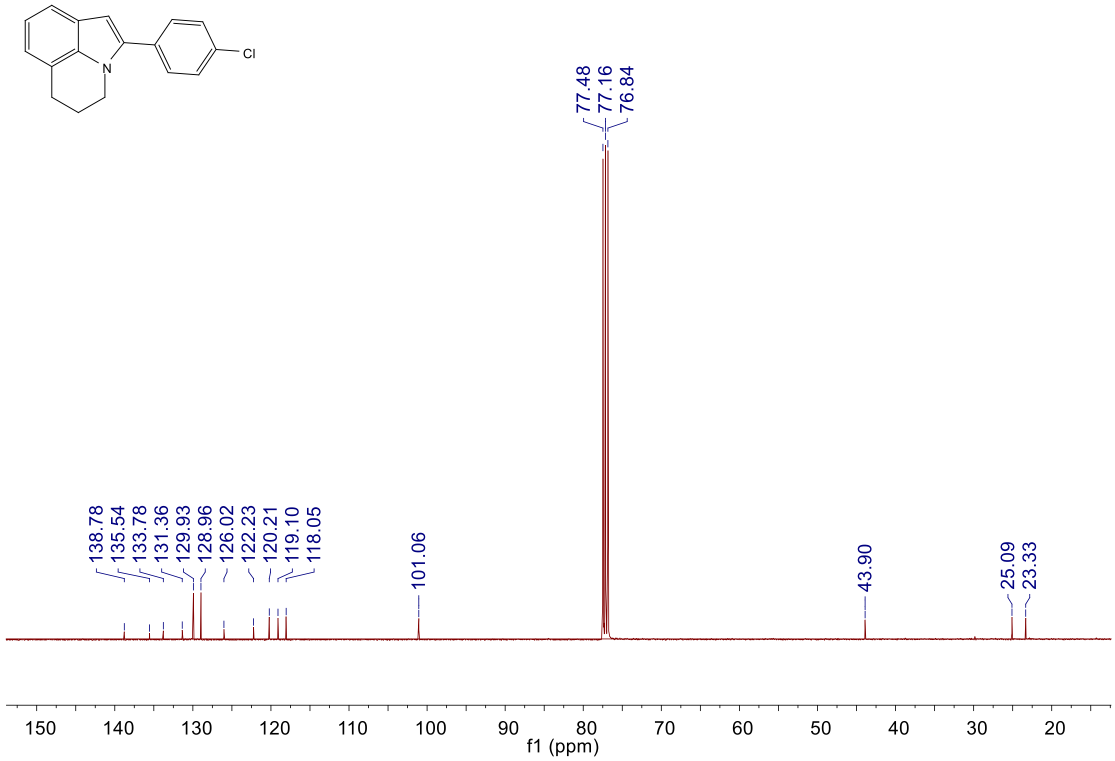

2-(4-(5,6-Dihydropyrrolo[3,2,1-*ij*]quinolin-2-yl)phenyl)acetonitrile (9)

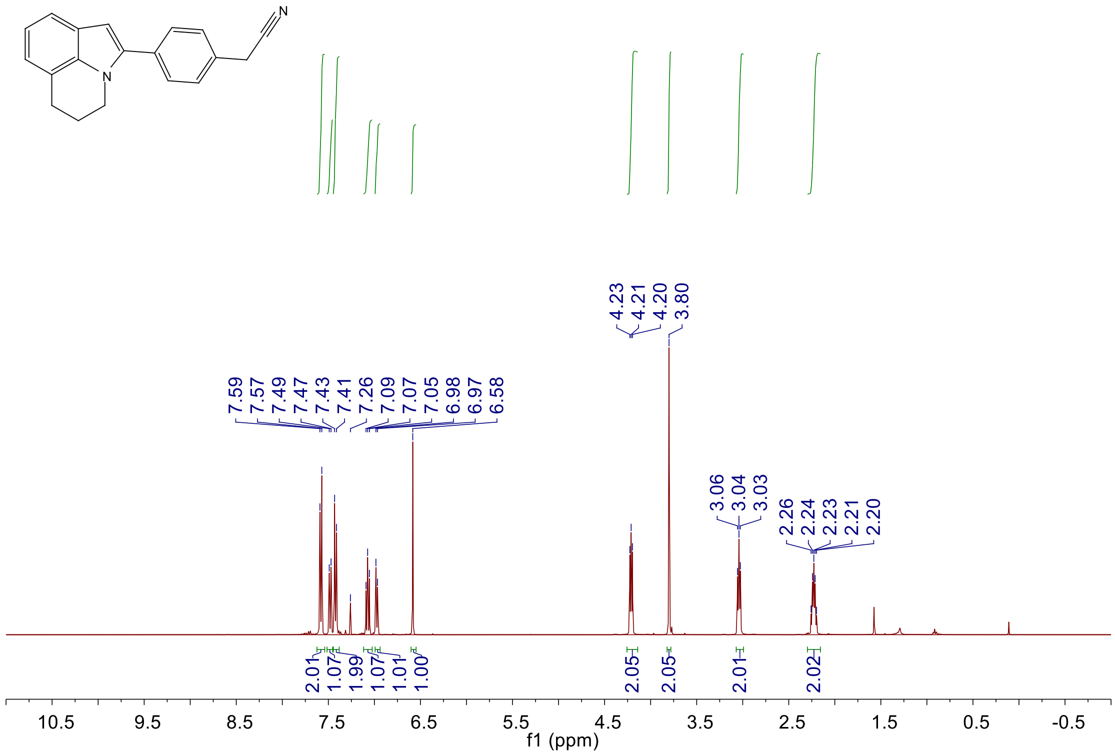

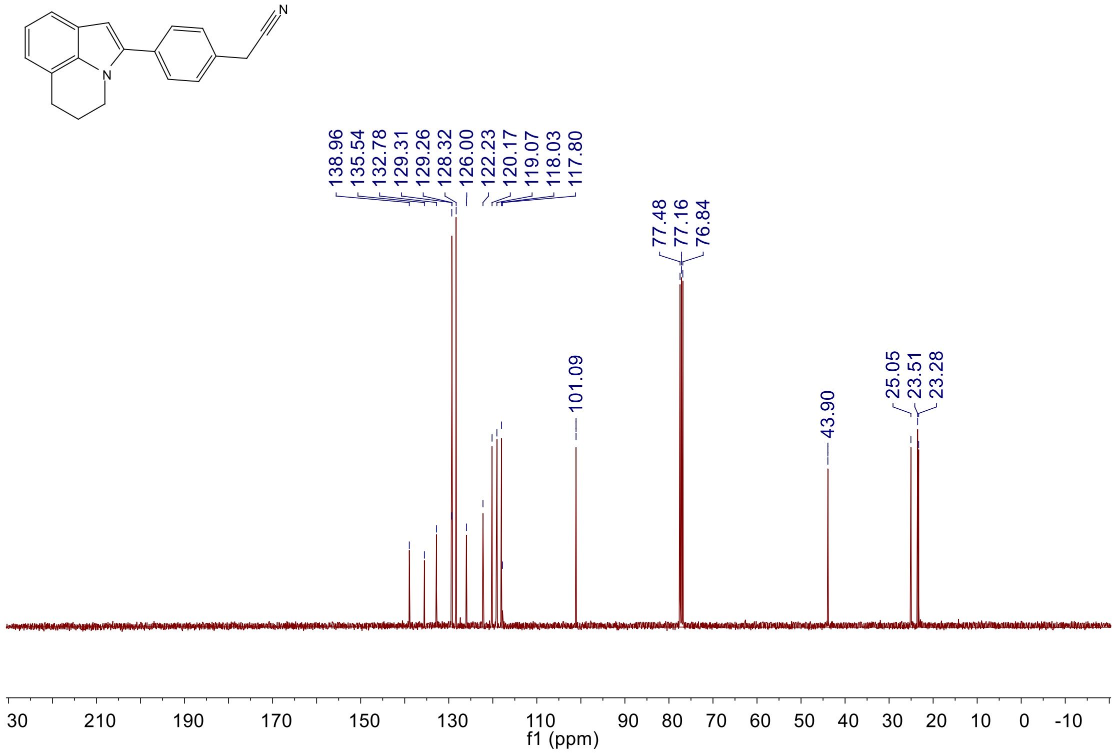

1-(3-(5,6-Dihydropyrrolo[3,2,1-*ij*]quinolin-2-yl)phenyl)ethan-1-one (10)

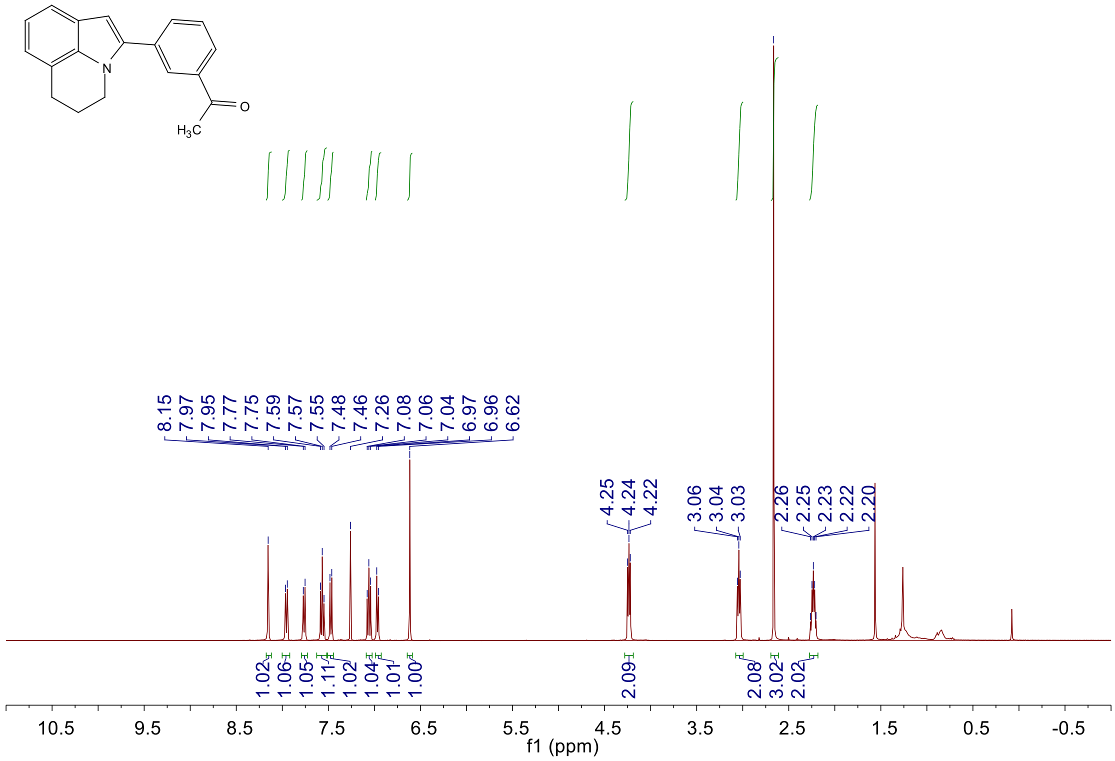

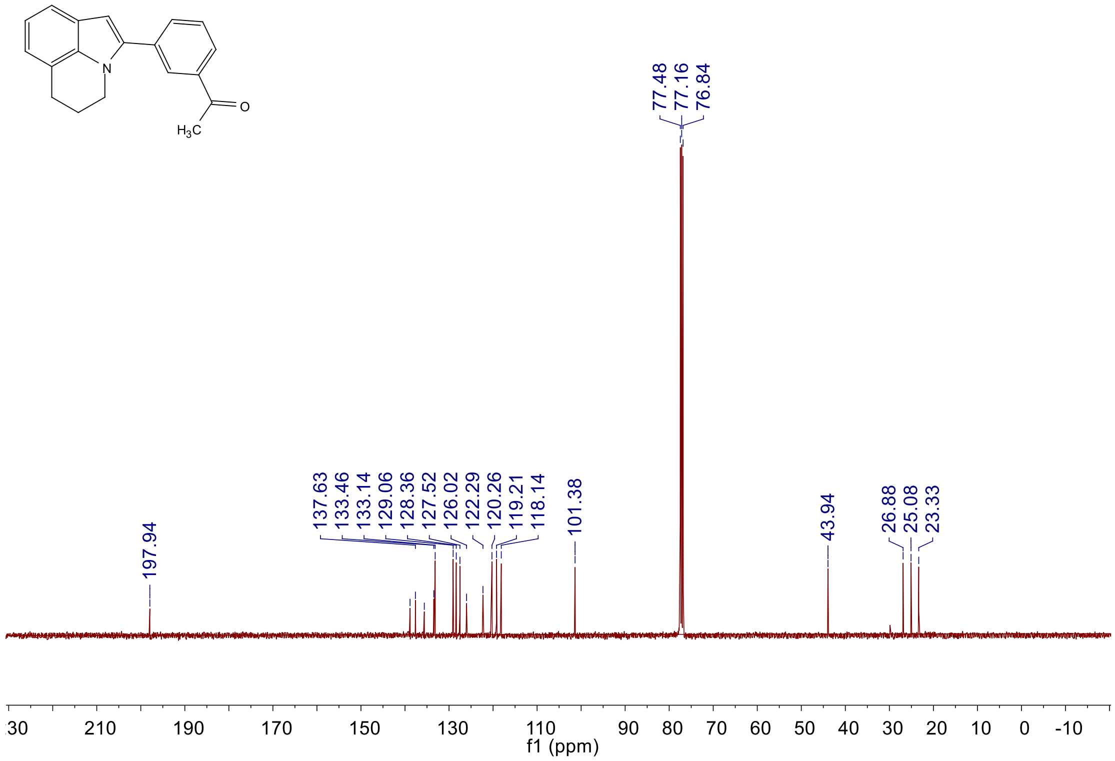

Methyl 3-(5,6-Dihydropyrrolo[3,2,1-*ij*]quinolin-2-yl)benzoate (11)

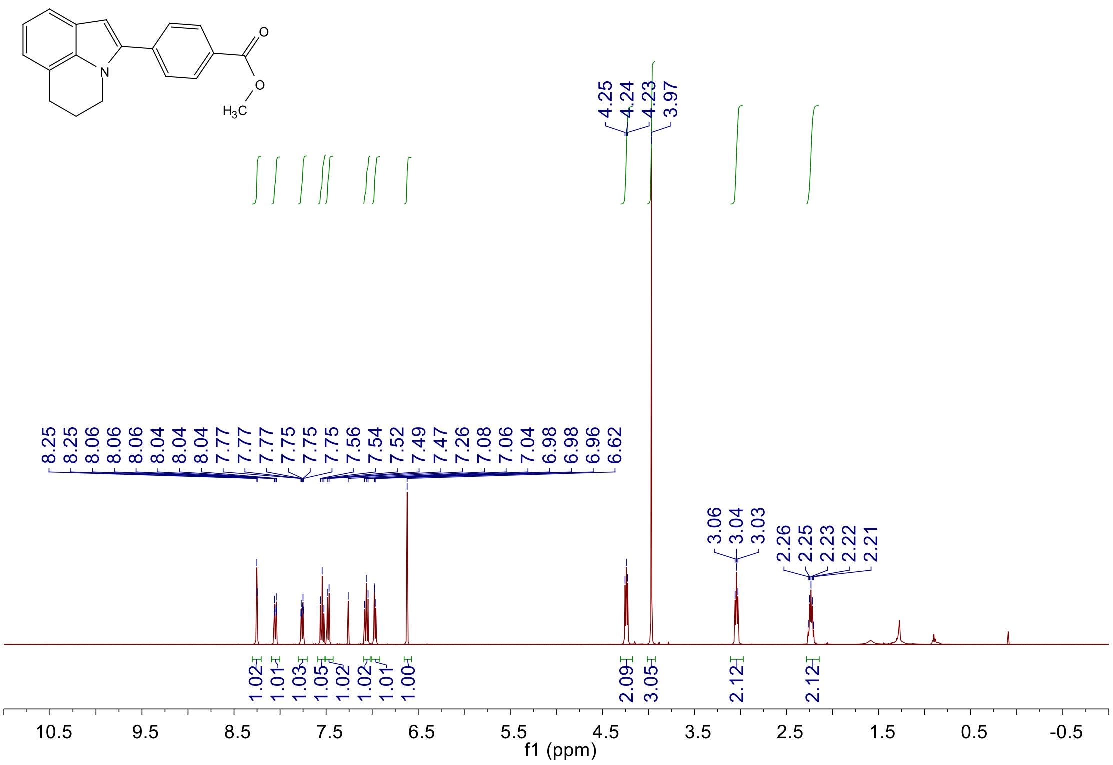

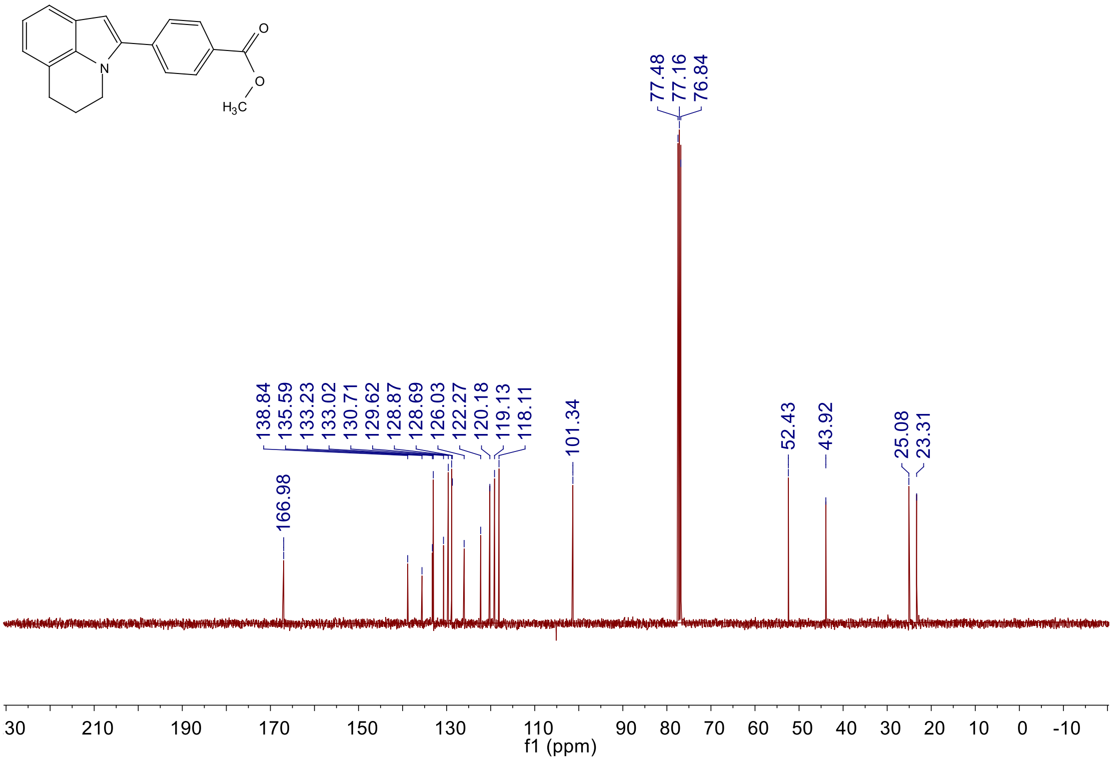

2-(5,6-Dihydropyrrolo[3,2,1-*ij*]quinolin-2-yl)benzonitrile (12)

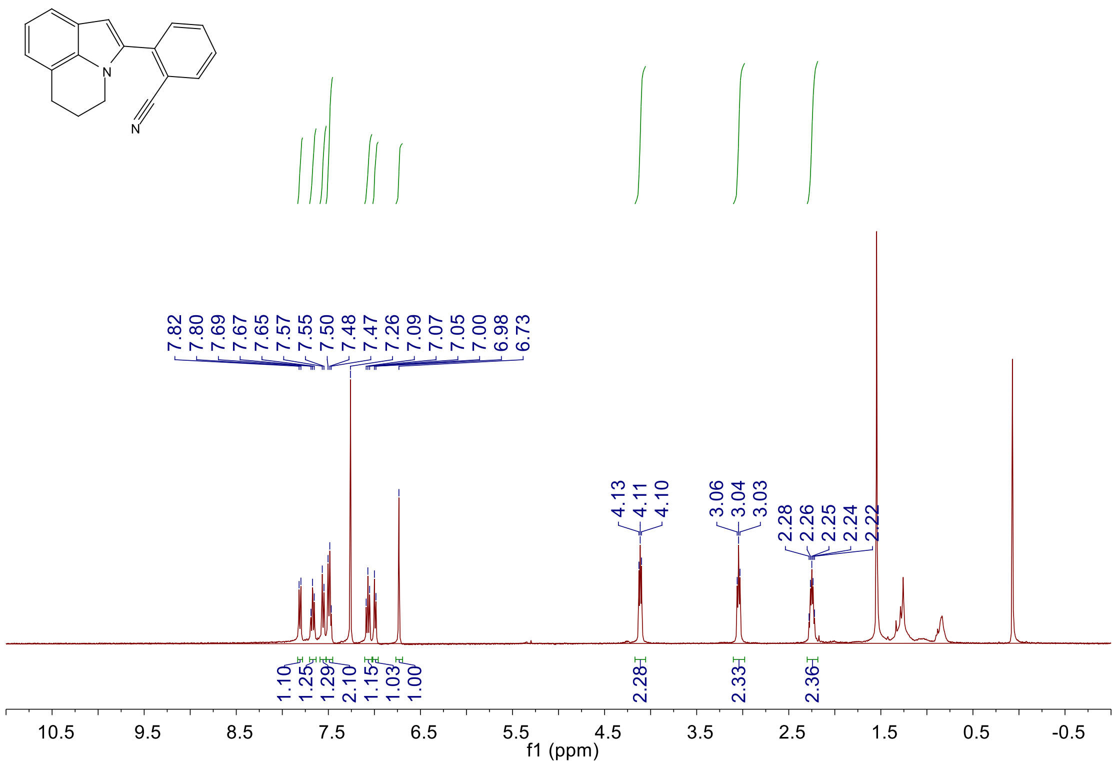

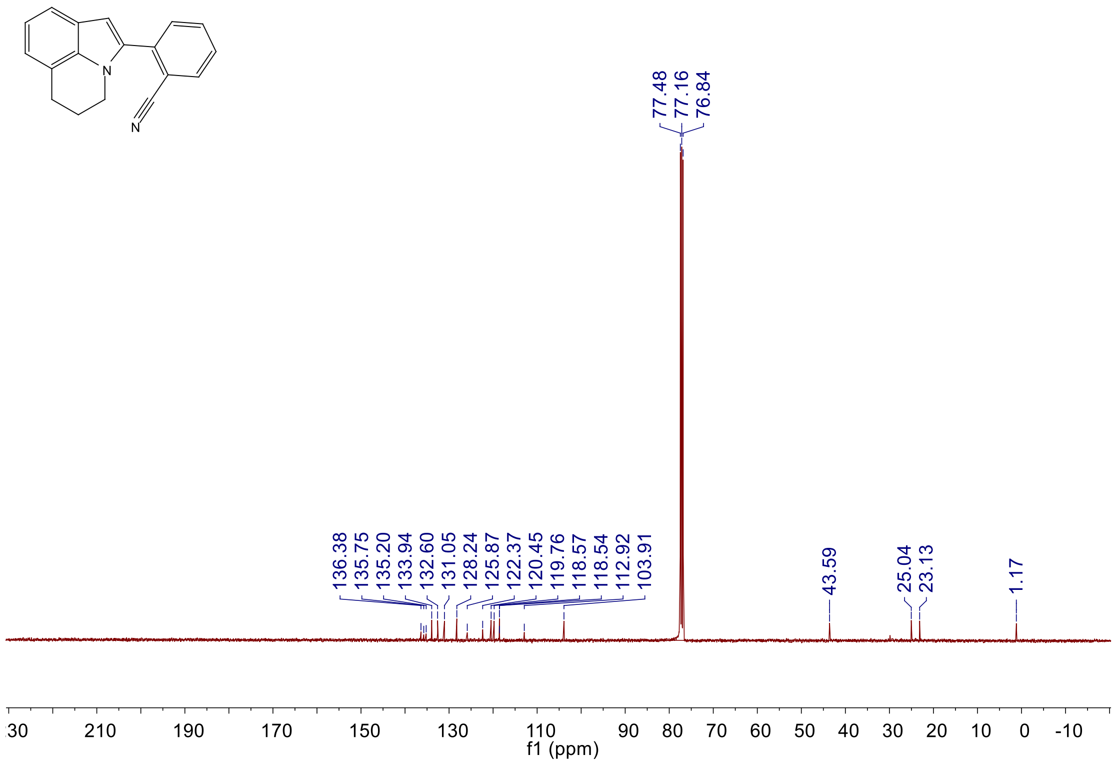

Other regioisomer: 2-(5,6-Dihydropyrrolo[3,2,1-*ij*]quinolin-1-yl)benzonitrile

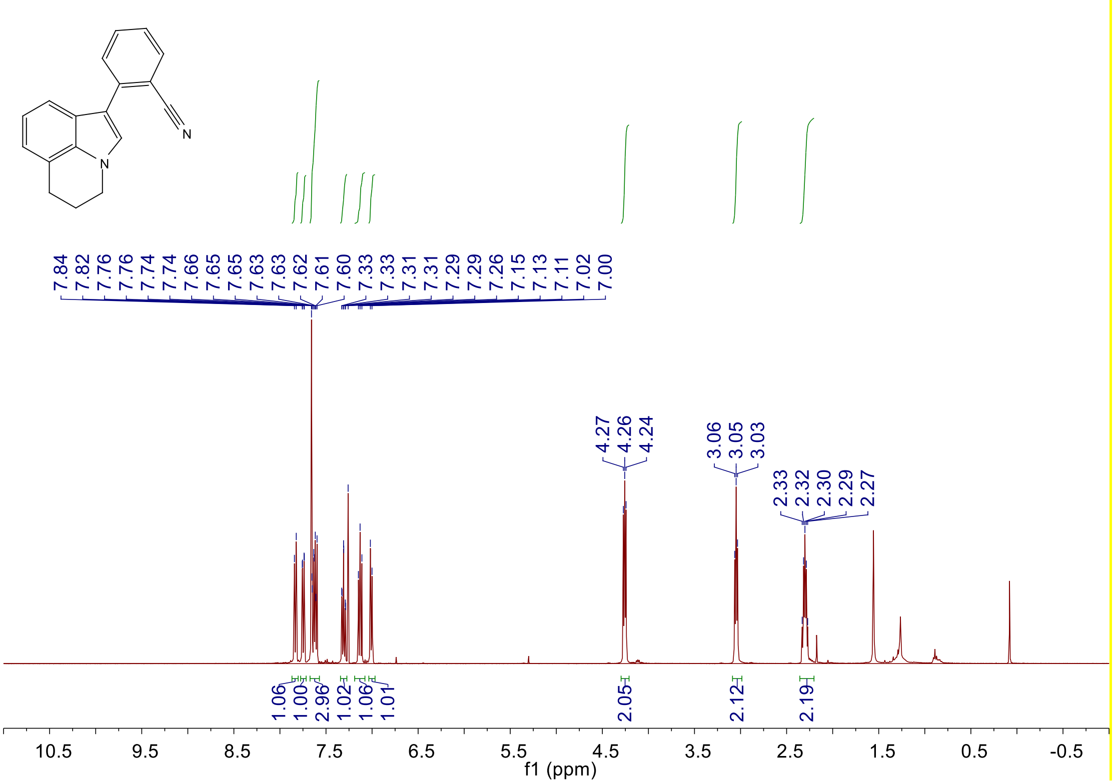

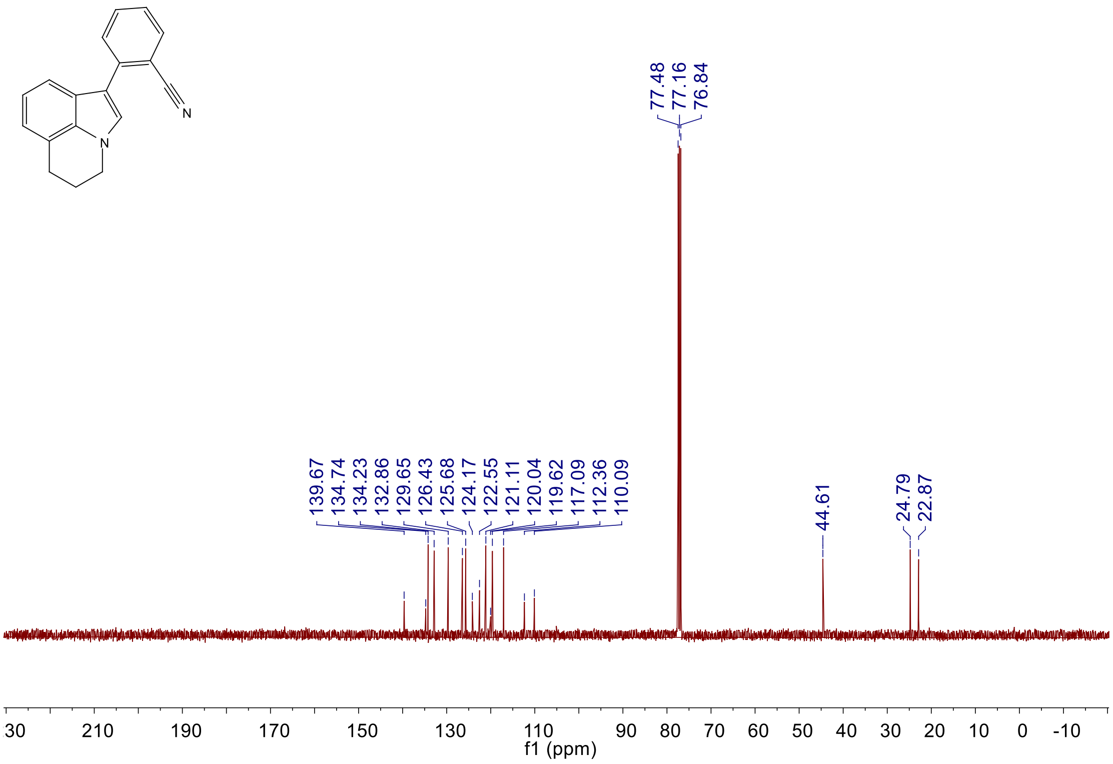

2-(4-(*tert*-Butyl)phenyl)-5,6-dihydropyrrolo[3,2,1-*ij*]quinoline (13)

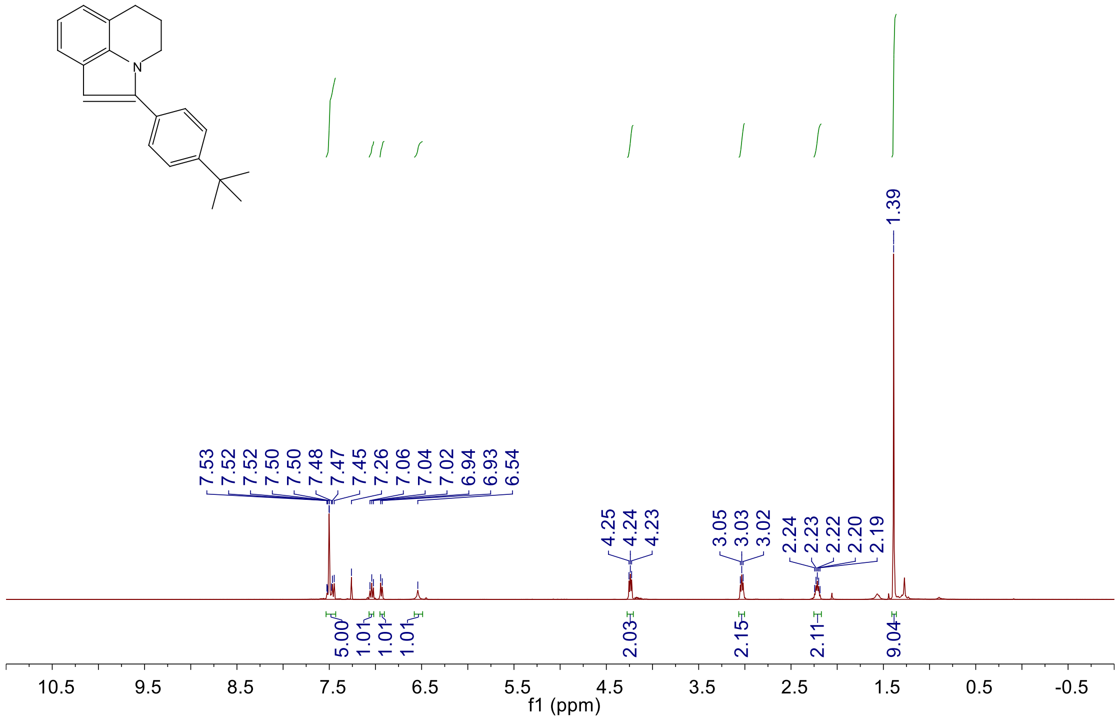

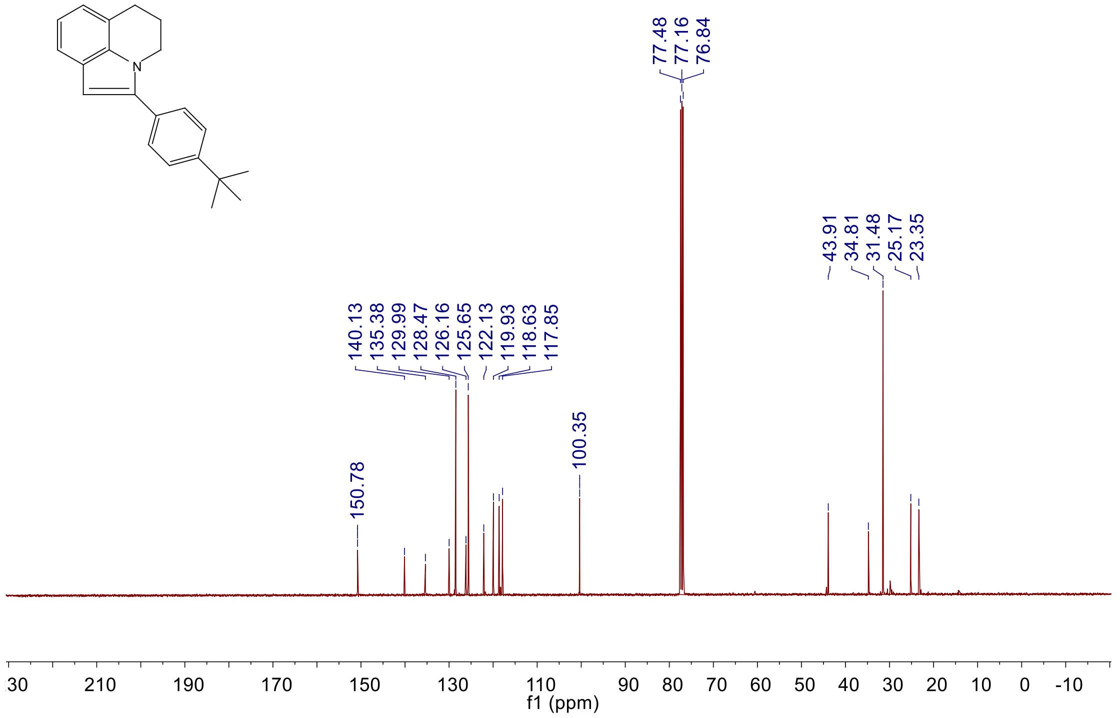

2-(4-Methoxyphenyl)-5,6-dihydropyrrolo[3,2,1-*ij*]quinoline (14)

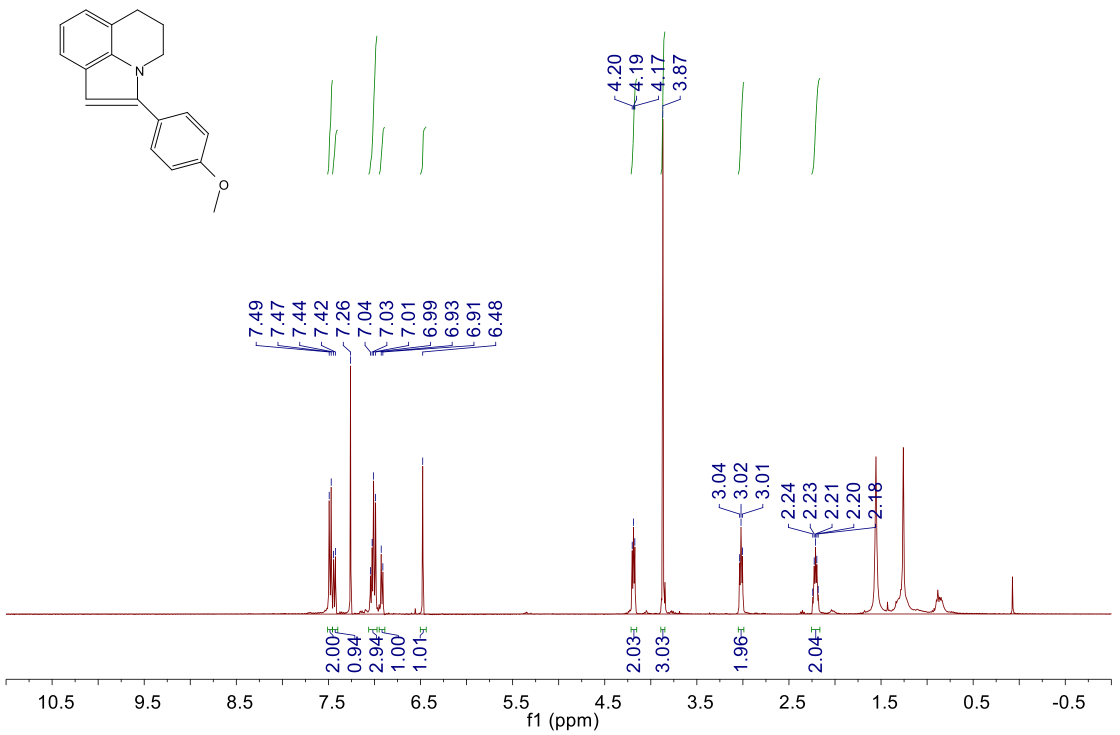

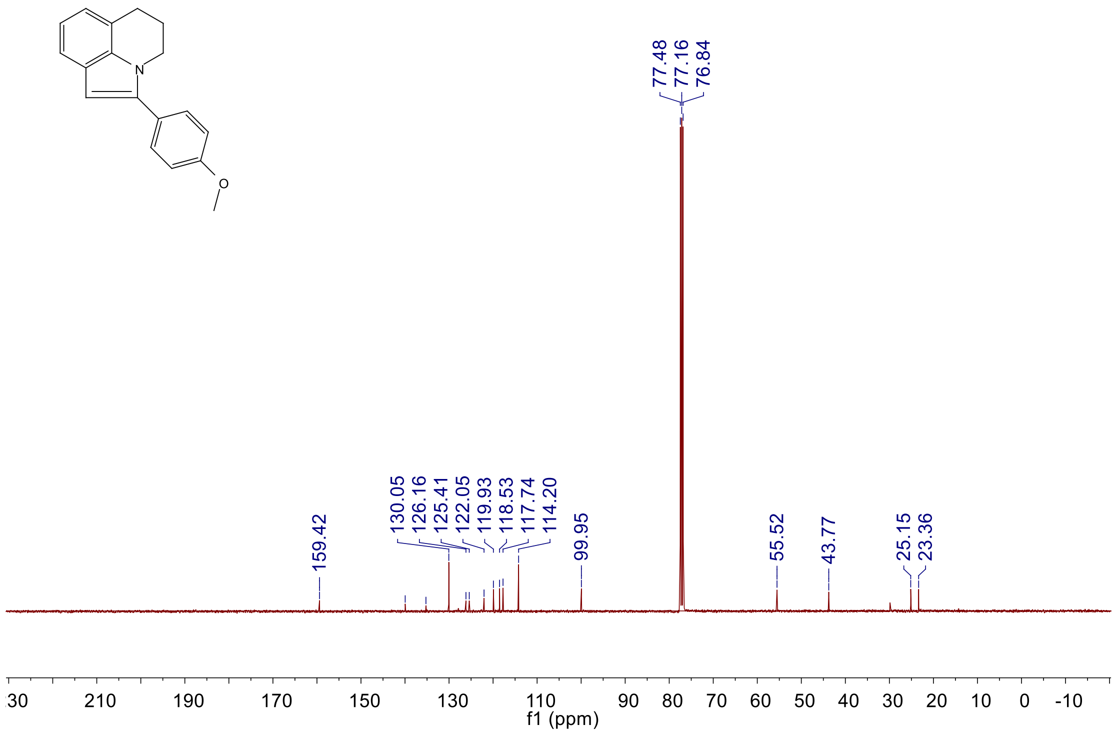

2-(Naphthalen-2-yl)-5,6-dihydropyrrolo[3,2,1-*ij*]quinoline (15)

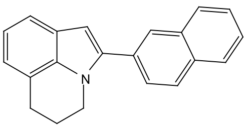

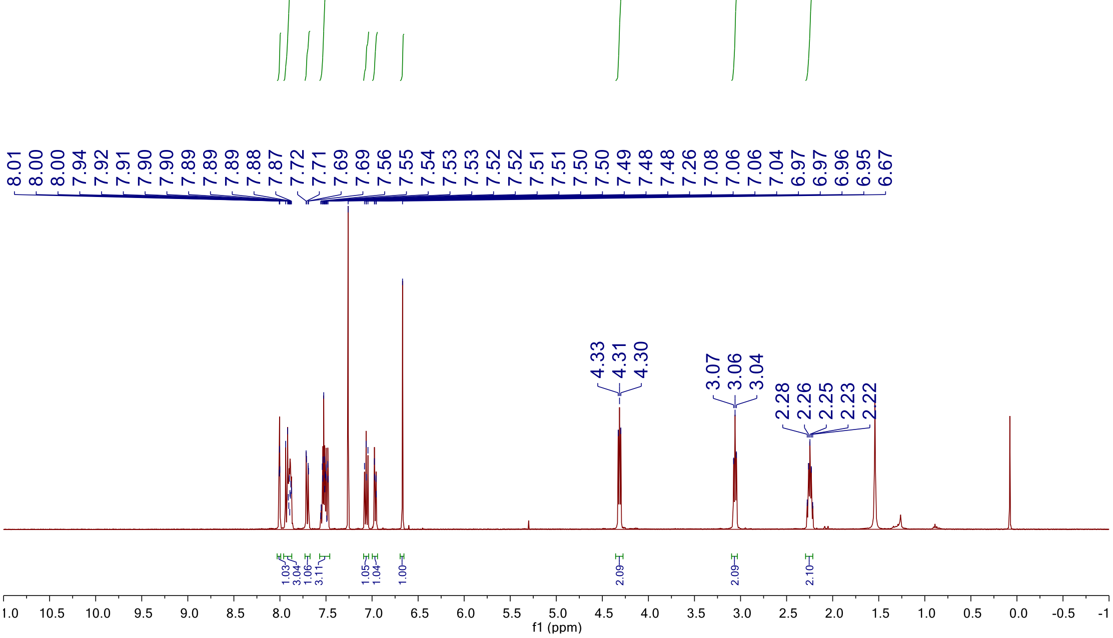

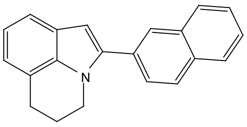

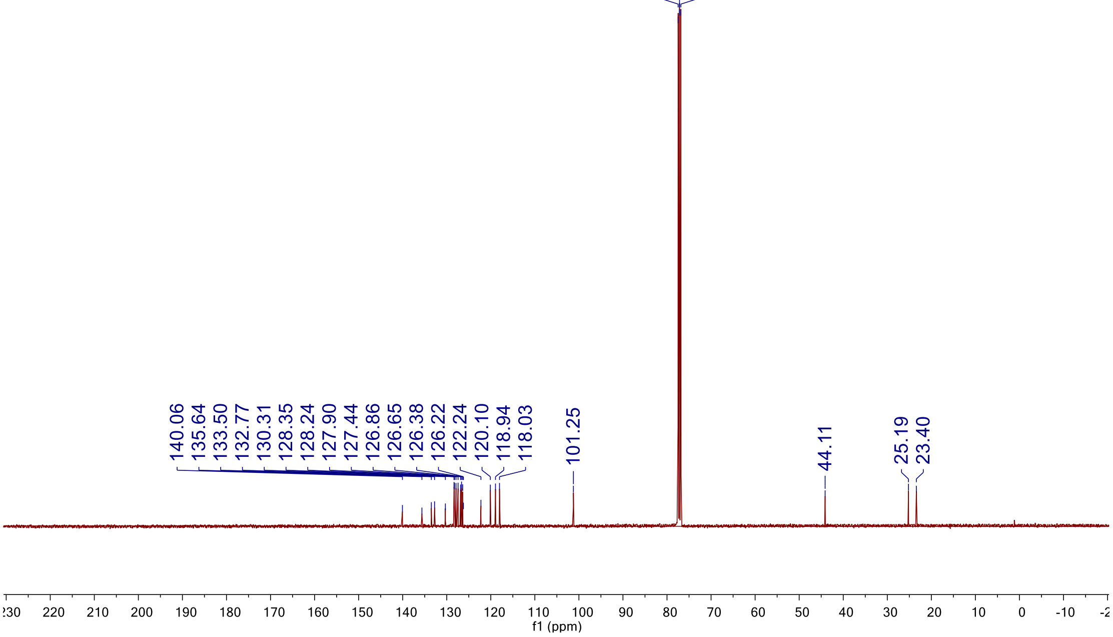

2-(Pyridin-3-yl)-5,6-dihydropyrrolo[3,2,1-*ij*]quinoline (16)

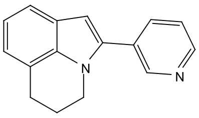

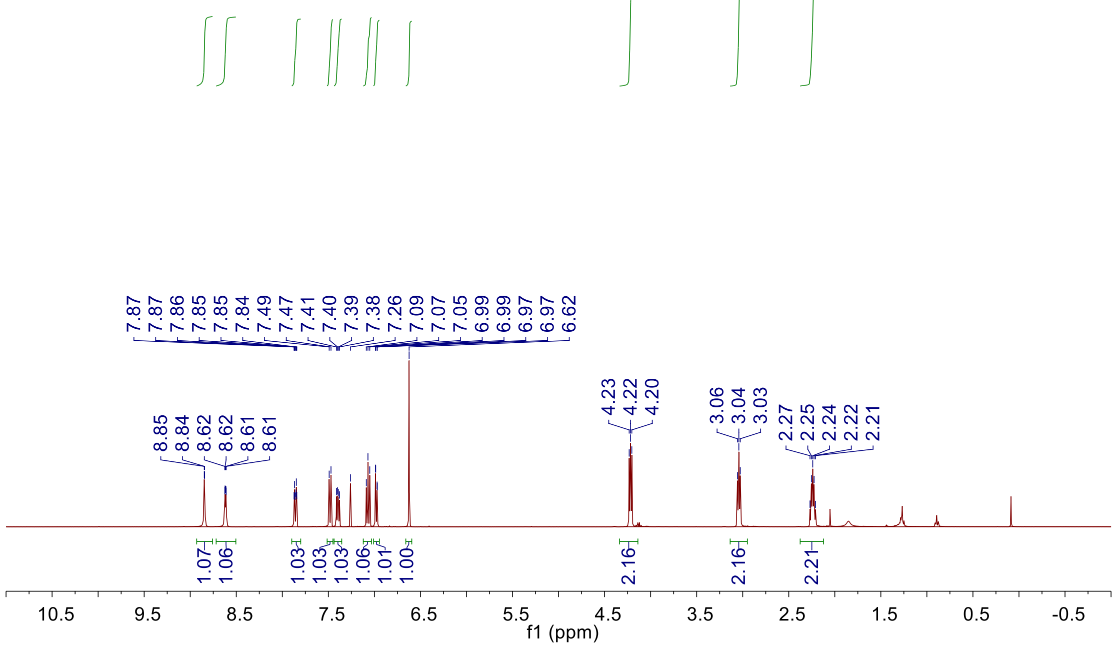

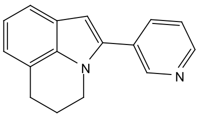

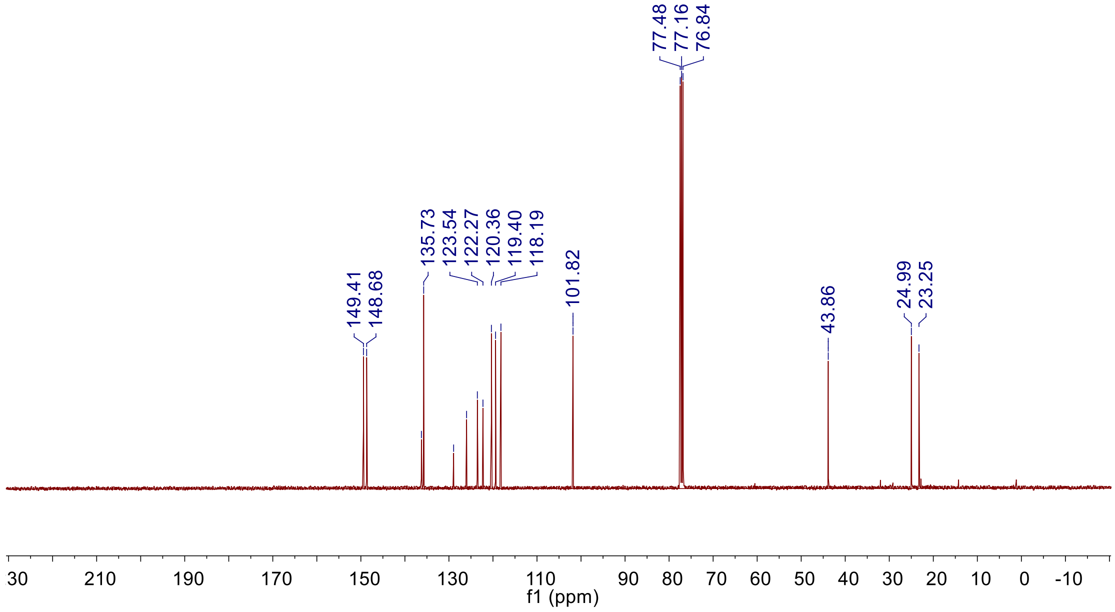

Other regioisomer: 1-(Pyridin-3-yl)-5,6-dihydropyrrolo[3,2,1-*ij*]quinoline

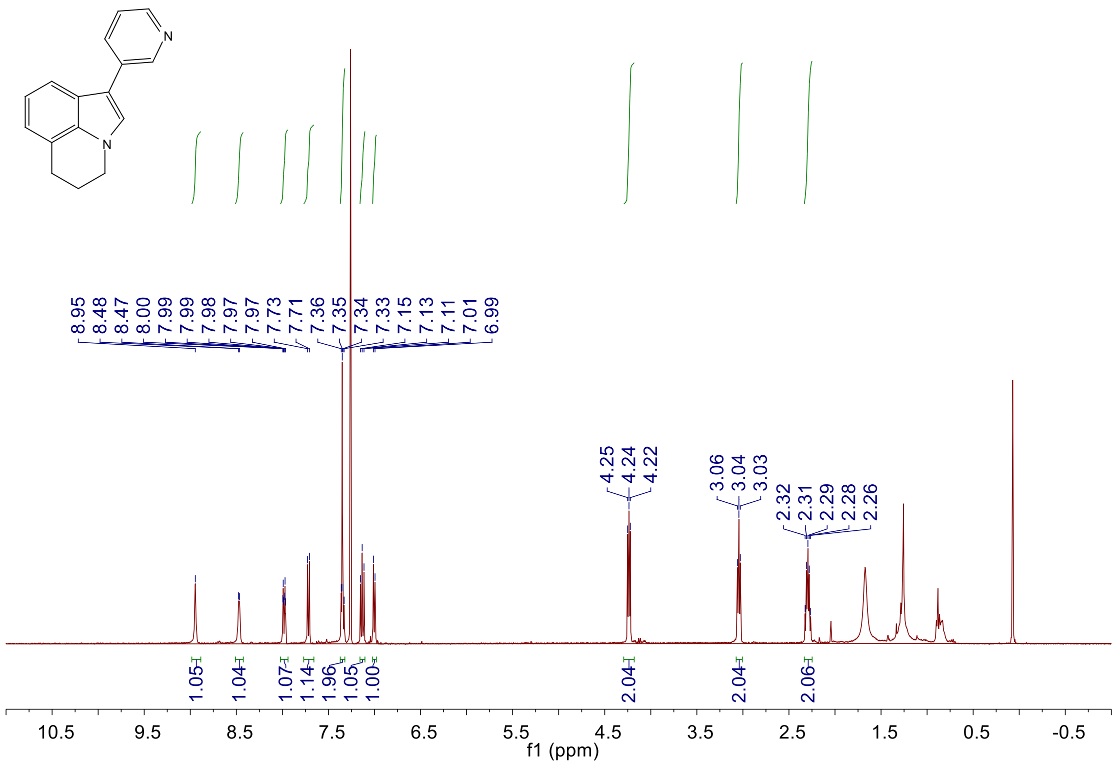

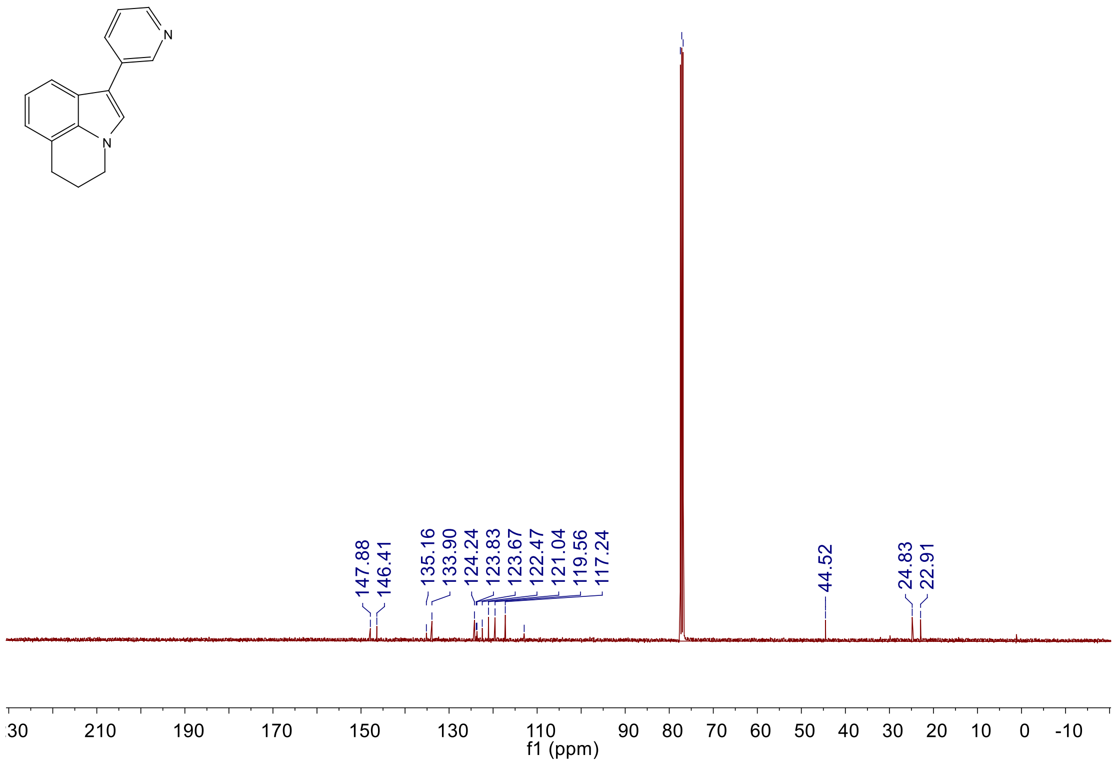

2-(Pyridin-4-yl)-5,6-dihydropyrrolo[3,2,1-*ij*]quinoline (17)

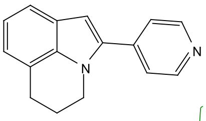

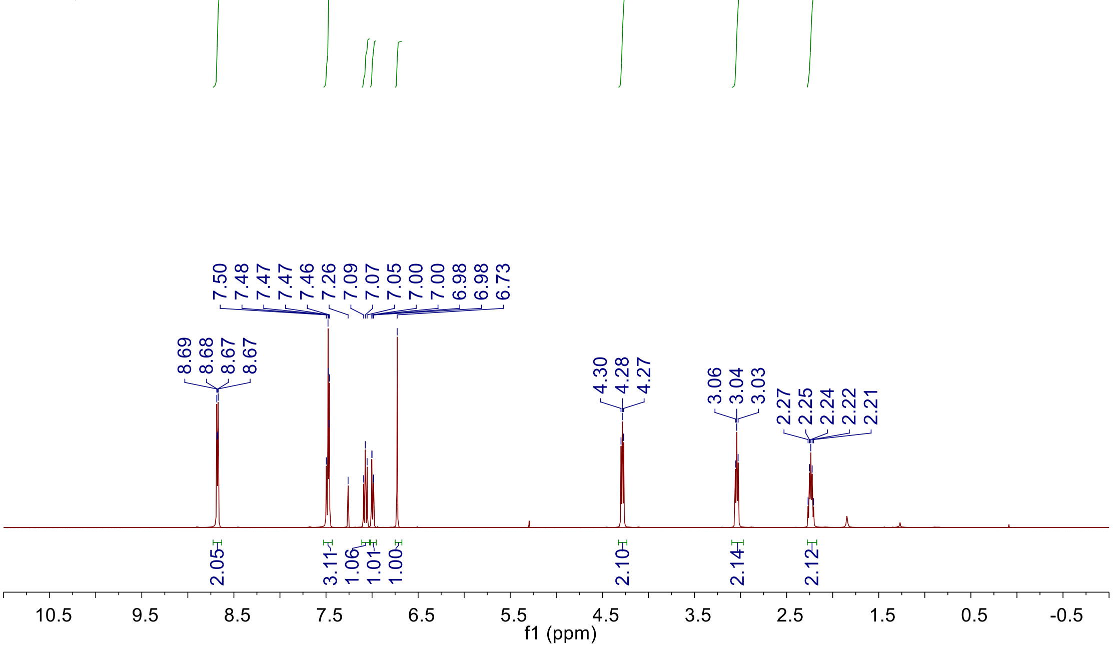

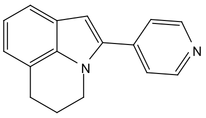

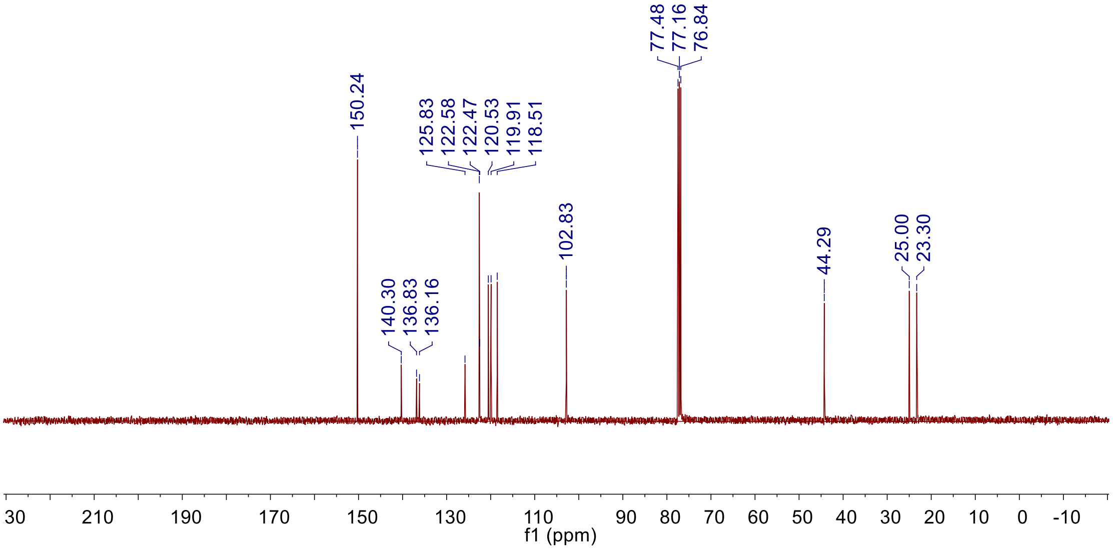

2-(Quinolin-3-yl)-5,6-dihydropyrrolo[3,2,1-*ij*]quinoline (18)

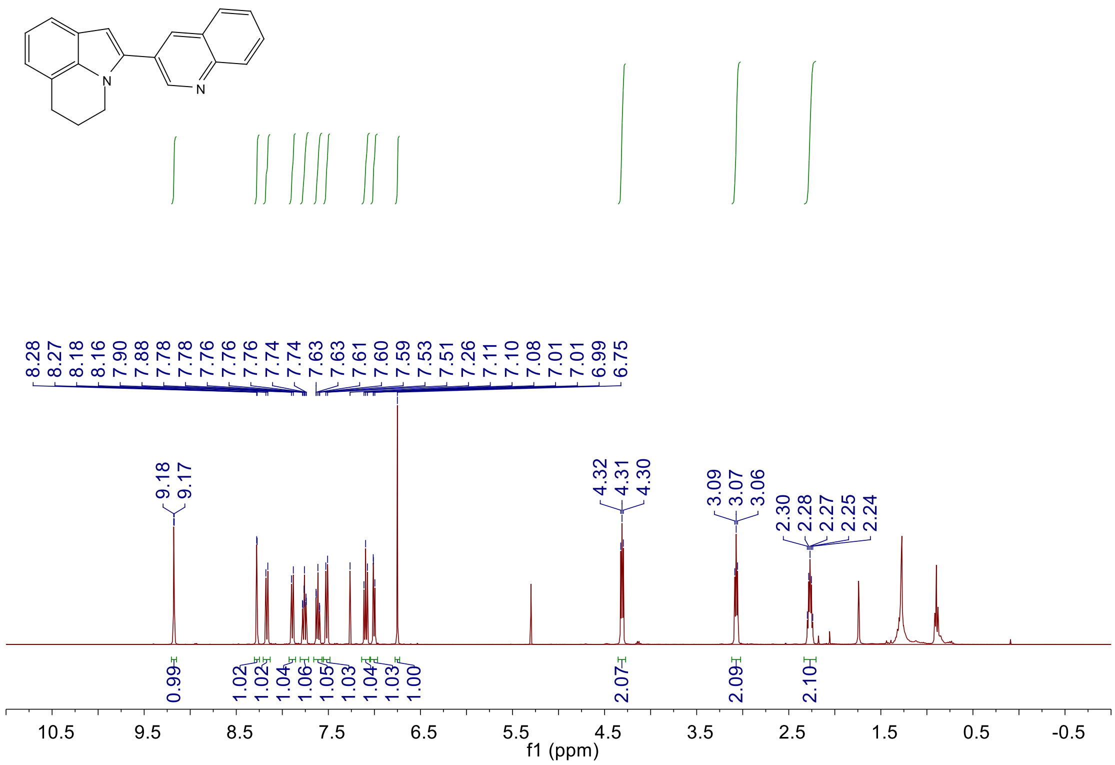

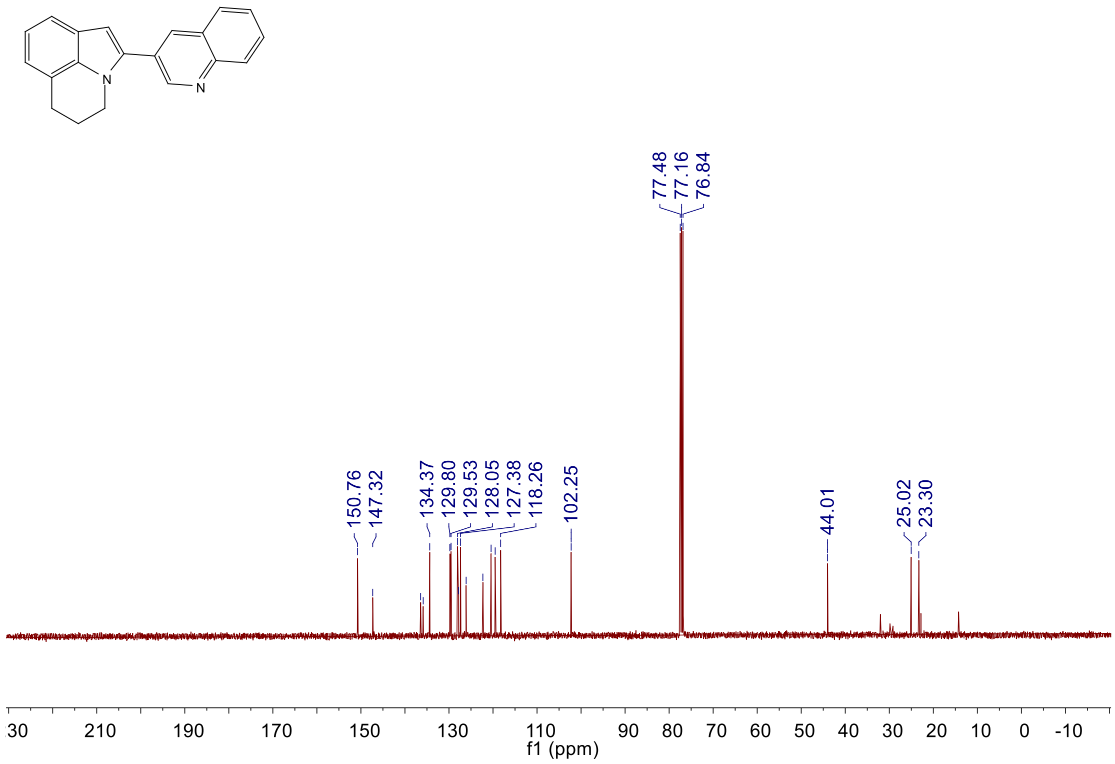

2-(Isoquinolin-4-yl)-5,6-dihydropyrrolo[3,2,1-*ij*]quinoline (19)

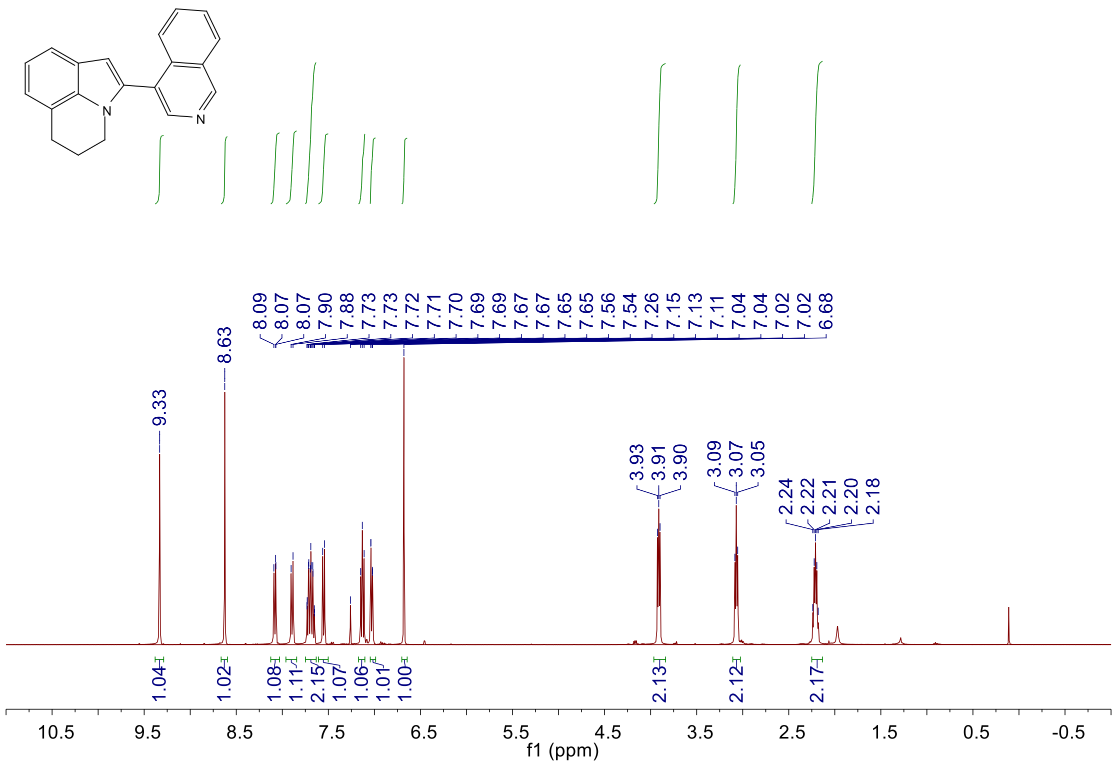

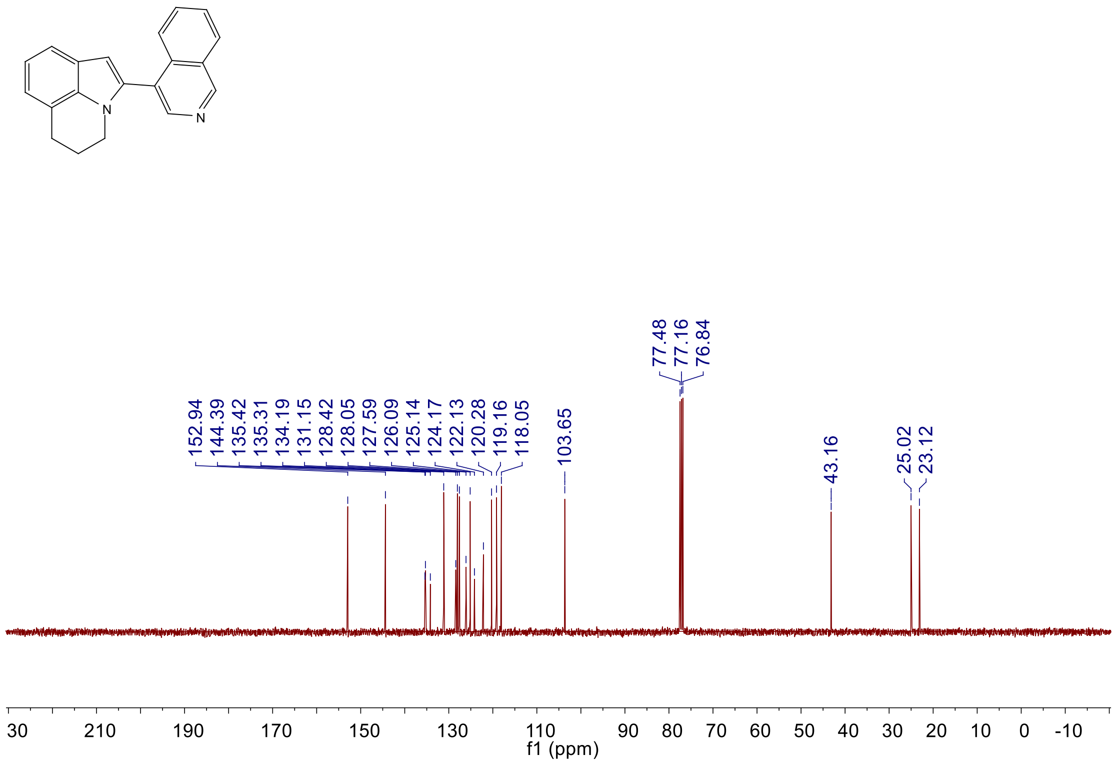

Other regioisomer: 1-(Isoquinolin-4-yl)-5,6-dihydropyrrolo[3,2,1-*ij*]quinoline

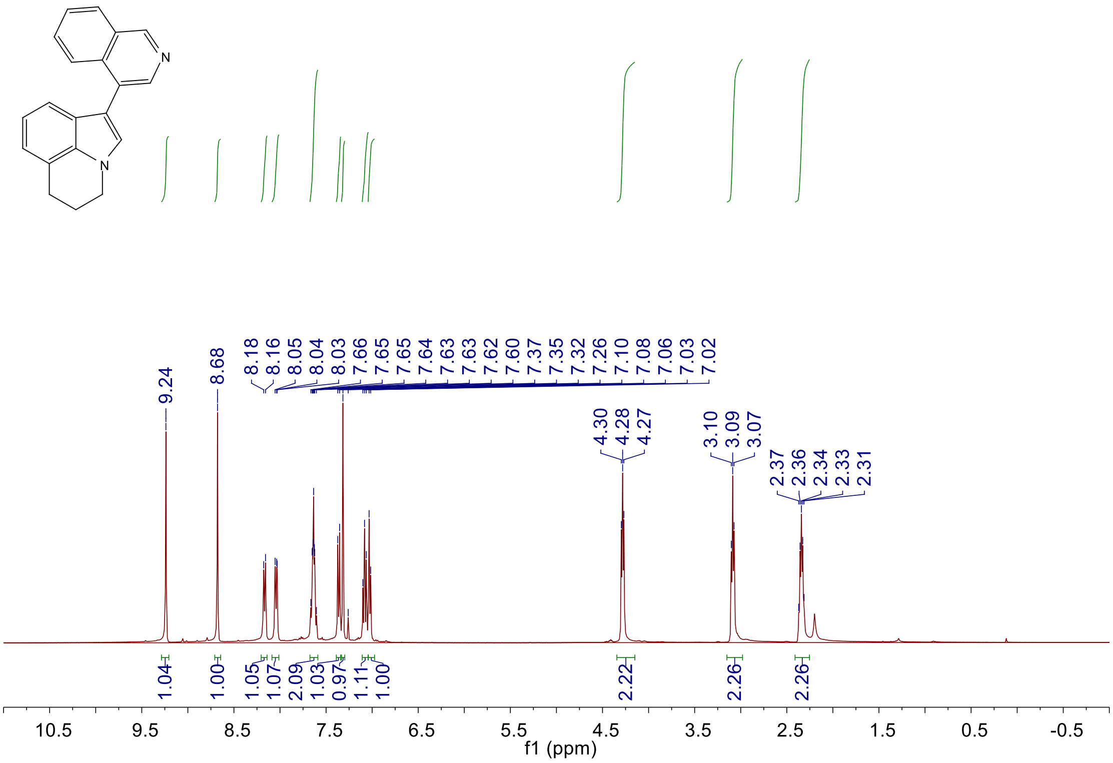

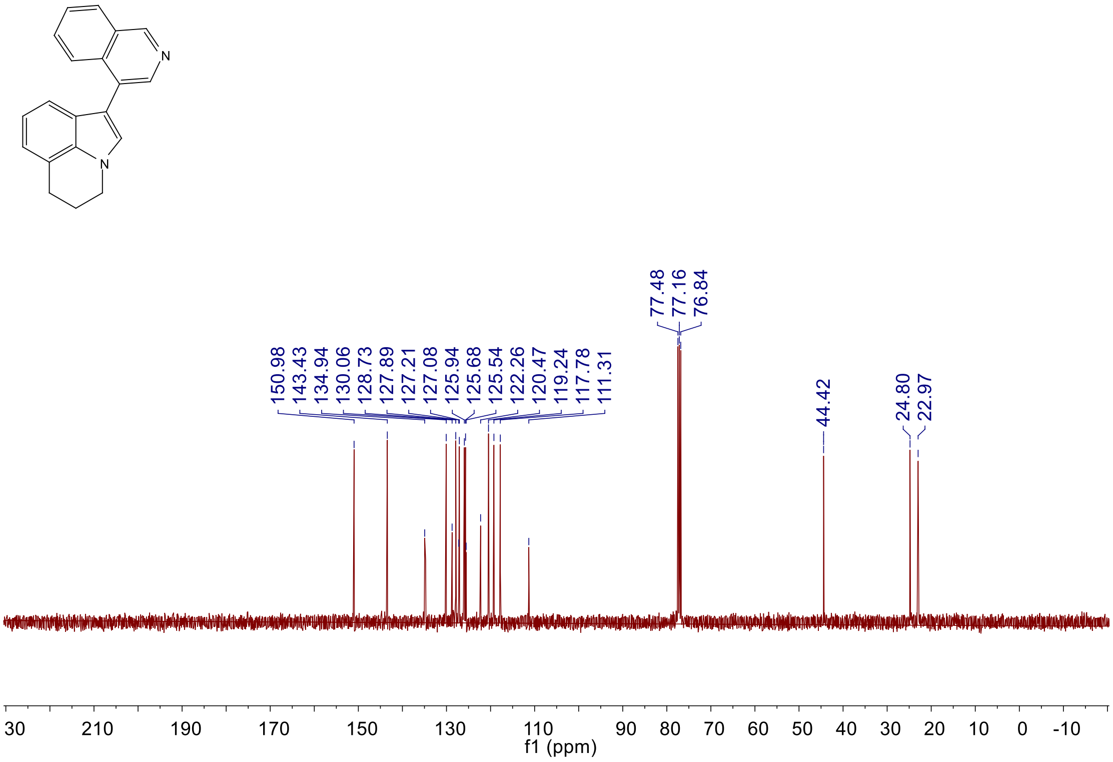

1,2-Bis(4-fluorophenyl)-5,6-dihydropyrrolo[3,2,1-*ij*]quinoline (20)

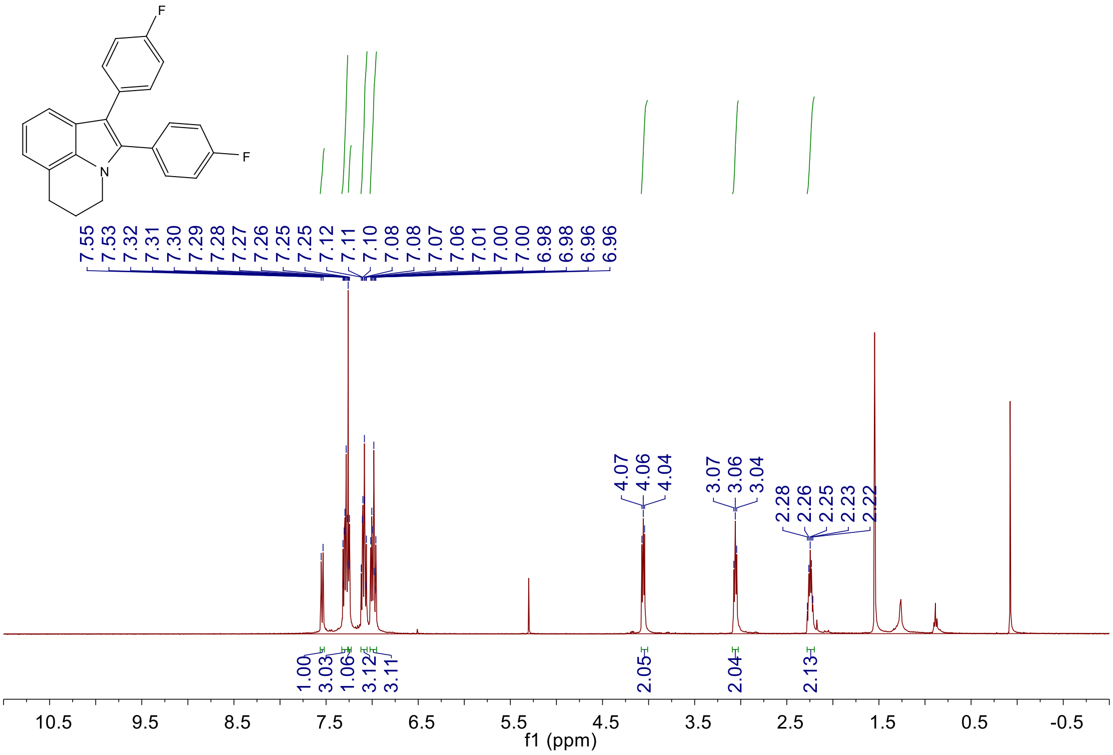

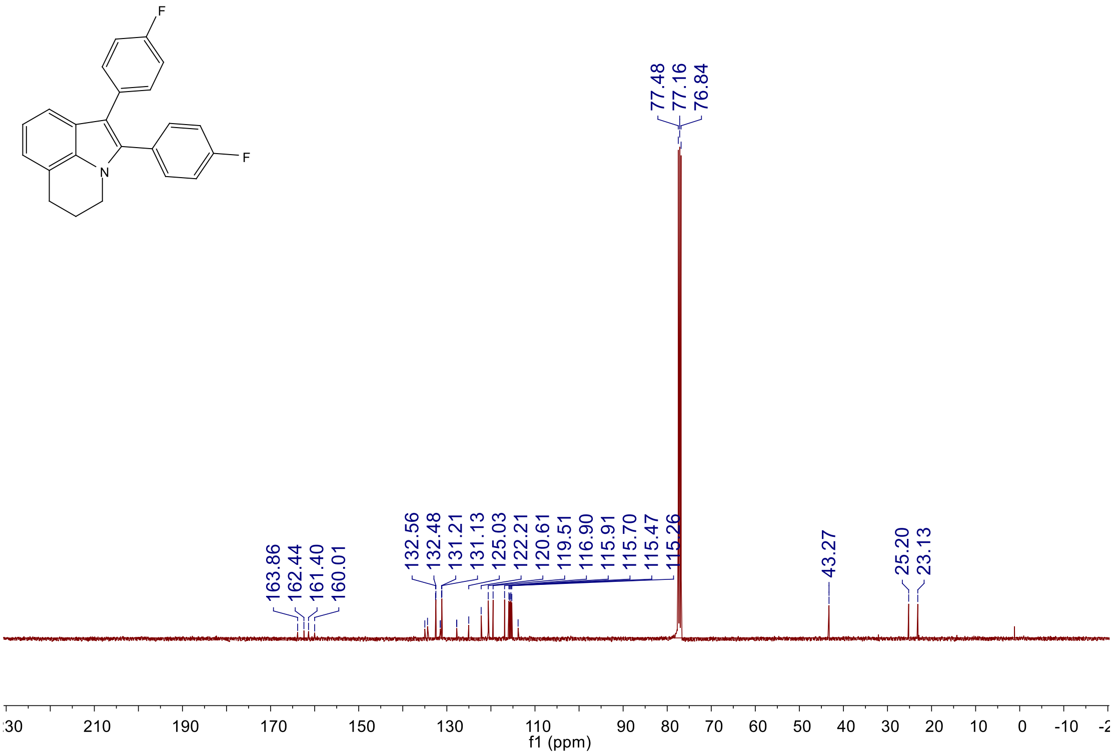

1,2-Bis(4-(trifluoromethyl)phenyl)-5,6-dihydropyrrolo[3,2,1-*ij*]quinoline (21)

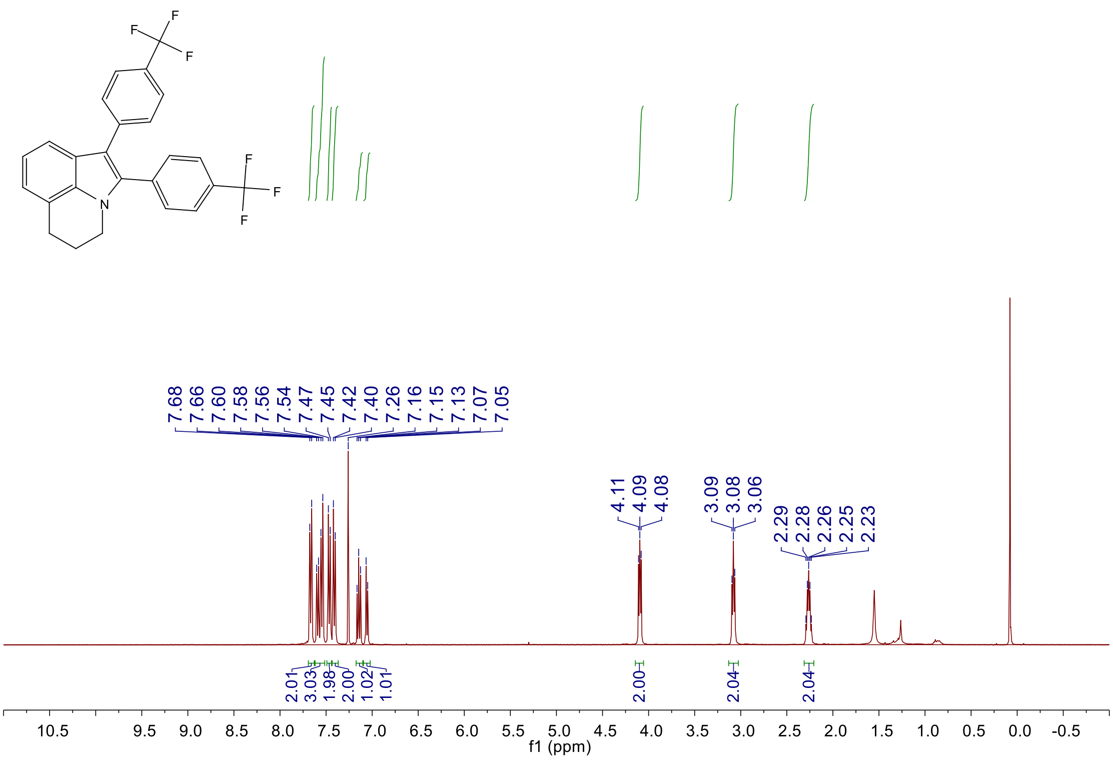

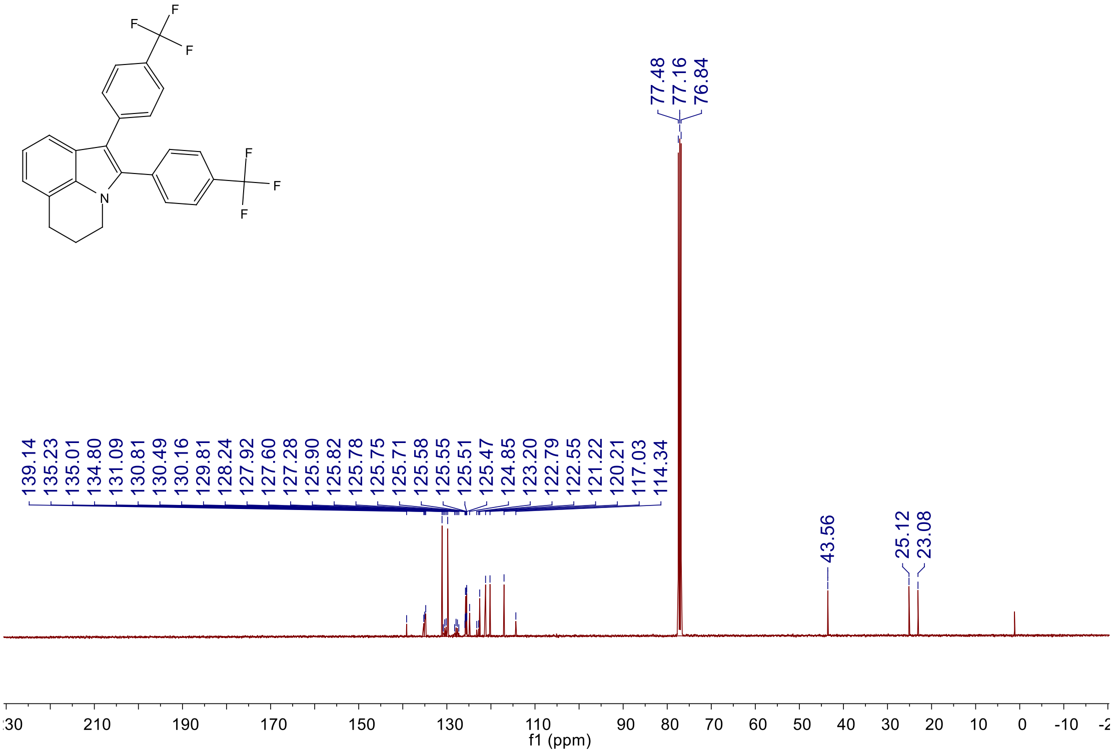

1,2-Bis(6-(trifluoromethyl)pyridin-2-yl)-5,6-dihydropyrrolo[3,2,1-*ij*]quinoline (22)

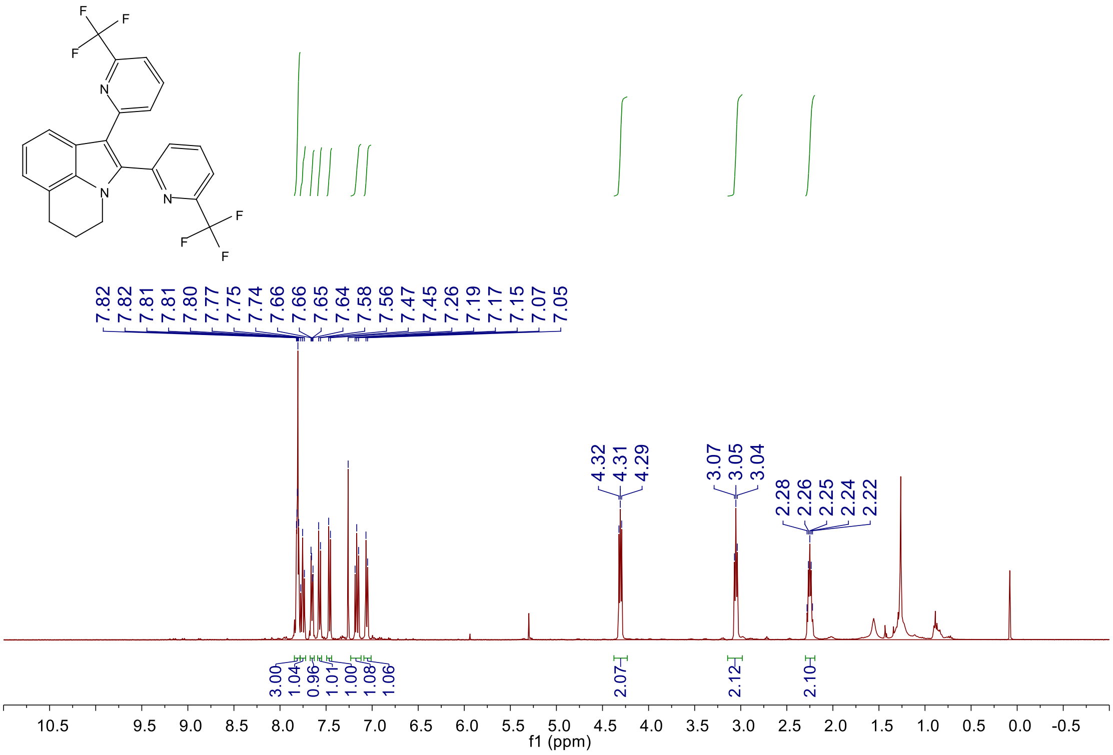

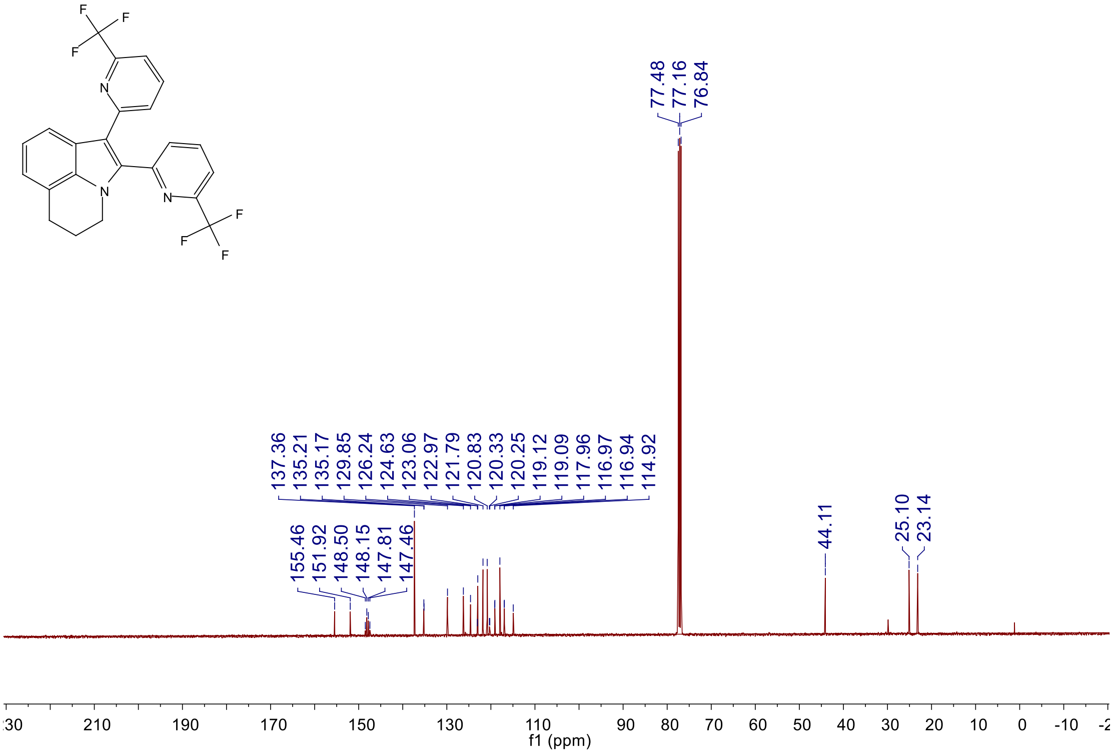

4-(1-(4-Acetylphenyl)-5,6-dihydropyrrolo[3,2,1-*ij*]quinolin-2-yl)benzonitrile (23)

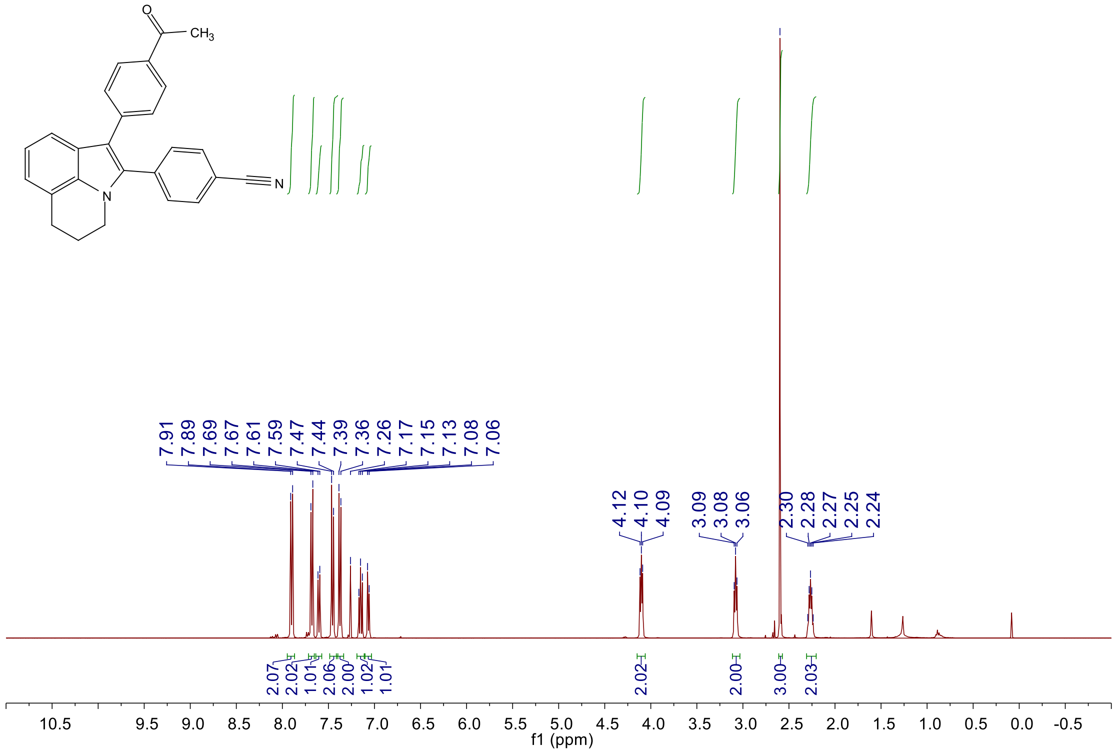

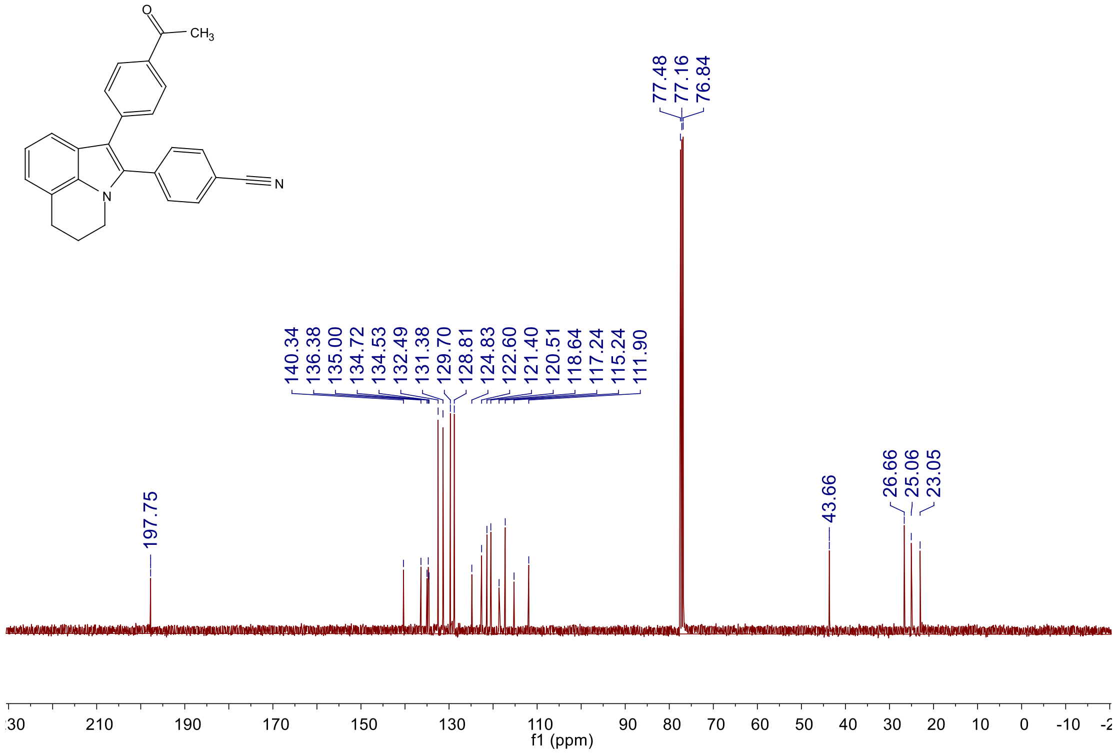

4-(1-(4-(Trifluoromethyl)phenyl)-5,6-dihydropyrrolo[3,2,1-*ij*]quinolin-2-yl)benzonitrile (24)

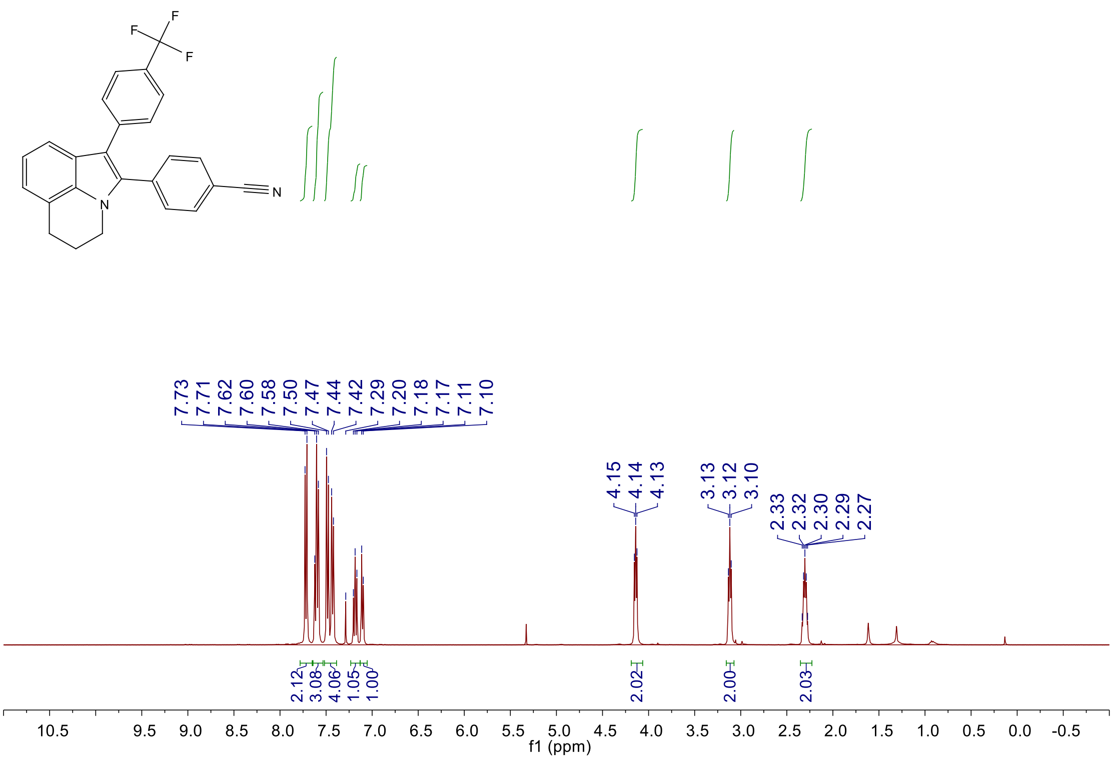

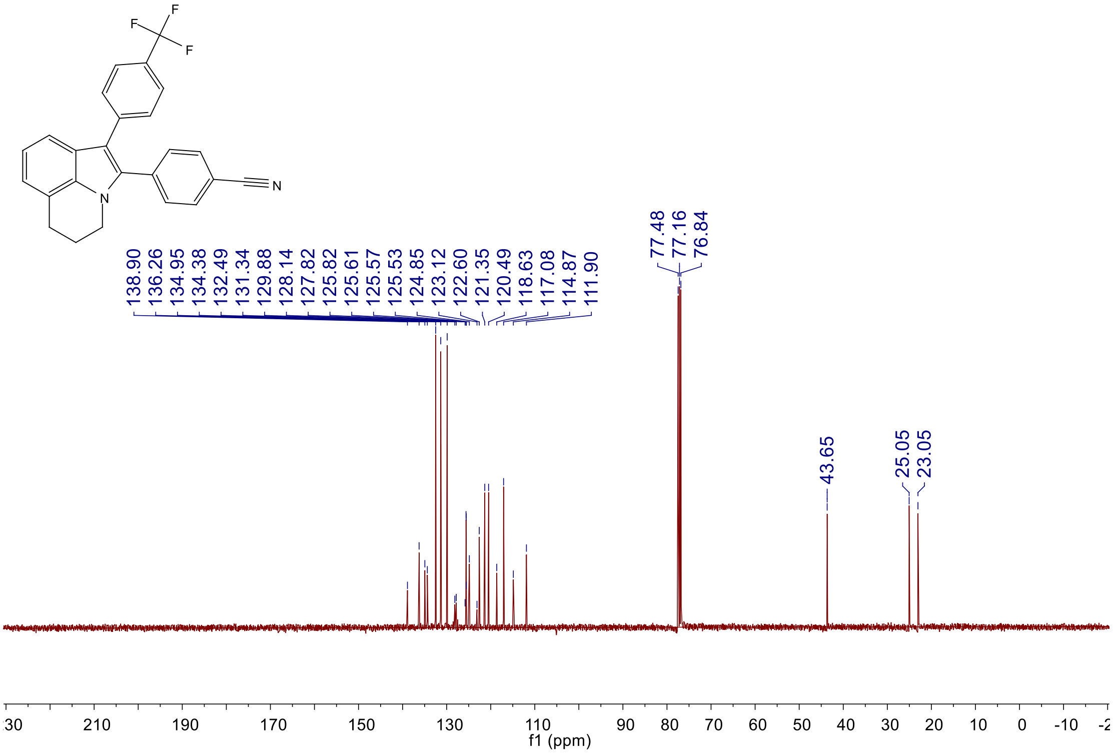

4-(1-(3,5-Bis(trifluoromethyl)phenyl)-5,6-dihydropyrrolo[3,2,1-*ij*]quinolin-2-yl)benzonitrile (25)

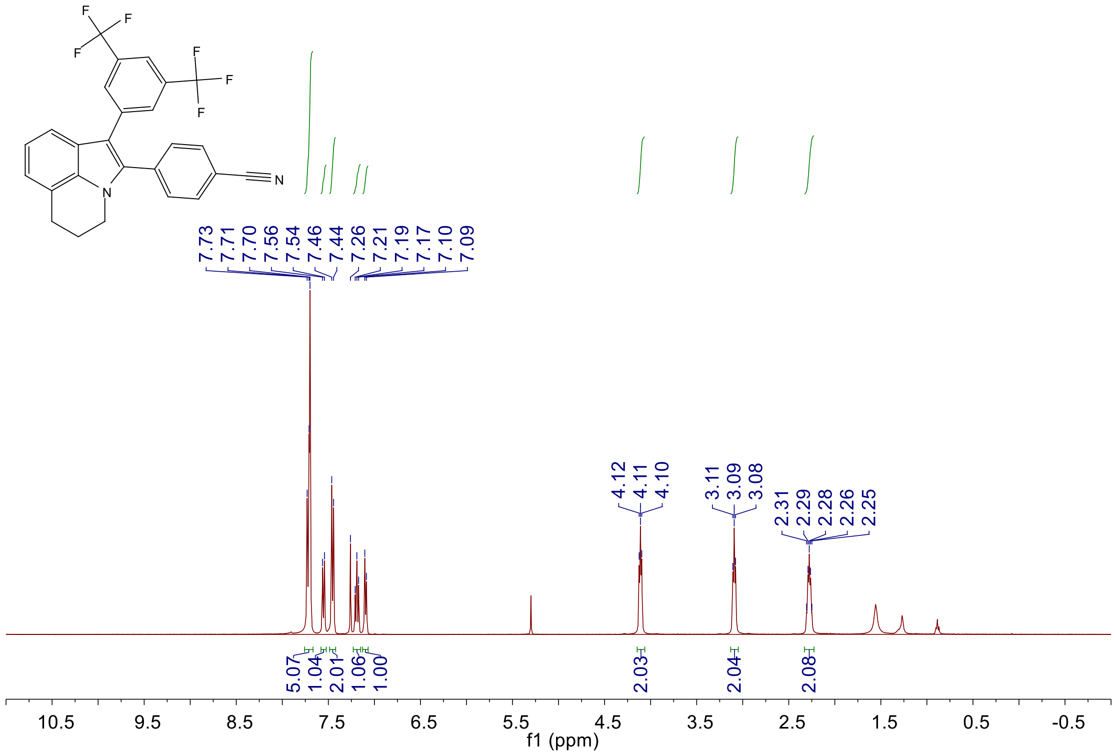

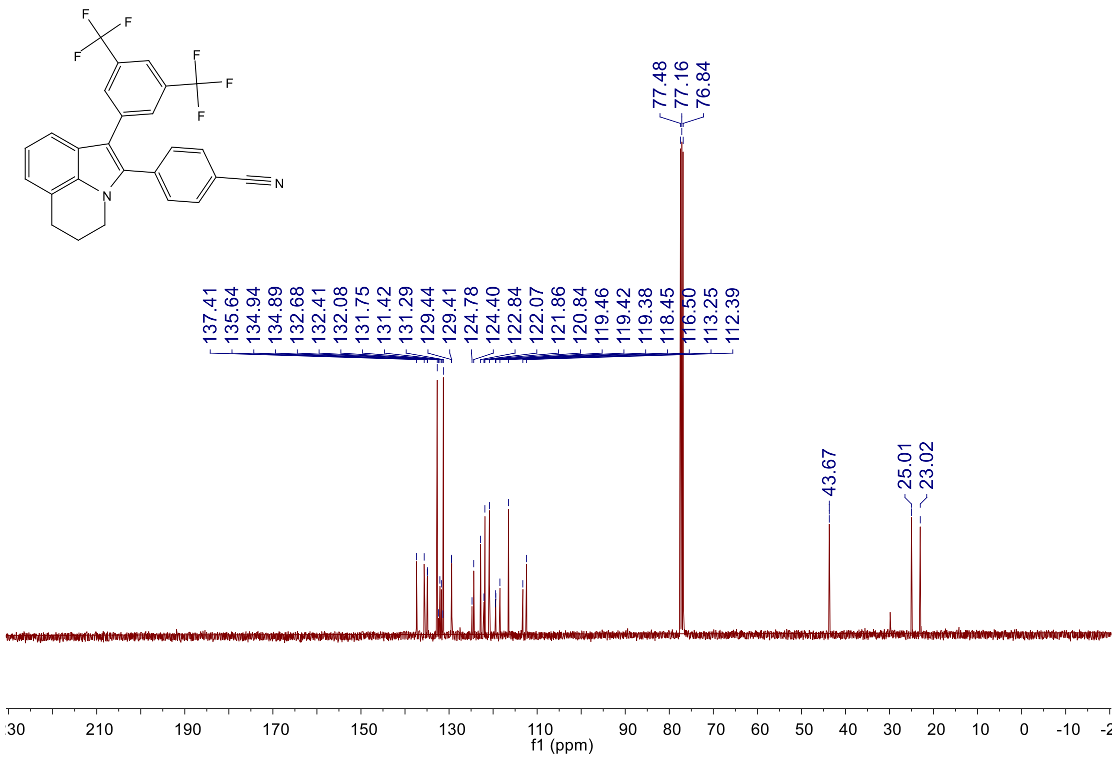

3-(2-(4-Cyanophenyl)-5,6-dihydropyrrolo[3,2,1-*ij*]quinolin-1-yl)benzonitrile (26)

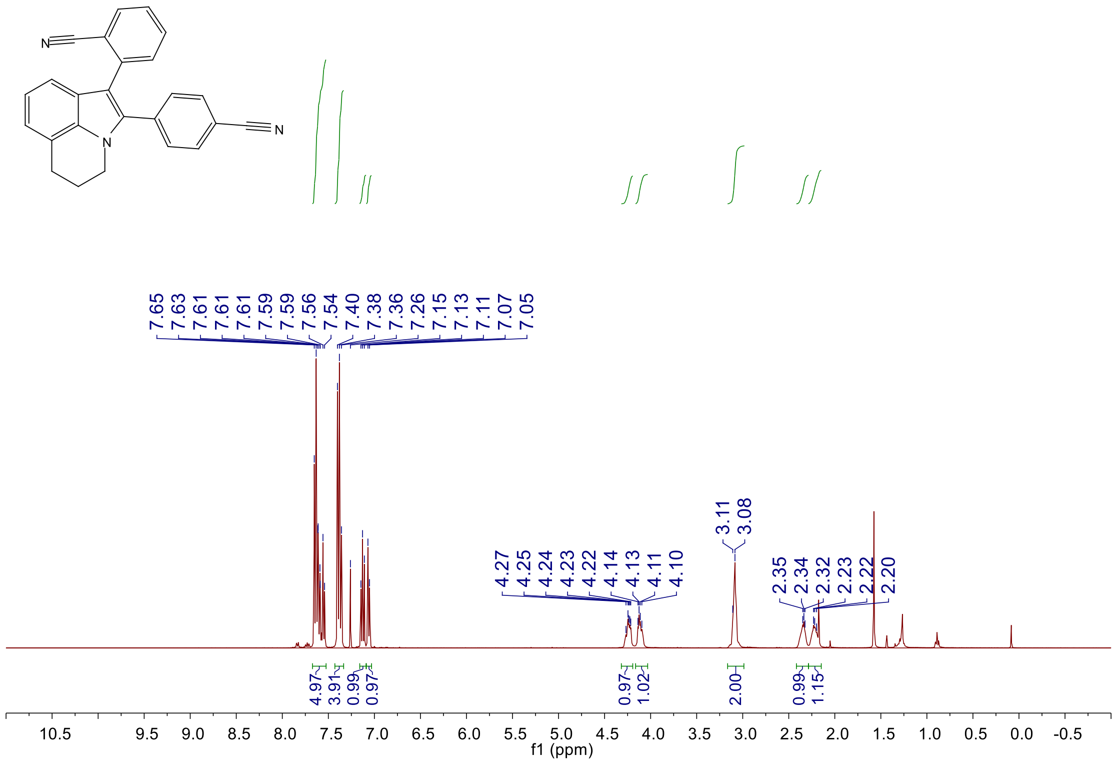

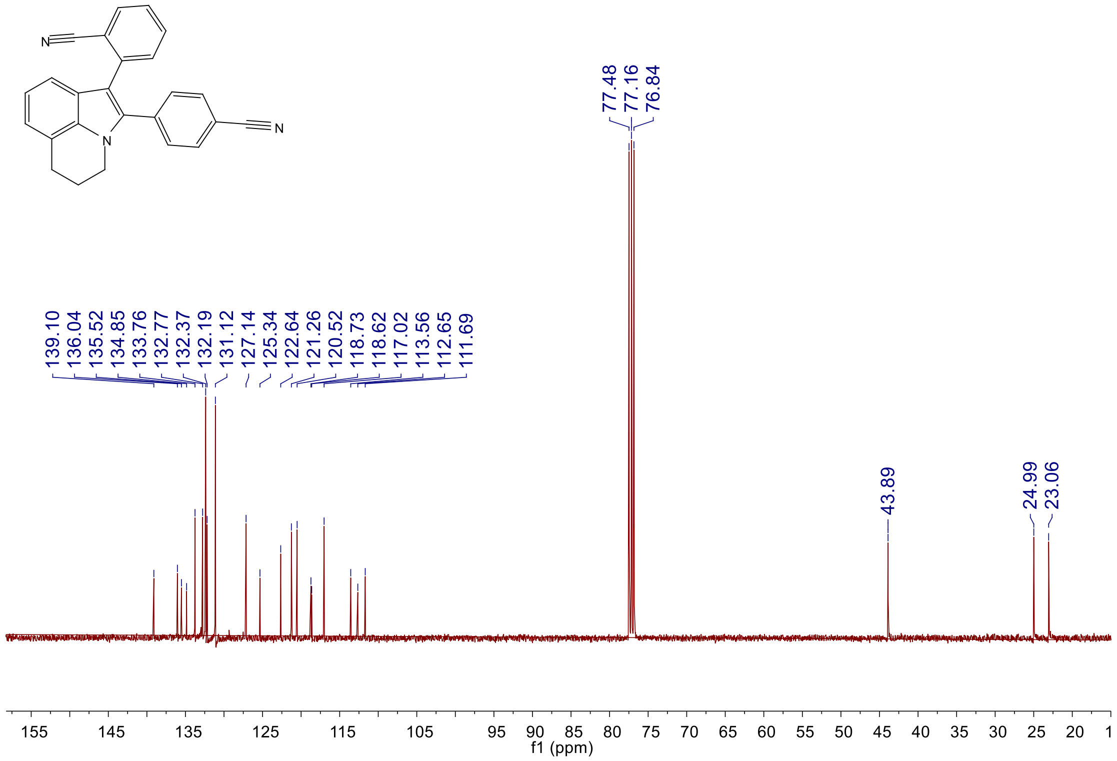

5,6-Dihydro-4*H*-dibenzo[*a*,*c*]pyrido[3,2,1-*jk*]carbazole-10-carbonitrile (27)

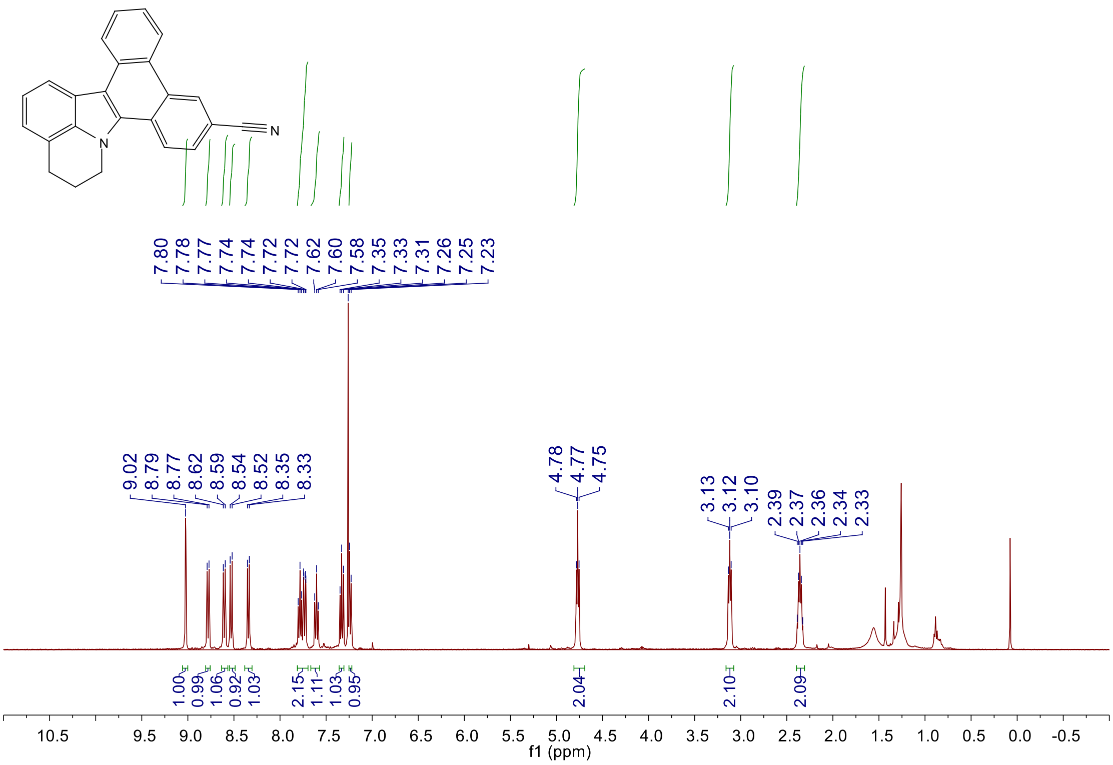

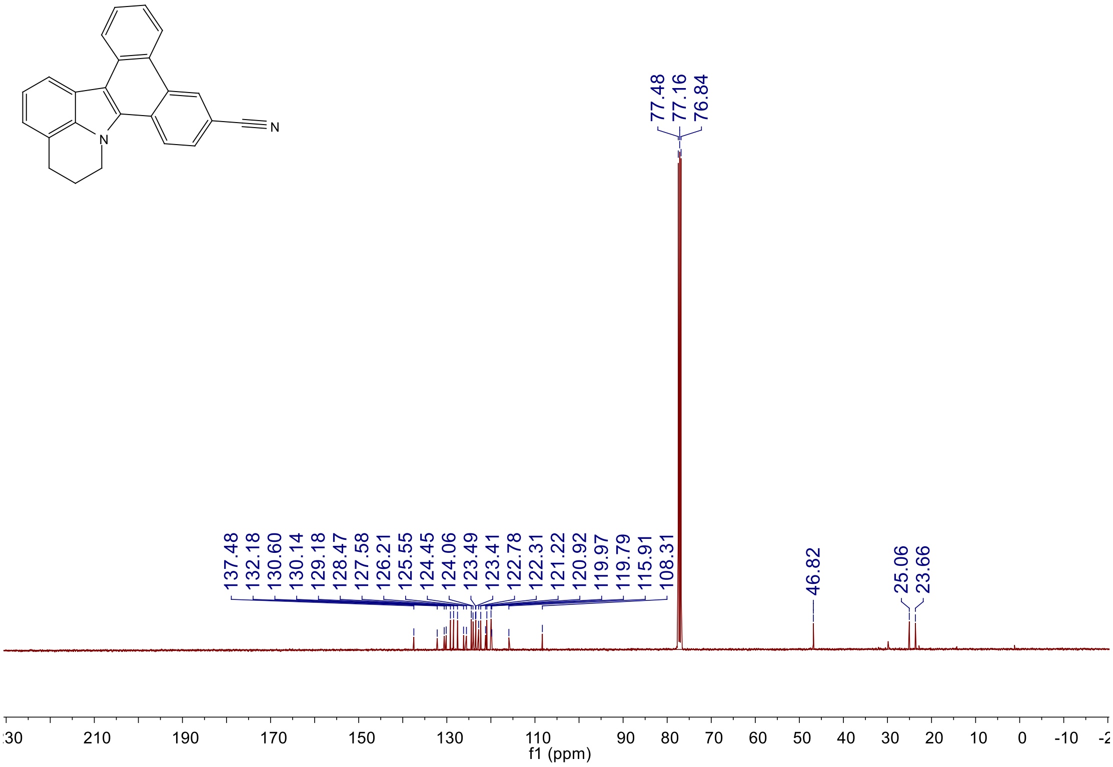

(5,6-Dihydro-4*H*-dibenzo[*a*,*c*]pyrido[3,2,1-*jk*]carbazol-10-yl)(phenyl)methanone (28)

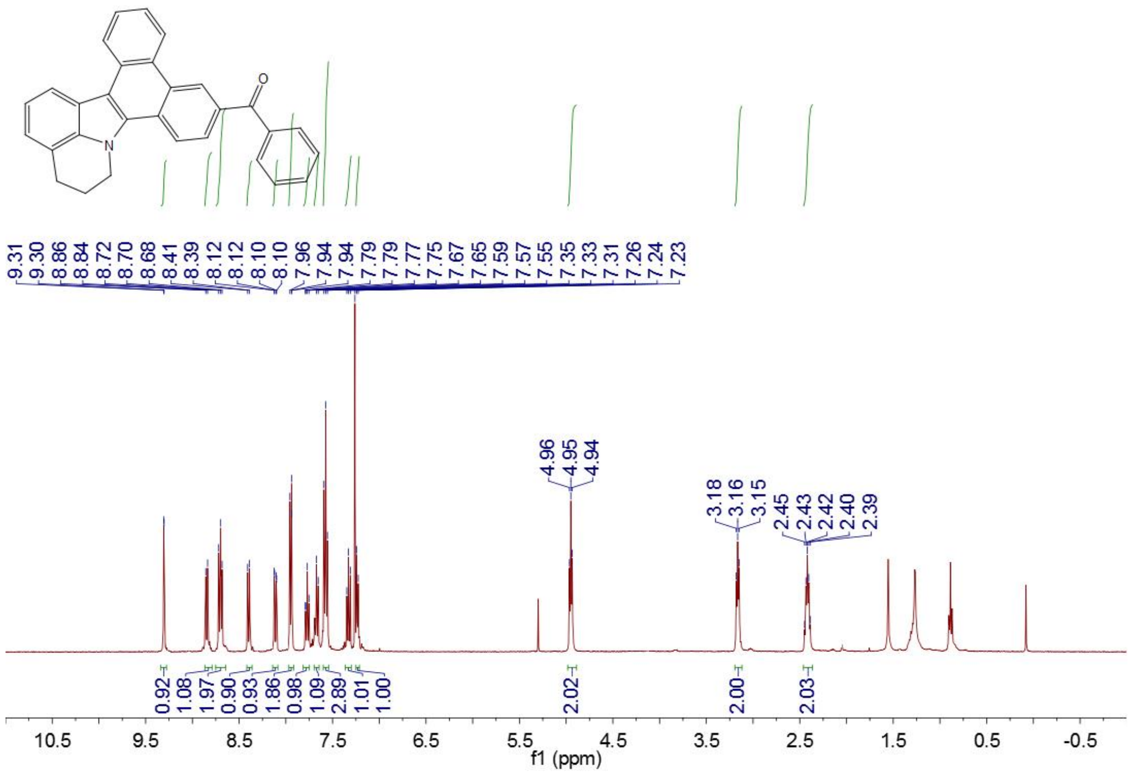

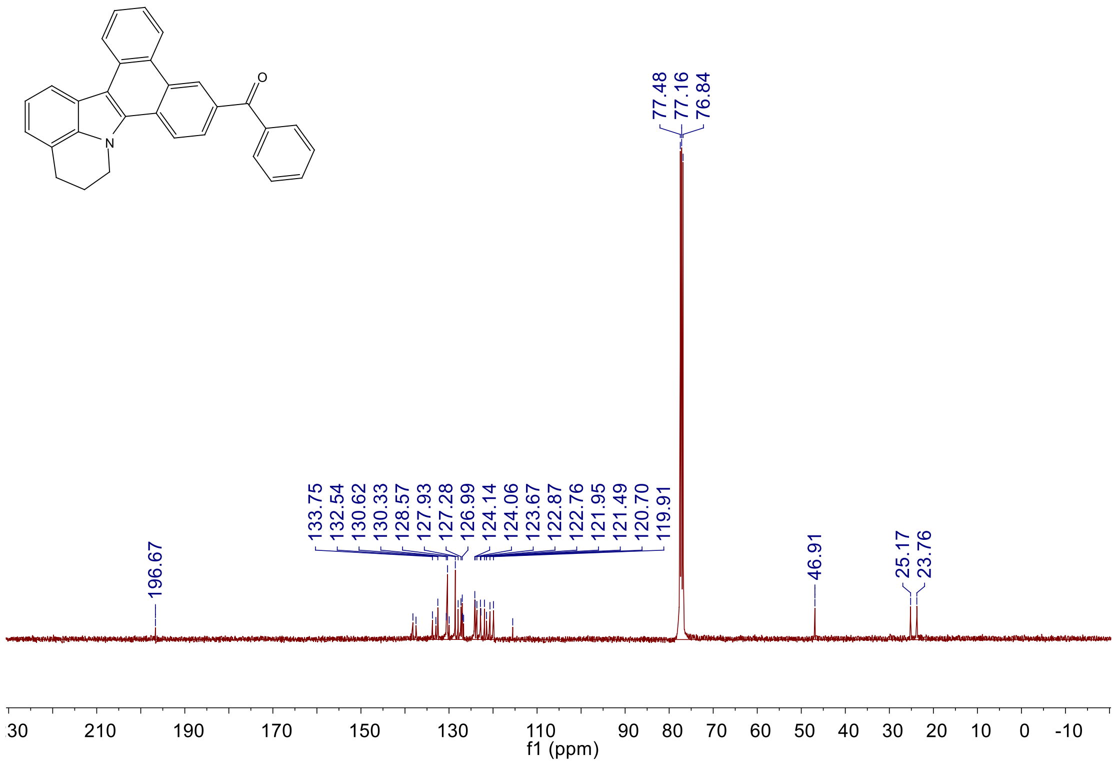

13,14-Dihydro-12*H*-benzo[*c*]dipyrido[4,3-*a*:3',2',1'-*jk*]carbazole (29)

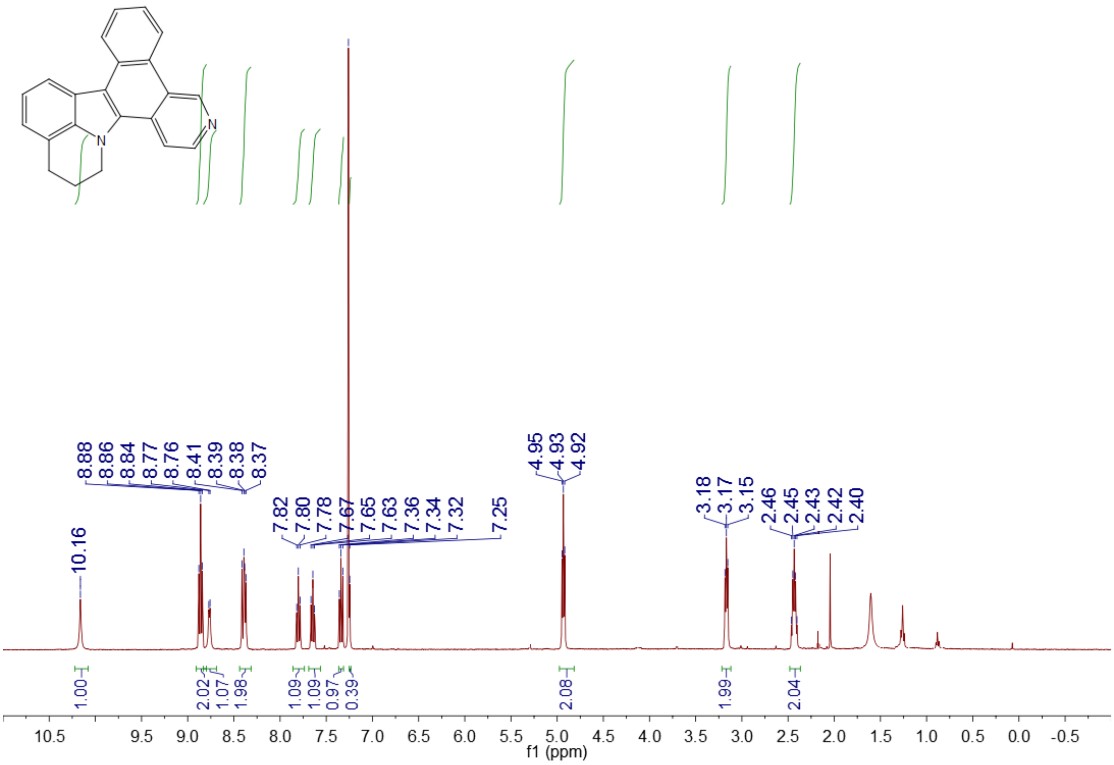

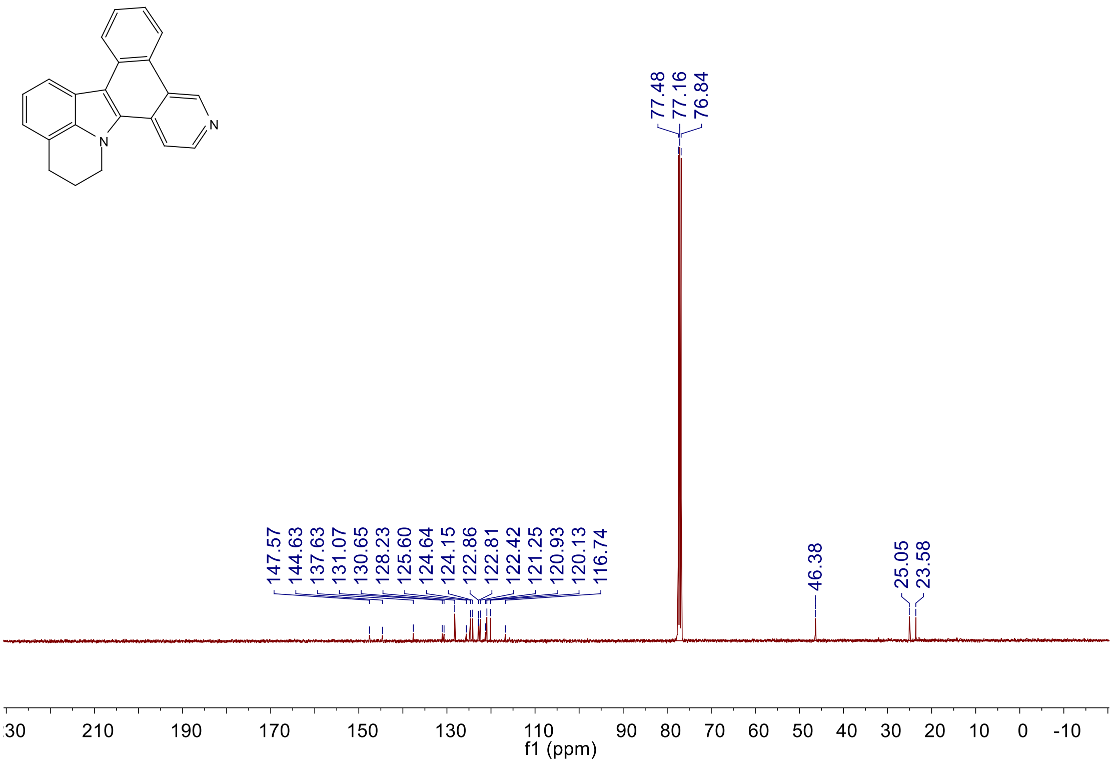

CCDC numbers of products 2, 20 and 23:

For 4-(5,6-dihydropyrrolo[3,2,1-*ij*]quinolin-2-yl)benzonitrile (**2**): CCDC number 1904054; for 1,2-bis(4-fluorophenyl)-5,6-dihydropyrrolo[3,2,1-ij]quinoline (**20**): CCDC 1909150; for 4-(1-(4-acetylphenyl)-5,6-dihydropyrrolo[3,2,1-*ij*]quinolin-2-yl)benzonitrile (**23**): CCDC number 1904056.
